# Amphiphilic nebramine analogs synergize with β-lactam/β-lactamase inhibitor combinations, including cefepime–taniborbactam and meropenem–xeruborbactam against metallo-β-lactamase-carrying *Pseudomonas aeruginosa*†

**DOI:** 10.1039/d5md00375j

**Published:** 2025-07-17

**Authors:** Christian Lozeau, Danzel Ramirez, Danyel Ramirez, Gulshan Kumar, Rajat Arora, George Zhanel, Gilbert Arthur, Frank Schweizer

**Affiliations:** a Department of Chemistry, University of Manitoba Winnipeg MB R3T 2N2 Canada lozeauc@myumanitoba.ca ramirezd@myumanitoba.ca ramiredm@myumanitoba.ca gulshan.kumar@umanitoba.ca arorar5@myumanitoba.ca frank.schweizer@cc.umanitoba.ca; b Department of Medical Microbiology and Infectious Diseases, University of Manitoba Winnipeg MB R3E 0J9 Canada ggzhanel@pcsinternet.ca; c Department of Biochemistry and Medical Genetics, University of Manitoba Winnipeg MB R3E 0J9 Canada gilbert.arthur@umanitoba.ca

## Abstract

Cefepime–taniborbactam (FEP–TAN) and meropenem–xeruborbactam (MEM–XER) are β-lactam–β-lactamase inhibitor (BL–BLI) combinations currently in development and both projected to treat metallo-β-lactamase (MBL)-producing Gram-negative pathogens. Among Gram-negative pathogens, the low permeability of the outer membrane of *Pseudomonas aeruginosa* poses unique challenges to drug discovery in general and to BL–BLIs in particular. This study set out to augment β-lactam antibiotic potency by enhancing outer membrane permeability of *P. aeruginosa* using novel amphiphilic aminoglycoside-based outer membrane permeabilizers. Amphiphilic nebramines acting as outer membrane permeabilizers, were synthesized and evaluated in combination with β-lactam antibiotics and BL–BLIs against *P. aeruginosa* clinical isolates harbouring a number of resistance determinants, including MBLs. Dually guanidinylated and C-5-alkylated analogs of nebramine were able to sensitize MBL-carrying *P. aeruginosa* to various BL–BLIs. The amphiphilic nebramine derivative, compound 4, synergized with multiple β-lactam antibiotics and BL–BLIs including aztreonam–avibactam (ATM–AVI), FEP–TAN and MEM–XER against multidrug-resistant *P. aeruginosa* isolates. In particular, compound 4 + ATM–AVI, restored susceptibility to all nine β-lactamase (including MBL)-harbouring *P. aeruginosa* strains that were previously resistant to aztreonam. Compound 4 was found to be less toxic than both polymyxin B and its corresponding amphiphilic tobramycin counterpart (compound 7) in human renal cell lines, RPTEC and HK-2. Overall, our study suggests that addition of compound 4 alongside next-generation BL–BLIs such as FEP–TAN, MEM–XER as well as the recently approved ATM–AVI combination can overcome intrinsic and acquired *in vitro P. aeruginosa* resistance determinants that confer high-level resistance to β-lactam antibiotics.

## Abstract 2

Cefepime–taniborbactam (FEP–TAN) and meropenem–xeruborbactam (MEM–XER) are β-lactam–β-lactamase inhibitor (BL–BLI) combinations currently in development and both projected to treat metallo-β-lactamase (MBL)-producing Gram-negative pathogens.

## Graphical abstract

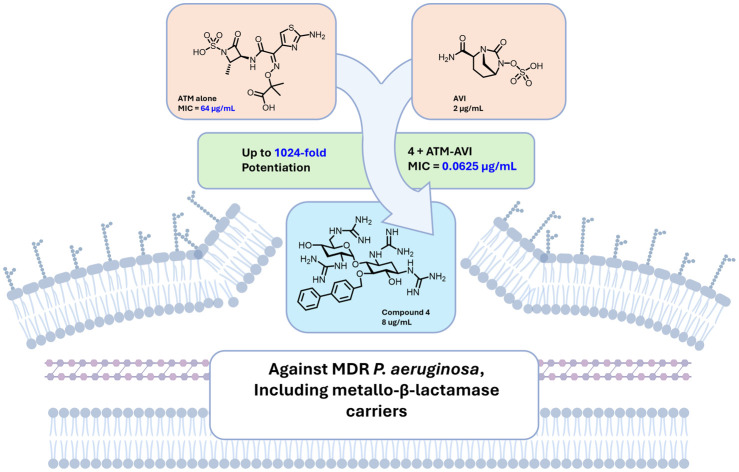

## Introduction

*Pseudomonas aeruginosa* is a difficult to treat pathogen in hospital settings in part due to its comparatively low outer membrane permeability amongst Gram-negative bacilli. Recently, the World Health Organization deemed carbapenem-resistant *P. aeruginosa* (CRPA) a high priority pathogen.^1^ CRPA are often multidrug-resistant (MDR) which means they are resistant to three or more antibiotic classes used for routine treatment. MDR *P. aeruginosa* infections can leave few or no treatment options available. Since carbapenems are an antibiotic drug class of last resort, the rise of CRPA and MDR *P. aeruginosa* is concerning. Like all β-lactam antibiotics, carbapenems mimic the d-alanyl-d-alanine dipeptide which normally binds to penicillin-binding proteins (PBPs) in peptidoglycan crosslinking.^2,3^ The binding of carbapenems to PBPs inhibit cell wall synthesis and leads to rapid lysis of the bacteria. To circumvent the action of β-lactam antibiotics, *P. aeruginosa* utilizes various resistance mechanisms including PBP modifications, multidrug efflux pumps, reduced porin expression and the overproduction of β-lactamases.^4^ β-Lactamases hydrolyze the lactam ring of the β-lactam antibiotic *via* a catalytic serine residue present in the enzyme active site.^3^ β-Lactamases are recognized widely as being classified by amino acid sequence into classes A through D by the Ambler system.^5,6^ The earliest β-lactamase inhibitor (BLI) is the natural product clavulanic acid, which was originally partnered with the aminopenicillin, amoxicillin.^7^ Later, other 1st generation BLIs appeared including penicillanic acid sulfones: sulbactam and tazobactam.^8,9^ It was not until 2015 that 2nd generation broader-spectrum inhibitors, such as the diazabicyclooctane, avibactam (AVI), were approved. AVI is able to inhibit the intrinsic Ambler class C *Pseudomonas*-derived cephalosporinase (PDC).^10^ PDC is the major inducible cephalosporinase contributing to β-lactam resistance and is encoded by the chromosomal ampC gene.^10–12^ Recently, β-lactamases of a significantly different type (class B), called metallo-β-lactamases (MBLs), are gaining research spotlight.^13^ MBLs are a major resistance factor that pose a formidable treatment obstacle in *P. aeruginosa* infections. Alarmingly, MBLs have broad substate specificity, with aztreonam (ATM) being the only β-lactam antibiotic resistant to hydrolysis.^14^ MBLs carry either one (class B2) or two zinc centres (class B1 and B3) in their active site which house a catalytic hydroxide to initiate the attack on the carbonyl carbon within the β-lactam ring.^15^ Currently, there are no FDA-approved BLIs for this class of enzyme. However, there has been a push within the last decade to develop small molecules with the capability to inhibit even MBLs, such as the bicyclic boronates taniborbactam (TAN; formerly VNRX-5133) and xeruborbactam (XER; formerly QPX-7728).^16,17^ Cefepime (FEP) used in combination with TAN has recently cleared phase III clinical trials and is awaiting FDA approval. On the other hand, XER has cleared phase I studies in combination with meropenem (MEM) and was reported to be safe and well tolerated in healthy adults.^18^ A notable advantage of TAN and XER over other BLIs, is their ability to inhibit most clinically relevant class B1 MBLs with *K*_i_ values in the nanomolar range.^18^ Another anti-MBL combination is aztreonam–avibactam (ATM–AVI). ATM avoids the fate of MBLs but is still prone to hydrolysis by extended-spectrum β-lactamases in class A, C and D, of which AVI can conveniently inhibit (except OXA-23).^19^ ATM–AVI is currently approved in Europe for complicated urinary tract infections and hospital-acquired pneumonia and is awaiting FDA approval (expected in June 2025).^20^ Unfortunately, the potency of all β-lactam–β-lactamase inhibitor (BL–BLI) combinations in *P. aeruginosa* can be compromised by reduced expression of porins and overproduction of multidrug efflux systems, such as MexAB–OprM.^17,21,22^ Therefore, a permeability enhancer such as an outer membrane permeabilizer that can increase the intracellular accumulation of BL–BLIs may be useful, even in MBL-producing strains.

**Fig. 1:**
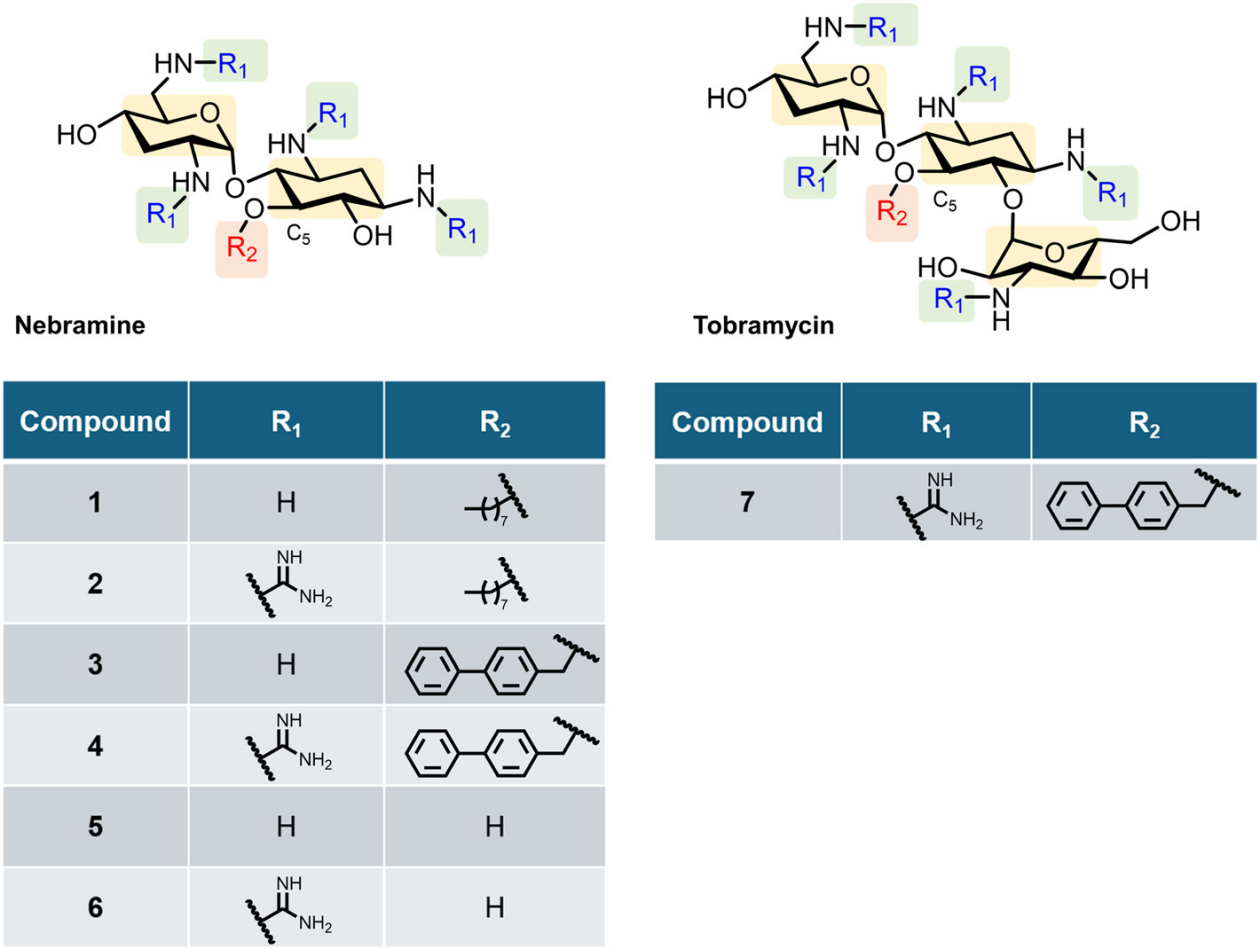
Chemical structure of the amphiphilic nebramine derivatives 1–6 and previously studied lead compound 7.

Our group has explored amphiphilic aminoglycoside-based outer membrane permeabilizers capable of synergizing with BL–BLIs against *P. aeruginosa*.^23–27^ Recently, both guanidinylation of the amino functions and *O*-alkylation to covalently attach various hydrophobic groups at the C-5 position of tobramycin was explored.^27^ The modifications afforded dual-modified amphiphilic tobramycin that could synergize with ceftazidime–avibactam (CAZ–AVI) and ATM–AVI, in MDR and β-lactamase-harbouring *P. aeruginosa*.^27^ However, tobramycin bears considerable cationicity at physiological pH due to its five amino groups, and is therefore known to be ototoxic and nephrotoxic.^28,29^ Nebramine is a pseudo-disaccharide segment of tobramycin missing the amino sugar, kanosamine (Fig. 1). Modified nebramine analogs have shown the potential to retain synergy with β-lactam antibiotics and offer reduced cytotoxic properties.^30–32^ This property is rationalized by one reduced positively charged amine when comparing the structure of nebramine to tobramycin.^33^ Hence, our interests directed us towards the synthesis of dual-modified nebramines bearing guanidinylated amino functions and C-5 *O*-alkylation as shown in analogs 1–6 (Fig. 1) and their synergistic effects with BL–BLIs, such as ATM–AVI, FEP–TAN and MEM–XER against clinical *P. aeruginosa* isolates including MBL producing strains.

## Results and discussion

### Chemistry

#### Synthesis of amphiphilic Nebramine analogs

**Scheme 1:**
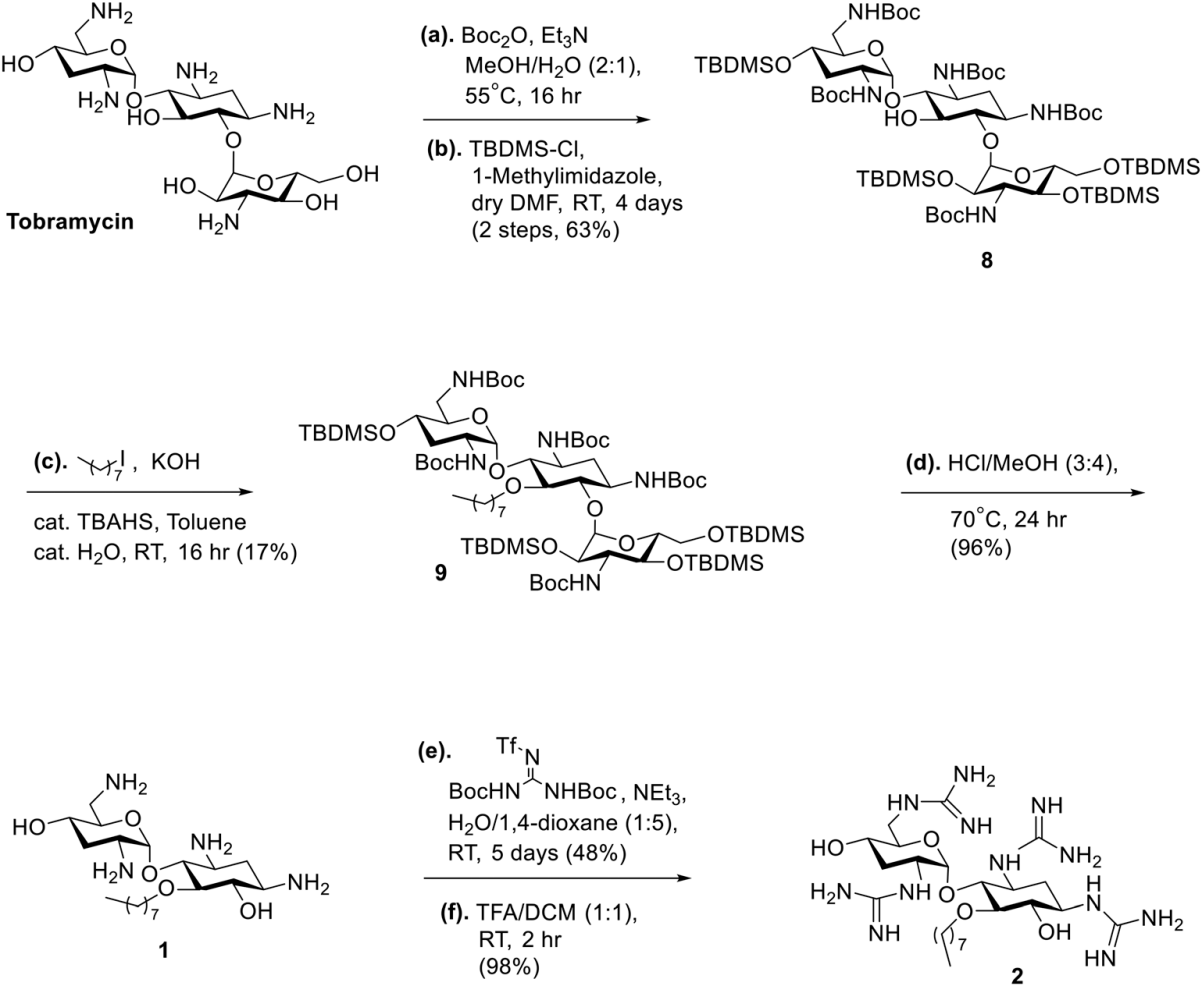
Strategy for the synthesis of amphiphilic nebramine compounds 1 and 2.

**Scheme 2:**
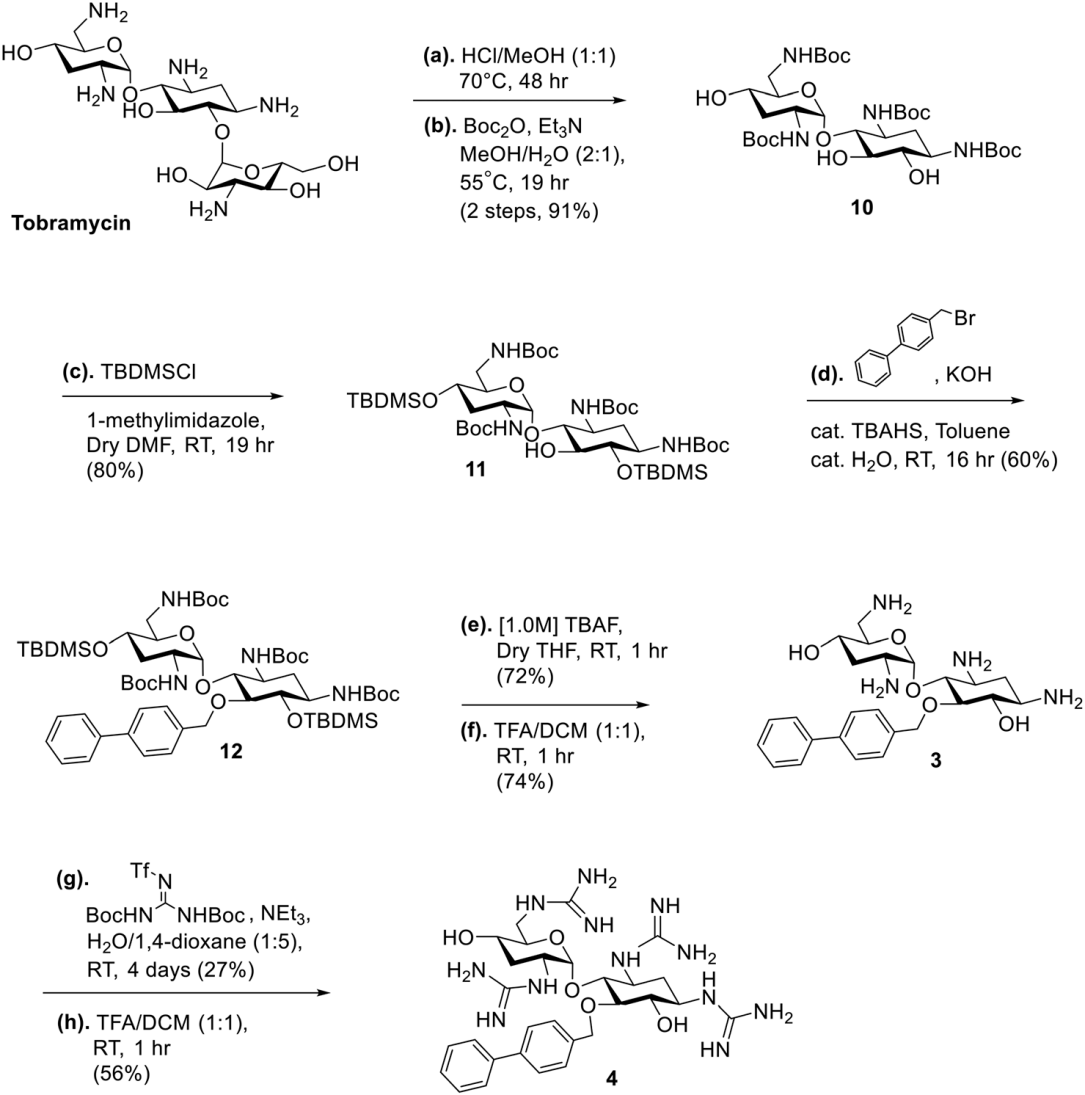
Strategy for the synthesis of amphiphilic nebramine compounds 3 and 4.

**Scheme 3:**
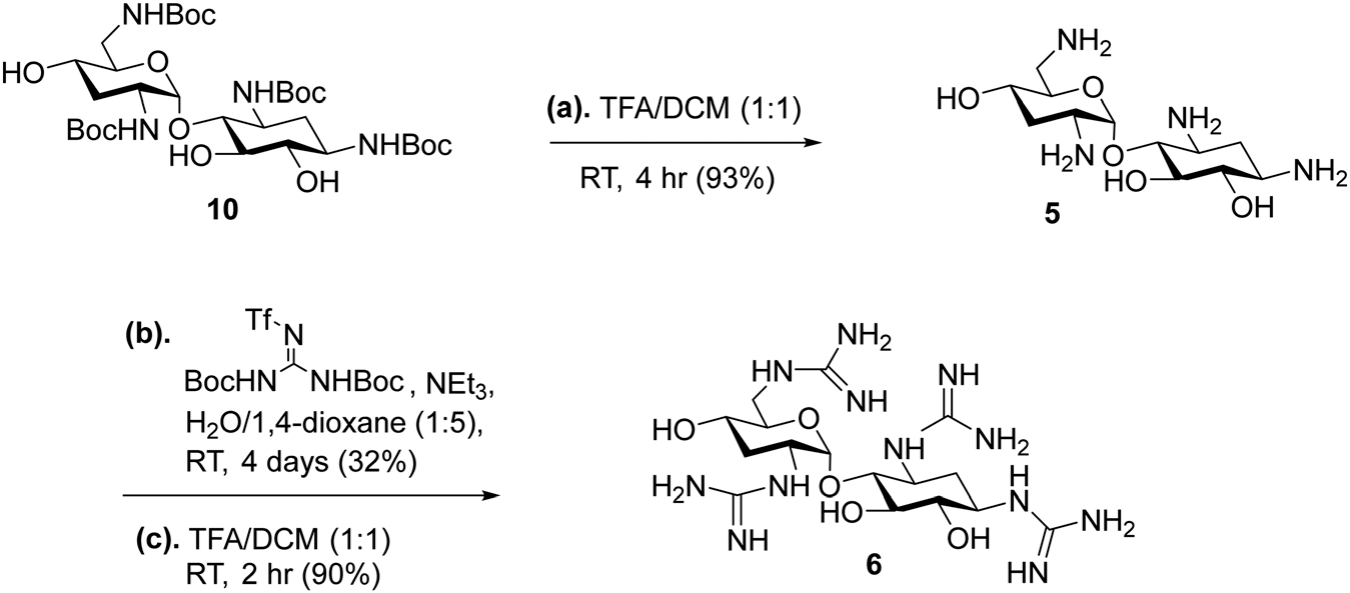
Strategy for the synthesis of compounds 5 and 6.

The strategy for the synthesis of the amphiphilic nebramine analogs (1–6) is summarized in Scheme 1–3. Alkylation of the C-5 hydroxyl was achieved by phase-transfer catalysis conditions using tetrabutylammonium sulfate as the catalyst following previous published procedures.^23,34^ For compounds 1 and 2 (Scheme 1), tobramycin was used as the starting material and the amino groups were protected using di-*tert*-butyl dicarbonate (Boc_2_O). The hydroxyl groups were protected using *tert*-butyldimethylsilyl chloride (TBDMSCl) to afford compound 8 which leaves the C-5 hydroxyl available for alkylation in the succeeding step.^23^ After the phase-transfer catalyzed alkylation producing compound 9, kanosamine was removed by exposing the tobramycin intermediate 9 to HCl and heat as previously described.^35^ Subsequently, the TBDMS- and Boc-groups from 9 were removed in the same step to produce target compound 1 following established procedures.^30,33^ The free amino groups were then subjected to guanidinylation conditions using *N*,*N′*-di-Boc-*N′′*-triflylguanidine as the guanidine source in modest yield (48%).^36^ Boc-protecting groups were then removed by trifluoroacetic acid (TFA) to yield target compound 2 (Scheme 1). The synthesis of compounds 3 and 4 (Scheme 2) introduced a challenge since the methylene–biphenyl ether moiety is acid-labile and could not survive 12 M HCl nor 1.5 M H_2_SO_4_ treatment required to hydrolyze the ether linkage between 2-deoxystreptamine and kanosamine in tobramycin.^27,31^ Therefore, a novel strategy was implemented wherein the sugar cleavage of kanosamine was performed first, followed by Boc-protection of the amines to generate 10 and selective silyl protection of the 6 and 4′ hydroxyl groups to produce 11 in 80% yield. Alkylation of 11 using 4-(bromomethyl)biphenyl afforded compound 12, which was deprotected using standard conditions to produce target compound 3. Guanidinylation of 3, then TFA treatment afforded compound 4 (Scheme 2) in the same manner as compound 2. Similarly, control compounds 5 and 6 were produced from compound 10 using the same strategy (Scheme 3).

#### Standalone activity of nebramine analogs against *P. aeruginosa* clinical isolates

The minimum inhibitory concentration (MIC) is a metric used to describe *in vitro* antibacterial activity. The MIC is defined as the lowest concentration of antibiotic to inhibit visible growth of bacteria.^37^ MICs are often used to determine if a bacterium is susceptible or resistant to an antibiotic by comparing the antibiotic's MIC to susceptibility breakpoints set by national standards and guideline organizations, such as the Clinical and Laboratory Standards Institute (CLSI).^38^ The CLSI susceptibility breakpoints of β-lactam antibiotics comprising this study are included in the ESI† in Table S1. Apart from the wild-type strain (PAO1), all strains displayed high-level resistance to β-lactam antibiotics and were designated as CRPA (MIC_MEM_ > 2 μg mL^−1^). These clinical isolates were also designated as MDR.^39^ A more detailed antibiotic susceptibility profile of all *P. aeruginosa* strains is also available in the ESI† (Table S2). All modified nebramine analogs 1–4 and 6 when used alone were inactive *versus* the strains tested (MIC > 128 μg mL^−1^), which is consistent with previous findings that indicated alkylated aminoglycosides lose ribosomal binding abilities due to loss of hydrogen bonding and steric hindrance.^25,31^ Similarly, nebramine 5 (MIC_5_ = 32 μg mL^−1^) does not possess potent standalone activity against PAO1. The 32-fold increase in MIC relative to tobramycin (MIC_TOB_ = 1 μg mL^−1^) suggests that nebramine loses significant ribosomal binding capabilities upon removal of the kanosamine sugar. This observation demonstrates that the kanosamine segment of tobramycin is important for ribosomal binding in PAO1, likely through the additional hydrogen bonding interactions the segment can participate in with bacterial 16S rRNA.^40^ While all CRPA were susceptible to colistin (MIC_CST_ ≤ 2 μg mL^−1^), with the exception of PA101243 and PA114228 (Table S2†), colistin is unfortunately considered a suboptimal treatment in most clinics due to nephro- and neurotoxicity.^41^

#### Synergy of nebramine analogs with β-lactam antibiotics against wild-type *P. aeruginosa*

*P. aeruginosa* utilize a myriad of resistance mechanisms against β-lactam antibiotics, including multidrug efflux pumps and porin reduction to reduce uptake.^17,21,22^ It is surmised that adding an outer membrane permeabilizer would lower the MIC of β-lactam antibiotics by disrupting the outer membrane. Therefore, the susceptibility of wild-type *P. aeruginosa* (PAO1) to the β-lactam antibiotics, ATM and ceftazidime (CAZ), were tested in a dual combination with sub-MIC concentrations (8 μg mL^−1^) of each of the six nebramine analogs *via* checkerboard assays (Table 1). While assessing antibiotic combinations, the fractional inhibitory concentration (FIC) index is generally accepted to represent the interactions (synergy, additive or antagonism) between antibiotic agents. An FIC index ≤ 0.5 indicates synergy between the agents. Whereas FIC indices, 0.5 > *x* ≤ 1 and 1 > *x* ≤ 4 indicates additive and antagonistic interactions, respectively.^42^ Among the derivatives, compounds 2 and 4 stood out, proving to have the highest degree of synergy with ATM and CAZ (Table 1). Moreover, it appeared that both guanidinylation and C-5 alkylation was the optimal combination of chemical modifications to efficiently potentiate β-lactam antibiotics in PAO1 when compared to compounds 1, 3 and 6 bearing single modifications. Interestingly, unmodified nebramine 5 could synergize with ATM and CAZ, but further checkerboard studies were not pursued, as nebramine 5 demonstrated moderate standalone activity (MIC_5_ = 32 μg mL^−1^) which could interfere with the interpretation of the β-lactam MICs. Based on these results, compounds 2 and 4 were chosen to pursue in further checkerboard studies against MDR *P. aeruginosa* clinical isolates.

**Table 1:** Synergy of nebramine analogs (1–6) with β-lactam antibiotics against PAO1

Analog	β-Lactam	MIC (μg mL^−1^)		Fold reduction	FIC index	Interpretation
		β-Lactam	Analog + β-lactam			
Compound 1	CAZ	4	4	1	1 < *x* < 1.06	Additive
	ATM	4	4	1	1 < *x* < 1.06	Additive
Compound 2	CAZ	4	0.5	8	0.125 < *x* < 0.188	Synergy
	ATM	4	1	4	0.25 < *x* < 0.313	Synergy
Compound 3	CAZ	4	4	1	1 < *x* < 1.06	Additive
	ATM	4	4	1	1 < *x* < 1.06	Additive
Compound 4	CAZ	4	0.5	8	0.125 < *x* < 0.188	Synergy
	ATM	8	1	8	0.125 < *x* < 0.188	Synergy
Compound 5	CAZ	4	1	4	0.5	Synergy
	ATM	4	1	4	0.5	Synergy
Compound 6	CAZ	4	2	2	0.5 < *x* < 0.56	Additive
	ATM	4	2	2	0.5 < *x* < 0.56	Additive

#### Compound 4 synergizes with β-lactam antibiotics against MDR *P. aeruginosa* clinical isolates

MDR *P. aeruginosa* are resilient and are often associated with life-threatening infections. They carry an assortment of resistance features to counter β-lactams and other classes of antibiotics. Thus, compounds 2 or 4 were tested in a dual combination with several β-lactam agents against a panel of CRPA which were also MDR (Table 2). Interestingly, 4 potentiated β-lactams such as CAZ, MEM and FEP which are typically inactive against MBL-carrying *P. aeruginosa,* by 4- to 16-fold in the MBL-carrying strains (PA86046, PA93654, PA259-96918 and PA106046). This finding demonstrates that 4 synergizes a broad panel of resistance phenotypes and MBL genotypes in *P. aeruginosa*. Importantly, all isolates except PA101243 and PA114228 remained susceptible to ATM upon addition of adjuvant 4. The additive effects of 4 with PA101243 and PA114228 are likely the result of outer membrane modifications, such as aminoarabinose or phosphoethanolamine bonded to lipid A phosphates, that confer resistance to positively charged membrane-active antibiotics such as colistin.^43,44^ Therefore, repulsive interactions could be the factor that limits interaction between the outer membrane and the cationic compound 4. The synergy of compound 2 with β-lactam antibiotics were comparable to that of the dual combinations with compound 4 (Table S3–S5†). However, 2 never outperformed 4 in any case. Therefore, compound 4 was chosen as the lead compound and tested alongside BL–BLIs against β-lactamase producing *P. aeruginosa*. Select triple combinations including compound 2 against the β-lactamase producers are available in the ESI† and were completed for comparison purposes (Tables S3–S9 and Fig. S1 and S2†).

**Table 2:** Synergy of compound 4 + β-lactam antibiotic dual combinations against MDR *P. aeruginosa*

MDR *P. aeruginosa* strain	β-Lactam	MIC (μg ml^−1^)		Fold reduction	FIC index	Interpretation
		β-Lactam	4 + β-lactam			
PA101243^a^	CAZ	512	512	1	1 < *x* < 1.06	Additive
	ATM	256	256	1	1 < *x* < 1.06	Additive
	MEM	16	8	2	0.5 < *x* < 0.56	Additive
	FEP	64	32	2	0.5 < *x* < 0.56	Additive
PA114228^a^	CAZ	8	4	2	0.5 < *x* < 0.56	Additive
	ATM	16	8	2	0.5 < *x* < 0.56	Additive
	MEM	16	8	2	0.5 < *x* < 0.56	Additive
	FEP	4	4	1	1 < *x* < 1.06	Additive
PA262-101856	CAZ	16	4	4	0.25 < *x* < 0.313	Synergy
	ATM	32	8	4	0.25 < *x* < 0.313	Synergy
	MEM	32	8	4	0.25 < *x* < 0.313	Synergy
	FEP	32	8	4	0.25 < *x* < 0.313	Synergy
PA264-104354	CAZ	64	16	4	0.25 < *x* < 0.313	Synergy
	ATM	64	8	8	0.125 < *x* < 0.188	Synergy
	MEM	64	8	8	0.125 < *x* < 0.188	Synergy
	FEP	64	8	8	0.125 < *x* < 0.188	Synergy
PA86052^b^	CAZ	256	8	32	0.031 < *x* < 0.094	Synergy
	ATM	64	1	64	0.016 < *x* < 0.078	Synergy
	MEM	16	1	16	0.063 < *x* < 0.125	Synergy
	FEP	64	8	8	0.125 < *x* < 0.188	Synergy
PA88949^b^	CAZ	128	32	4	0.25 < *x* < 0.313	Synergy
	ATM	32	8	4	0.25 < *x* < 0.313	Synergy
	MEM	32	8	4	0.25 < *x* < 0.313	Synergy
	FEP	32	16	2	0.5 < *x* < 0.56	Additive
PA107092^b^	CAZ	64	16	4	0.25 < *x* < 0.313	Synergy
	ATM	128	8	16	0.063 < *x* < 0.125	Synergy
	MEM	64	8	8	0.125 < *x* < 0.188	Synergy
	FEP	64	16	4	0.25 < *x* < 0.313	Synergy
PA108590^b^	CAZ	64	2	32	0.031 < *x* < 0.094	Synergy
	ATM	64	2	32	0.031 < *x* < 0.094	Synergy
	MEM	16	1	16	0.063 < *x* < 0.125	Synergy
	FEP	32	1	32	0.031 < *x* < 0.094	Synergy
PA109084^b^	CAZ	128	32	4	0.25 < *x* < 0.313	Synergy
	ATM	128	16	8	0.125 < *x* < 0.188	Synergy
	MEM	16	4	4	0.25 < *x* < 0.313	Synergy
	FEP	32	16	2	0.5 < *x* < 0.56	Additive
PA86056^c^	CAZ	512	32	16	0.063 < *x* < 0.125	Synergy
	ATM	16	2	8	0.125 < *x* < 0.188	Synergy
	MEM	128	16	8	0.125 < *x* < 0.188	Synergy
	FEP	128	16	8	0.125 < *x* < 0.188	Synergy
PA93654^c^	CAZ	128	32	4	0.25 < *x* < 0.313	Synergy
	ATM	16	4	4	0.25 < *x* < 0.313	Synergy
	MEM	512	128	4	0.25 < *x* < 0.313	Synergy
	FEP	128	32	4	0.25 < *x* < 0.313	Synergy
PA259-96918^c^	CAZ	512	64	8	0.125 < *x* < 0.188	Synergy
	ATM	32	8	4	0.25 < *x* < 0.313	Synergy
	MEM	1024	128	8	0.125 < *x* < 0.188	Synergy
	FEP	512	64	8	0.125 < *x* < 0.188	Synergy
PA106046^c^	CAZ	128	16	8	0.125 < *x* < 0.188	Synergy
	ATM	32	8	4	0.25 < *x* < 0.313	Synergy
	MEM	4	0.25	16	0.063 < *x* < 0.125	Synergy
	FEP	64	8	8	0.125 < *x* < 0.188	Synergy

a Colistin resistant.

b PDC producing (negative for carbapenemases: GES, KPC, NDM, IMP, VIM and OXA-48).^24^

c MBL-carriers (detected by PCR for notable carbapenemases: PA86056 [VIM-2, IMP-18], PA93654 [VIM-4], PA259-96918 [IMP-18], PA106046 [VIM-4]).^45^

#### Compound 4 synergizes with BL–BLIs against PDC- and MBL-producing *P. aeruginosa*

**Fig. 2:**
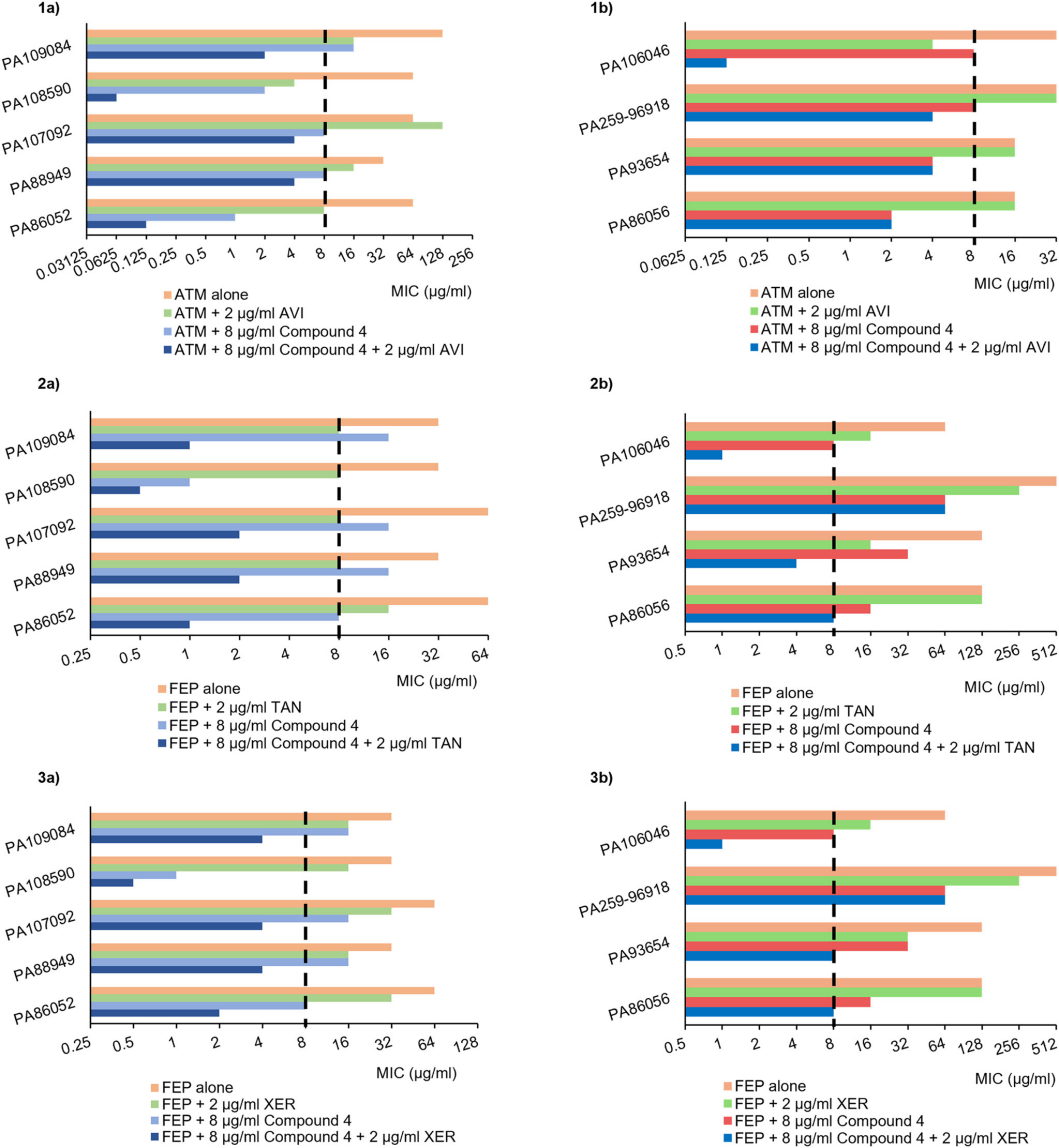
The MICs of 1) ATM alone, ATM–AVI, 4 + ATM and 4 + ATM–AVI; 2) FEP alone, FEP–TAN, 4 + FEP, 4 + FEP–TAN; 3) FEP alone, FEP–XER, 4 + FEP, 4 + FEP–XER; against the a) PDC-producing and b) MBL-carrying *P. aeruginosa*. The vertical dashed line represents the CLSI susceptibility breakpoint of the corresponding β-lactam (8 μg mL^−1^ for ATM and FEP).

Hyperproduction of PDC (ampC) is the major resistance determinant for β-lactam antibiotics with respect to *P. aeruginosa*.^46^ Similarly, MBLs pose a unique hurdle because they inactivate penicillins, cephalosporins and carbapenems. In addition to β-lactamases, other factors such as porin alterations and overexpression of efflux pumps, can render many β-lactams ineffective.^4^ Thus, compound 4 was evaluated against CRPA that produce β-lactamases, in combination with the following modern BL–BLIs: ATM–AVI, CAZ–AVI, FEP–TAN, FEP–XER, MEM–VAB (meropenem–vaborbactam) and MEM–XER. Overall compound 4 synergized with all except one BL–BLI *versus* the selected isolates resulting in 4- to 128-fold potentiation of antibiotic activity (Table 3). Similar synergistic potentiation of compound 4 with ATM–AVI, CAZ–AVI, FEP–TAN, FEP–XER, MEM–VAB and MEM–XER were seen with MBL-carrying *P. aeruginosa* (Table 4). Strikingly, the triple combination of 4 + ATM–AVI re-sensitized all PDC- and MBL-carriers to the monobactam antibiotic, ATM, and displayed coverage against the *P. aeruginosa* strains presumed to express PDCs (Fig. 2). Overall, the nine β-lactamase harbouring isolates were largely resistant to the dual combination of ATM–AVI, but all were made susceptible to ATM when exposed to the triple combination. The success of 4 + ATM–AVI in the MBL-carrying strains is likely three-fold. Firstly, MBLs are unable to hydrolyze ATM. Additionally, co-expressed β-lactamases can be inhibited by AVI.^46^ Lastly, compound 4 presumably increases the periplasmic accumulation of ATM–AVI by destabilizing the outer membrane.

**Table 3:** Synergy of compound 4 + BL–BLI triple combinations against PDC-producing *P. aeruginosa*

*P. aeruginosa* Strain	BL/BLI	MIC (μg ml^−1^)		Fold reduction	FIC index	Interpretation
		BL/BLI	4 + BL/BLI			
PA86052	CAZ–AVI	32	1	32	0.031 < *x* < 0.094	Synergy
	FEP–TAN	16	1	16	0.063 < *x* < 0.125	Synergy
	FEP–XER	32	2	16	0.063 < *x* < 0.125	Synergy
	MEM–VAB	16	1	16	0.063 < *x* < 0.125	Synergy
	MEM–XER	16	0.25	64	0.016 < *x* < 0.078	Synergy
	ATM–AVI	8	0.125	64	0.016 < *x* < 0.078	Synergy
PA88949	CAZ–AVI	8	4	2	0.5 < *x* < 0.56	Additive
	FEP–TAN	8	2	4	0.25 < *x* < 0.313	Synergy
	FEP–XER	16	4	4	0.25 < *x* < 0.313	Synergy
	MEM–VAB	32	8	4	0.25 < *x* < 0.313	Synergy
	MEM–XER	16	4	4	0.25 < *x* < 0.313	Synergy
	ATM–AVI	16	4	4	0.25 < *x* < 0.313	Synergy
PA107092	CAZ–AVI	64	4	16	0.063 < *x* < 0.125	Synergy
	FEP–TAN	8	2	4	0.25 < *x* < 0.313	Synergy
	FEP–XER	32	4	8	0.125 < *x* < 0.188	Synergy
	MEM–VAB	64	8	8	0.125 < *x* < 0.188	Synergy
	MEM–XER	64	8	8	0.125 < *x* < 0.188	Synergy
	ATM–AVI	128	4	32	0.031 < *x* < 0.094	Synergy
PA108590	CAZ–AVI	4	0.125	32	0.031 < *x* < 0.094	Synergy
	FEP–TAN	8	0.5	16	0.063 < *x* < 0.125	Synergy
	FEP–XER	16	0.5	32	0.031 < *x* < 0.094	Synergy
	MEM–VAB	8	0.5	16	0.063 < *x* < 0.125	Synergy
	MEM–XER	8	0.0625	128	0.008 < *x* < 0.07	Synergy
	ATM–AVI	4	0.0625	64	0.016 < *x* < 0.078	Synergy
PA109084	CAZ–AVI	8	2	4	0.25 < *x* < 0.313	Synergy
	FEP–TAN	8	1	8	0.125 < *x* < 0.188	Synergy
	FEP–XER	16	4	4	0.25 < *x* < 0.313	Synergy
	MEM–VAB	16	4	4	0.25 < *x* < 0.313	Synergy
	MEM–XER	16	2	8	0.125 < *x* < 0.188	Synergy
	ATM–AVI	16	2	8	0.125 < *x* < 0.188	Synergy

**Table 4:** Synergy of compound 4 + BL–BLIs against MBL-carrying *P. aeruginosa*

*P. aeruginosa* Strain	BL/BLI	MIC (μg ml^−1^)		Fold reduction	FIC index	Interpretation
		BL/BLI	4 + BL/BLI			
PA86056	CAZ–AVI	512	32	16	0.063 < *x* < 0.125	Synergy
	FEP–TAN	128	8	16	0.063 < *x* < 0.125	Synergy
	FEP–XER	128	8	16	0.063 < *x* < 0.125	Synergy
	MEM–VAB	128	8	16	0.063 < *x* < 0.125	Synergy
	MEM–XER	64	4	16	0.063 < *x* < 0.125	Synergy
	ATM–AVI	16	2	8	0.125 < *x* < 0.188	Synergy
PA93654	CAZ–AVI	128	32	4	0.25 < *x* < 0.313	Synergy
	FEP–TAN	16	4	4	0.25 < *x* < 0.313	Synergy
	FEP–XER	32	8	4	0.25 < *x* < 0.313	Synergy
	MEM–VAB	512	128	4	0.25 < *x* < 0.313	Synergy
	MEM–XER	256	32	8	0.125 < *x* < 0.188	Synergy
	ATM–AVI	16	4	4	0.25 < *x* < 0.313	Synergy
PA259-96918	CAZ–AVI	512	64	8	0.125 < *x* < 0.188	Synergy
	FEP–TAN	256	64	4	0.25 < *x* < 0.313	Synergy
	FEP–XER	256	64	4	0.25 < *x* < 0.313	Synergy
	MEM–VAB	1024	128	8	0.125 < *x* < 0.188	Synergy
	MEM–XER	1024	128	8	0.125 < *x* < 0.188	Synergy
	ATM–AVI	32	4	8	0.125 < *x* < 0.188	Synergy
PA106046	CAZ–AVI	16	0.5	32	0.031 < *x* < 0.094	Synergy
	FEP–TAN	16	1	16	0.063 < *x* < 0.125	Synergy
	FEP–XER	16	1	16	0.063 < *x* < 0.125	Synergy
	MEM–VAB	4	0.125	32	0.031 < *x* < 0.094	Synergy
	MEM–XER	2	0.03125	64	0.016 < *x* < 0.078	Synergy
	ATM–AVI	4	0.125	32	0.031 < *x* < 0.094	Synergy

#### Time-kill kinetics in PA107092

**Fig. 3:**
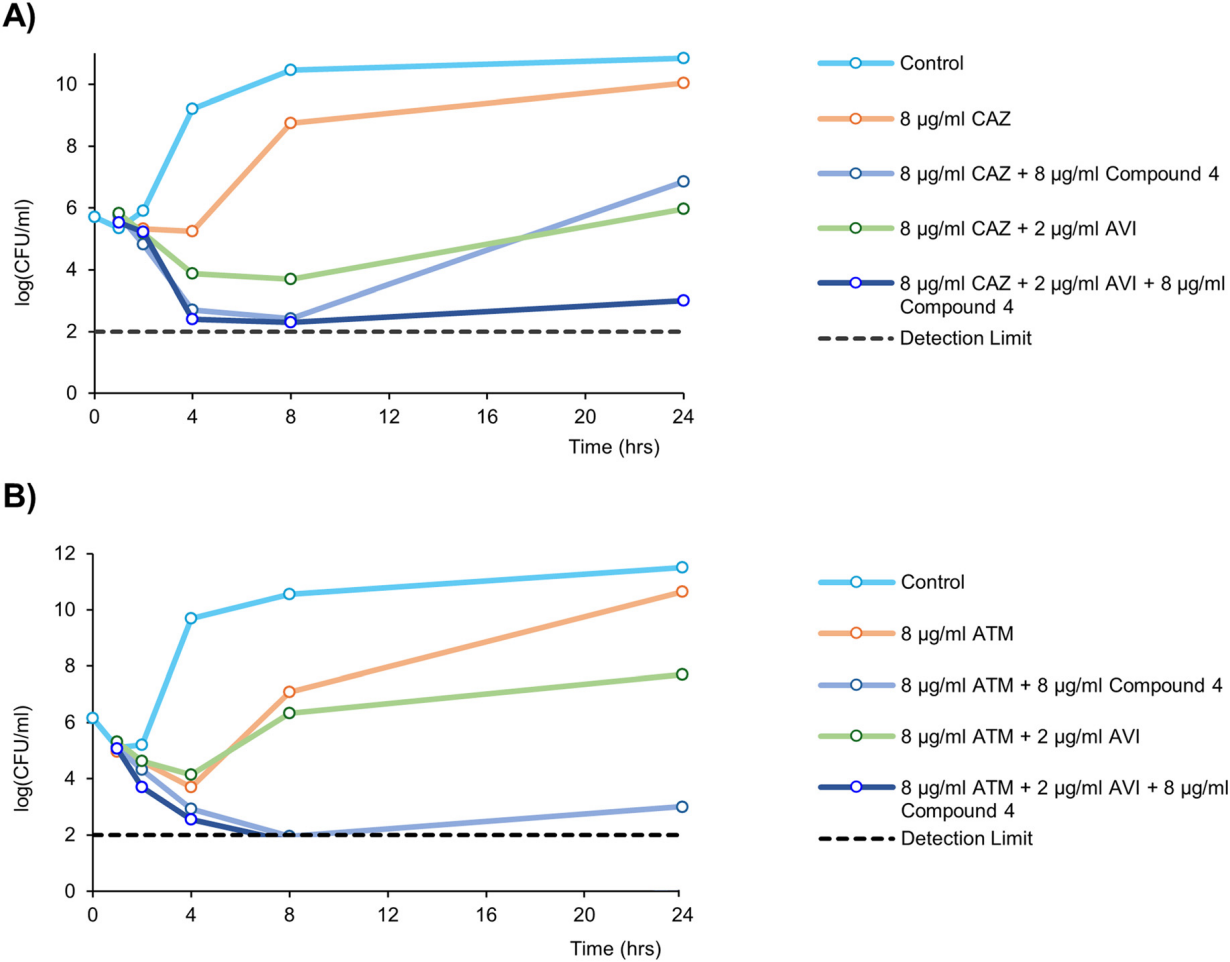
Time-kill kinetics of **A**) CAZ monotherapy, 4 + CAZ, CAZ–AVI, and triple combination 4 + CAZ–AVI; **B**) ATM alone, 4 + ATM, ATM–AVI and 4 + ATM–AVI against PDC-producing strain PA107092. Detection limit is *y* = 2 log_10_.

The time-dependent killing of select triple combinations in the PDC-producing strain PA107092 was assessed according to previously published methods.^47^ Bactericidal activity is defined as a ≥3 log_10_ decrease in CFU mL^−1^ compared to the initial time point inoculum, which is equivalent to 99.9% cell killing after 24-hours.^48^ Whereas bacteriostatic activity is a <3 log_10_ decrease from the initial inoculum after 24-hours.^49^ For the time-kill kinetics studies, a concentration of 8 μg mL^−1^ for both CAZ and ATM was selected, as this concentration in combination with 8 μg mL^−1^ of compound 4 and 2 μg mL^−1^ AVI inhibited visible PA107092 growth in checkerboard assays (Table 4). Under CAZ monotherapy, growth at the 24-hour timepoint was similar to the positive control (Fig. 3A) which is consistent with a previous report on this strain.^27^ In both the dual combinations, nearly full bacterial regrowth at 24-hours was observed compared to the initial inoculum. The 4 + CAZ dual combination resulted in a sharp decrease in growth (≥3 log_10_) at 4 hours, but resurgence of the bacterial population occurred within 24-hours. Incorporation of all three agents 4 + CAZ–AVI caused a similar ≥3 log_10_ fold reduction in bacterial population at 4 hours which persisted up to 8 hours. However, slight regrowth was observed at 24-hours with total colony counts reduced compared to the dual combinations.

ATM monotherapy time-kill kinetics behaved nearly identical to CAZ monotherapy against PA107092. A slight reduction in colony number was observed at the 1- and 2-hour timepoints, and regrowth of the bacterial population occurred beyond 4 hours (Fig. 3B). The ATM–AVI results only differed from ATM monotherapy by which the sharp bacterial regrowth occurred after 8 hours instead of 4 hours. In contrast to ATM monotherapy and ATM–AVI, both 4 + ATM and 4 + ATM–AVI exhibited a ≥3 log_10_ reduction in CFU mL^−1^ at the 4 hour timepoint. However, re-growth of the bacterial population occurred with 4 + ATM dual combination after 24-hours, whereas the triple combination of 4 + ATM–AVI was bactericidal after 4-hours. Interestingly, 4 + ATM resulted in a much lower bacterial count than 4 + CAZ against PA107092 after 24-hours. Overall, these data highlight that the 4 + ATM dual combination is much more active than the ATM–AVI dual combination beyond 4 hours against PA107092. Also, 4 + ATM–AVI is bactericidal, while 4 + CAZ–AVI does not exhibit bactericidal action in the time range tested against PA107092.

#### Mode-of-action studies

Compound 4 is proposed to act as an outer membrane permeabilizer. It is believed that positively charged amphiphilic molecules like compound 4, interact with negatively charged phosphates present in the lipid A of lipopolysaccharide. This interaction destabilizes bridging Mg^2+^ and Ca^2+^ ions essential for outer membrane integrity in a mechanism coined “self-promoted uptake”.^50,51^ Compound 7 is analogous to compound 4 and was demonstrated to induce outer membrane permeabilization based on fluorescent permeability assays.^27^ Therefore, it is reasonable to assume compound 4 acts in a similar fashion. Moreover, if a Gram-negative impermeable antibiotic such as rifampicin (RIF) can be potentiated, it is plausible that compound 4 acts as an outer membrane permeabilizer. Evidently, 4 potentiates RIF by 16-fold against PAO1 according to checkerboard assays. In addition, the potentiation effect is diminished upon elevating Mg^2+^ levels (20 mM [Mg^2+^]) in the bacterial medium (Table 5). Supplementing Mg^2+^ promotes outer membrane integrity which assumably limits the interaction of compound 4 with lipid A phosphates.

**Table 5:** RIF potentiation by compound 4 in normal and 20 mM [Mg^2+^] cation-adjusted Mueller–Hinton broth (CAMHB)

Conditions	Antibiotic	MIC (μg mL^−1^)	MIC (μg mL^−1^)	Fold reduction	FIC index
		Antibiotic	4 + antibiotic		
CAMHB	RIF	32	2	16	0.063 < *x* < 0.125
20 mM [Mg^2+^] CAMHB	RIF	>32	>32	ND	ND

#### Nephrotoxicity assessment

**Fig. 4:**
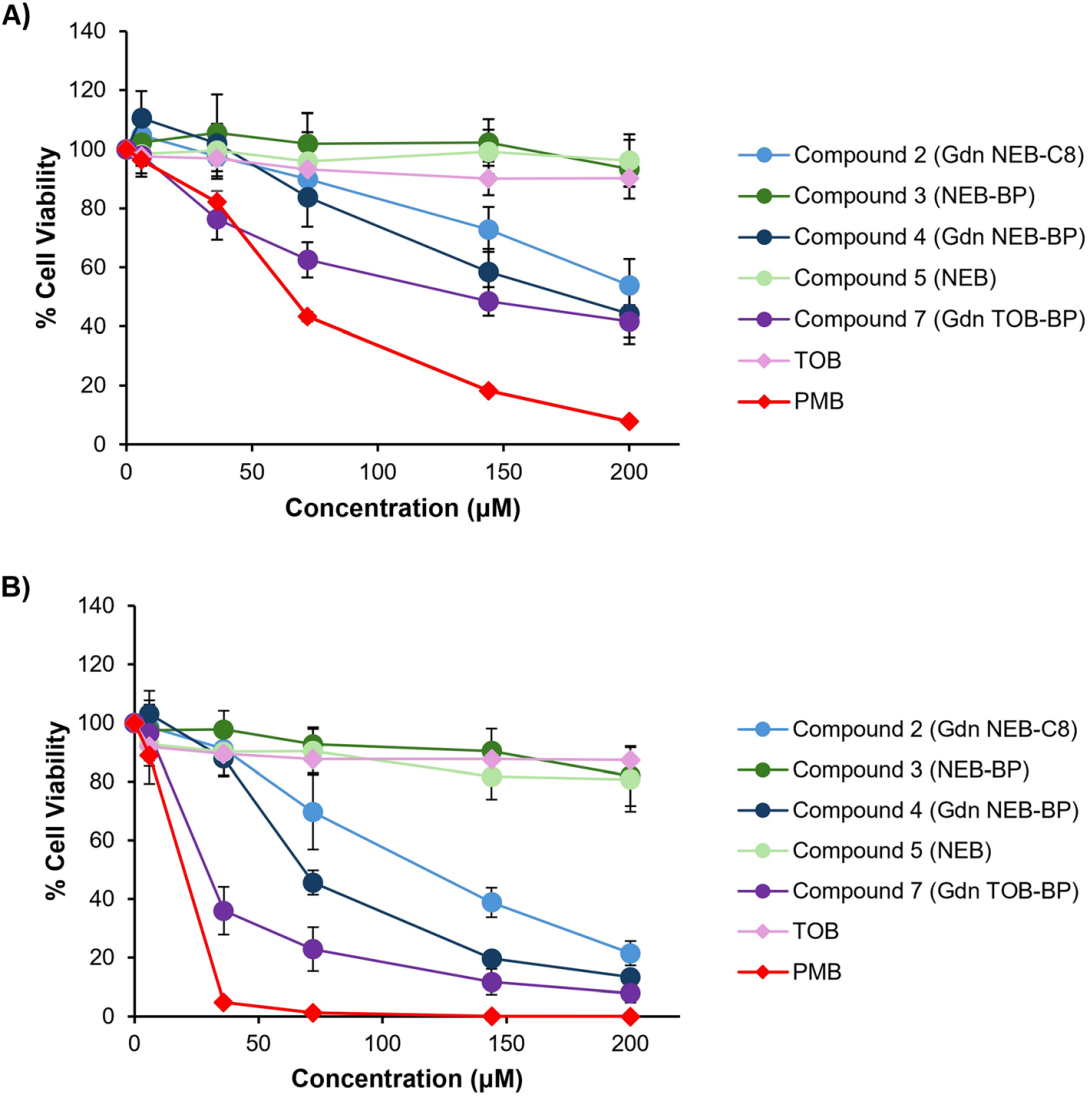
Cell viability of proximal renal tubule cell lines A) RPTEC and B) HK-2 suspended in various concentrations (0 μM, 6 μM, 36 μM, 72 μM, 144 μM and 200 μM) of chemical agents. TOB = tobramycin, PMB = polymyxin B.

Nephrotoxicity is a major limitation in the use of cationic antibiotics, such as polymyxin B for treatment of bacterial infections by clinicians.^29^ Hence, polymyxin B is an appropriate positive control to use as a benchmark for the following preliminary nephrotoxicity experiments. The cell lines selected are the immortal proximal renal tubule epithelial cells, RPTEC and HK-2, from the adult human kidney.^52,53^ The rationale behind reducing the number of positively charged groups from tobramycin is that previous evidence suggested a minimized scaffold (compound 4) may offer reduced cytotoxic properties.^30,32^ For that reason, the cell viability of RPTEC and HK-2 upon treatment of tobramycin, polymyxin B, compounds 2–5 and compound 7 (at concentrations of 0 μM, 6 μM, 36 μM, 72 μM, 144 μM and 200 μM) were assessed (Fig. 4).

Polymyxin B was the most cytotoxic agent to both cell lines at each concentration. In stark contrast, >90% of RPTEC cells and >80% of HK-2 cells incubated with 36 μM of lead compound 4, a concentration which is approximately three times greater (23 μg mL^−1^) than the active concentration (8 μg mL^−1^) used for the checkerboard and time-kill assays conducted herein, were viable. As expected, compound 7 was more toxic than compound 4; thus, at 36 μM, about 40% of HK-2 cells and 70% of RPTEC cells were viable. The enhanced toxicity likely arises from the extra guanidinium group in 7. The biphenyl group (compound 4) caused a greater loss of cell viability than the octyl function (compound 2) in these cells. The non-guanidinylated compound 3 was less toxic than compound 4, an indication that modification of the amino groups into guanidinium groups increases toxicity. Nebramine 5 and tobramycin with no chemical alterations, had no effect on the viability of the cells at all concentrations investigated. Overall, it appears that introduction of the biphenyl function and increasing the number of guanidinium groups increased the cytotoxicity of compounds against RPTEK and HK-2 cells.

#### Comparison of compound 4 to previously reported compound 7

Compound 4 is a minimized analog based on the previously reported tobramycin derivative 7, which contains an additional guanidinylated aminosugar, kanosamine, linked at the C-6 position of nebramine. As a consequence, tobramycin bears an extra positive charge in comparison to nebramine and is expected to have superior membrane permeabilizing capabilities. Compound 7 was previously found to synergize with CAZ–AVI and ATM–AVI combinations against the PDC-producers in this report.^27^ Retention of this synergy by compound 4 was observed (Table 3) in direct comparison to compound 7. The triple combinations with CAZ–AVI or ATM–AVI differed in potentiation by 4-fold at most (Fig. S3 and S4†), suggesting that the guanidinylated kanosamine sugar attributes to a minor part in the outer membrane permeabilization effect of compound 7, and that 4 is an effective alternative that is also less damaging to kidney cells.

## Conclusions

β-Lactam antibiotics are the most widely prescribed agents for treating bacterial infections.^54^ It is paramount that the scientific community prioritizes enhancing agents to prolong β-lactam antibiotic activity. One method to preserve β-lactam susceptibility in *P. aeruginosa*, is to increase their permeability across the outer membrane, such that they reach their periplasmic target, the PBP. Inspired by previous research on dual modified tobramycin-based outer membrane permeabilizers, novel dual modified nebramine analogs were prepared. Lead compound 4 was then explored in combination with β-lactam antibiotics and BL–BLIs against *P. aeruginosa* clinical isolates including MBL-producing strains. Although 4 is inactive as a standalone agent (MIC_4_ > 128 μg mL^−1^), it can potentiate a variety of β-lactam antibiotics and BL–BLIs against several MDR, PDC- and MBL-carrying *P. aeruginosa.* Importantly, adding 4 with ATM–AVI as a triple combination restored susceptibility to ATM in all PDC- and MBL-carrying *P. aeruginosa* tested. Time-kill kinetics revealed this triple combination to have bactericidal activity on the PDC-producer PA107092 after 4-hours. Compound 4 demonstrated comparable potentiating effects to previously reported compound 7, while simultaneously displaying markedly reduced cytotoxic properties. Overall, there is an unfulfilled urgent need for non-nephrotoxic agents that increase antibiotic permeability in *P. aeruginosa*. Compound 4 fulfils this need *in vitro* and is effective as a third component with several state-of-the-art BL–BLIs like FEP–TAN, FEP–XER, MEM–XER and ATM–AVI combinations against *P. aeruginosa* clinical isolates.

## Experimental

### Chemistry

Reagents were obtained commercially from Sigma Alrich, Biosynth, TCI America or AK Scientific and used without further purification. Chemical reactions were monitored using thin-layer chromatography (TLC) on 0.25 mm silica gel 60 F254 plates from Merck and visualized under a UV lamp (*λ* = 254 nm), staining by 20% H_2_SO_4_ in ethanol or ninhydrin staining. Intermediates and final compounds were purified using normal or reverse-phase flash chromatography with SiliaFlash P60 (40–63 μm) 60 Å silica gel or SiliaBond C18 (40–63 μm) 60 Å silica gel from SiliCycle, respectively, or using the Biotage Selekt Flash instrument with Biotage Sfär columns. Afterwards, characterization of the chemical structure and molecular connectivity was achieved by nuclear magnetic resonance (^1^H NMR, ^13^C NMR, and 2D NMR experiments) spectroscopy on a Bruker AMX-400 MHz or 500 MHz spectrometer (Fig. S5–S56†). The mass of each compound and intermediate was determined by matrix-assisted laser desorption ionization mass spectrometry (MALDI-MS) on a Bruker Daltonics ultraflex MALDI-time-of-flight (TOF) mass spectrometer or by electrospray ionization (ESI) on a Varian 500-MS ion trap mass spectrometer.

#### General procedure A for C5-O-alkylation of nebramine derivatives

TBDMS- and Boc-protected compound (1 eq.) was placed with potassium hydroxide (KOH) (3 eq.) and tetrabutylammonium hydrogen sulfate (TBAHS) (0.4 eq.) in a round bottom flask and dissolved in toluene and catalytic amount of H_2_O. After 10 minutes of stirring, alkyl halide (3 eq.) was added. The reaction was stirred overnight at ambient temperature and monitored *via* TLC. The reaction continued for 16 hours before TLC indicated significant conversion of the product (rf = 0.56, 20% EtOAc/hexanes). The mixture was quenched with ethyl acetate (EtOAc) and H_2_O. The aqueous layer was washed with EtOAc (3×). The combined organic layers were dried over anhydrous sodium sulfate (Na_2_SO_4_) and concentrated by rotary evaporation to recover a white powder.

#### General procedure B for deprotection of Boc protecting groups

*N*-Boc-protected compounds were dissolved in 2 ml dichloromethane (DCM) and exposed to dropwise addition of 2 ml trifluoroacetic acid (TFA). The reaction was carefully monitored by TLC and only continued for maximum 1 hour for the biphenyl derivatives and 2–3 hours for derivatives lacking a TFA-labile moiety. Next, the solution was concentrated by rotary evaporation to afford an off-white solid. The residue was washed in 2% methanol (MeOH)/diethyl ether before further purification. Purification was achieved by reverse-phase flash chromatography and compounds eluted in 0–20% MeOH/de-ionized H_2_O (v/v).

#### General procedure C for guanidinylation of nebramine analogs

Nebramine analogs (1 eq.) with free amines were dissolved in a round bottom flask with 0.5 ml H_2_O and stirred. 1/5th of total 15 eq. *N*,*N′*-di-Boc-*N*′′-triflylguanidine was added directly, followed by 0.5 ml 1,4-dioxane and solution was stirred until clear. Addition of *N*,*N′*-di-Boc-*N′′*-triflylguanidine and subsequent 1,4-dioxane addition was repeated four times until all *N*,*N′*-di-Boc-*N′′*-triflylguanidine (15 eq. total) and 1,4-dioxane (2.5 mL total) was added. After 5 min, NEt_3_ (15 eq.) was added, and the reaction was allowed to stir for 3–5 days until TLC showed conversion of the product. The solvent was removed *via* rotary evaporation by slowly reducing pressure. The material was then dissolved in DCM, transferred to a separatory funnel, and washed with H_2_O (3×). The organic layer was then dried over Na_2_SO_4_, and concentrated to a resin *in vacuo*. The crude residue was purified by normal-phase flash chromatography (P60 silica gel) using MeOH/DCM as the eluent gradient. The pure compound eluted in 5% MeOH/DCM in each case and was further characterized by NMR and MS experiments.

5-O-(Octyl)-1,3,2′,6′,3′′-penta-*N*-Boc-4′,2′′,4′′,6′′-tetra-*O*-TBDMS-tobramycin (9) and compound 7 were synthesized according to previous publication, matching spectral characteristics.^27^

#### Synthesis of 5-O-(octyl)-nebramine (1)

Intermediate 9 (81 mg, 1 eq.) was dissolved in MeOH, followed by dropwise addition of HCl (HCl : MeOH [3 : 4]). The reaction mixture was refluxed for 24-hours. Compound 1 (21 mg, 96%) was directly concentrated under reduced pressure and purified by reverse-phase flash chromatography (eluted in 100% de-ionized water). ^1^H NMR (500 MHz, D_2_O): *δ* 5.57 (d, *J* = 2.8 Hz, 1H, anomeric H-1′), 4.00 (m, 3H), 3.83 (m, 1H), 3.80–3.66 (m, 2H), 3.61 (m, 1H), 3.52 (m, 1H), 3.46–3.26 (m, 4H), 2.48 (dt, *J* = 12.6, 4.3 Hz, 1H), 2.30 (dt, *J* = 13.6, 4.2 Hz, 1H), 2.12 (dt, *J* = 13.4, 8.6 Hz, 1H), 1.84 (q, *J* = 12.6 Hz, 1H), 1.62 (m, 2H), 1.30 (m, 10H, octyl-methylene), 0.91–0.82 (t, 3H, octyl-CH_3_). ^13^CNMR (126 MHz, D_2_O): *δ* 92.09 (anomeric C-1′), 82.78, 75.41, 73.42, 72.92, 72.81, 63.85, 49.90, 48.90, 47.54, 39.31, 31.13, 29.48, 28.94, 28.65, 28.48, 28.28, 25.27, 22.07, 13.45. ESI-MS: *m/z* calculated for C_20_H_43_N_4_O_5_^+^ = 419.3233 [M + H]^+^; found = 419.3251 [M + H]^+^.

#### Synthesis of 5-O-(octyl)-guanidino-nebramine (2)

Compound 1 (21 mg) was subjected to the conditions outlined in general procedure C and recovered as a pure white solid (33.5 mg, 48%). The product from the previous step (30 mg) was exposed to the conditions of general procedure B and yielded crystalline solid 2 (12.6 mg, 99%). ^1^H NMR (500 MHz, D_2_O) *δ* 5.53 (d, 1H, anomeric H-1′), 4.04–3.97 (m, 1H), 3.88–3.82 (m, 1H), 3.75–3.63 (m, 3H), 3.62–3.43 (m, 7H), 2.31–2.19 (m, 2H), 1.79–1.50 (m, 4H), 1.29 (s, 10H), 0.98–0.77 (m, 3H). ^13^C NMR (126 MHz, D_2_O) *δ* 157.97 (guanidine), 157.21 (guanidine), 156.62 (guanidine), 155.94 (guanidine), 95.00 (anomeric C-1′), 84.61, 77.13, 75.44, 73.96, 72.00, 63.86, 52.07, 50.67, 49.09, 41.82, 32.32, 32.28, 31.08, 29.39, 28.59, 28.37, 25.24, 22.07, 13.45. ESI-MS: *m/z* calculated for C_24_H_52_N_12_O_5_^2+^ = 294.2087 [M + 2H]^2+^; found = 294.2086 [M + 2H]^2+^.

#### Synthesis of 1,3,2′,6′-tetra-*N*-Boc-nebramine (10)

A round bottom flask containing 2.1 g tobramycin sulfate (1 eq.) was charged with a magnetic stir bar. The white powder was completely solubilized within 1 hour by adding 25 ml MeOH, then 25 ml 12 M HCl dropwise. After acid addition, the reaction was allowed to reflux for 48 hours. The reaction mixture was directly concentrated *in vacuo* and a brown resin was neutralized to pH = 7 by dropwise addition of NH_4_^+^ OH^−^ solution. The material was concentrated again and used without further purification for the next step. The reaction mixture from the previous step was dissolved in MeOH/H_2_O (2 : 1). Further, triethylamine (NEt_3_) (16 eq.) and Boc-anhydride (8 eq.) were added, then the solution was refluxed overnight until TLC indicated complete conversion of the product (Rf = 0.5, solvent system: DCM/MeOH [15 : 1]). The solution was concentrated under reduced pressure to yield a brown solid. The crude mixture was purified by normal-phase flash chromatography and pure compound 10 was eluted in 4% MeOH/DCM (v/v) to afford a white powder (2.9 g, 91% yield). ^1^H NMR (500 MHz, MeOD) *δ* 6.62–6.52 (s, 1H), 5.10 (d, 1H, anomeric), 3.75–3.53 (m, 2H), 3.52–3.32 (m, 6H), 3.15 (m, 1H), 2.15–1.88 (m, 2H), 1.64 (m, 1H), 1.44 (m, 36H), 1.32 (m, 1H). ^13^C NMR (126 MHz, MeOD) *δ* 159.51, 159.29, 158.19, 157.74, 99.17, 82.40, 80.66, 80.36, 80.13, 79.84, 78.75, 76.58, 73.43, 66.51, 52.30, 51.16, 51.02, 41.99, 36.10, 34.17, 28.88, 28.76, 28.74, 28.66. MALDI-TOF-MS: *m/z* calculated for C_32_H_58_N_4_O_13_Na^+^ = 729.3898 [M + Na]^+^; found = 729.3771 [M + Na]^+^.

#### Synthesis of 1,3,2′,6′-tetra-*N*-Boc-6,4′-bis-*O*-TBDMS-nebramine (11)

1.295 g of intermediate 10 (1 eq.) was crushed by mortar and pestol into a fine powder and placed into a dry 50 ml round bottom flask with TBDMSCl (6 eq.). The solids were dissolved in anhydrous DMF then air expelled *via* N_2_ inlet and air outlet. 1-Methylimidazole (12 eq.) was added dropwise over N_2_-atmosphere and stirred for 20 hours. The residue was evaporated under reduced pressure then dissolved in 200 ml EtOAc and washed with ice-cold H_2_O (×3) and once with brine solution. The organic layer was dried over anhydrous Na_2_SO_4_, filtered, and concentrated *via* rotary evaporation to reveal an off-white solid. The crude was purified by flash column chromatography and the pure compound eluted in 2% MeOH/DCM (v/v) to yield compound 11 (1.38 g, 80%) as a crystalline solid. ^1^H NMR (500 MHz, CDCl_3_) *δ* 5.59 (s, 1H), 5.17–4.74 (m, 3H, anomeric), 4.29 (s, 1H), 3.73 (s, 1H), 3.69–3.06 (m, 10H), 2.60 (s, 1H), 2.33 (s, 1H), 2.01 (m, 1H), 1.66–1.54 (m, 1H), 1.42 (m, 36H, Boc), 0.87 (m, 18H, TBDMS *t*-Bu), 0.13–0.02 (m, 12H, TBDMS methyl). ^13^C NMR (126 MHz, CDCl_3_) *δ* 155.62, 155.29, 155.28, 155.08, 99.07 (C1′), 82.24, 80.31, 79.77, 79.44, 79.19, 77.85, 72.68, 67.19, 51.37, 50.49, 49.19, 41.30, 35.13, 34.64, 28.57, 28.56, 28.52, 28.38, 26.02, 25.88, 18.33, 17.99, −4.01, −4.17, −4.84. MALDI-TOF-MS: *m/z* calculated for C_44_H_86_N_4_O_13_Si_2_Na^+^ = 957.5628 [M + Na]^+^; found = 957.5310 [M + Na]^+^.

#### Synthesis of 5-O-(methylenebiphenyl)-1,3,2′,6′-tetra-*N*-Boc-6,4′-bis-*O*-TBDMS-nebramine (12)

Compound 11 (660 mg, 1 eq.) was subjected to conditions outlined in general procedure A with 4-(bromomethyl)biphenyl as the alkyl halide (3 eq.). The product was eluted in 16% EtOAc/hexanes (v/v) by normal-phase flash chromatography and recovered as a white powder (464 mg, 60%). ^1^H NMR (500 MHz, CDCl_3_) *δ* 7.57 (m, 2H, biphenyl), 7.51 (m, 2H, biphenyl), 7.45–7.38 (m, 2H, biphenyl), 7.30 (m, 3H, biphenyl), 5.44–4.88 (m, 4H), 4.78 (m, 2H), 4.33 (s, 1H), 3.80–3.31 (m, 9H), 3.20 (s, 1H), 2.37–2.17 (m, 1H), 2.05–1.88 (m, 1H), 1.44 (m, 20H), 1.26 (s, 18H), 0.97–0.75 (m, 18H), 0.21–−0.14 (m, 12H). ^13^C NMR (126 MHz, CDCl_3_) *δ* 156.07, 155.09, 154.76, 141.08, 140.23, 137.03, 128.82, 127.28, 127.18, 127.09, 79.34, 68.06, 48.66, 35.50, 32.08, 29.85, 28.64, 28.58, 28.35, 26.09, 25.93, 18.08, 14.26, −3.71, −4.04, −4.75. ESI-MS: *m*/*z* calculated for C_57_H_97_N_4_O_13_Si_2_^+^ = 1101.6591 [M + H]^+^; found = 1101.6519 [M + H]^+^.

#### Synthesis of 5-O-(methylenebiphenyl)-nebramine (3)

A dry stir bead and compound 12 (377 mg, 1 eq.) in an oven dried round bottom flask, was purged with N_2_-atmosphere through a septum. Anhydrous THF was added, then [1.0 M] TBAF/THF was added dropwise and stirred at room temperature for 1 hour. The reaction was concentrated under reduced pressure to reveal a dark resin. The crude mixture was purified by flash chromatography and eluted in 4% MeOH/DCM (v/v) to reveal a white powder (215.2 mg, 72%). 207 mg of 5-O-(methylenebiphenyl)-1,3,2′,6′-tetra-*N*-Boc-nebramine was solubilized in DCM and then exposed to dropwise addition of TFA as per the conditions outlined in general procedure B. Pure compound 3 (83 mg, 74%) was eluted in 100% de-ionized H_2_O. ^1^H NMR (500 MHz, D_2_O) *δ* 7.80 (d, 2H, biphenyl), 7.76 (d, 2H, biphenyl), 7.58 (t, 4H, biphenyl), 7.49 (t, 1H, biphenyl), 5.35 (d, 1H, anomeric), 5.17 (d, 1H, O-CH_2_-biphenyl), 4.86–4.80 (d, 1H, O-CH_2_-biphenyl), 4.10 (m, 2H), 3.92–3.76 (m, 3H), 3.64–3.55 (m, 1H), 3.44 (m, 1H), 3.29–3.17 (m, 2H), 3.13 (m, 1H), 2.56 (m, 1H, H-2), 2.04–1.98 (m, 2H, H-3′), 1.97–1.86 (m, 1H, H-2). ^13^C NMR (126 MHz, D_2_O) *δ* 141.06, 139.85, 136.54, 129.41, 129.34, 128.16, 127.49, 126.99, 92.45, 83.23, 76.00, 75.05, 74.64, 73.03, 63.28, 49.82, 48.87, 47.05, 38.60, 28.31, 27.93. ESI-MS: *m/z* calculated for C_25_H_37_N_4_O_5_^+^ = 473.2764 [M + H]^+^; found = 473.2717 [M + H]^+^.

#### Synthesis of 5-O-(methylenebiphenyl)-guanidino-nebramine (4)

Compound 3 (47 mg, 1 eq.) was exposed to the reaction conditions highlighted in general procedure C and purified as such. The product was recovered as a pure white powder (39 mg, 27%). 5-O-(Methylenebiphenyl)-Boc_8_-guanidino-nebramine (35 mg) from the previous step was subjected to the conditions in general procedure B. The crystalline solid (8.7 mg, 56%) was purified by reverse-phase flash chromatography and eluted in 20% MeOH/de-ionized H_2_O (v/v) and characterized by NMR. ^1^H NMR (500 MHz, D_2_O) *δ* 7.73 (m, 4H, biphenyl), 7.56 (m, 2H, biphenyl), 7.48 (m, 3H, biphenyl), 5.48 (d, *J* = 3.8 Hz, 1H, anomeric H-1′), 5.21 (d, *J* = 11.5 Hz, 1H, CH_2_-biphenyl), 4.72 (d, *J* = 11.6 Hz, 1H, CH_2_-biphenyl), 3.86–3.75 (m, 2H, H-4, H-5), 3.75–3.67 (m, 2H), 3.66–3.56 (m, 3H), 3.55–3.45 (m, 3H), 2.31 (dt, *J* = 13.0, 4.3 Hz, 1H), 2.15 (dt, *J* = 12.1, 4.6 Hz, 1H), 1.77–1.61 (m, 2H). ^13^C NMR (126 MHz, D_2_O) *δ* 157.92, 157.26, 156.63, 155.63, 140.30, 140.10, 136.63, 129.28, 128.13, 127.97, 127.15, 126.92, 95.14 (C1′), 84.54 (C5), 77.51 (C4), 75.68, 74.35 (biphenyl-CH_2_-O), 71.98, 63.75, 52.17, 50.77, 48.99, 41.79, 32.32, 32.23. ESI-MS: *m*/*z* calculated for C_29_H_46_N_12_O_5_^2+^ = 321.1852 [M + 2H]^2+^; found = 321.1814 [M + 2H]^2+^.

#### Synthesis of nebramine (5)

Compound 10 (155 mg) was exposed to the conditions included in general procedure B. Nebramine (62.6 mg, 93%) was recovered in 100% de-ionized H_2_O through a reverse-phase C18 column as an off-white solid. ^1^H NMR (500 MHz, D_2_O) *δ* 5.77 (d, *J* = 3.6 Hz, 1H, anomeric H-1′), 4.03–3.84 (m, 2H, H-4, H-5′), 3.79–3.63 (m, 3H, H-4′, H-5, H-2′), 3.62–3.51 (m, 2H, H-6, H-3), 3.44 (dd, *J* = 13.8, 3.7 Hz, 1H, H-
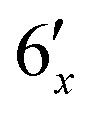
), 3.39–3.31 (m, 1H, H-1), 3.27 (dd, *J* = 13.6, 6.9 Hz, 1H, H-
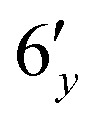
), 2.53 (m, 1H, H-2_eq_), 2.31 (m, 1H, H-
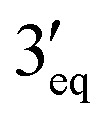
), 2.02 (m, 1H, H-
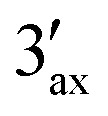
), 1.90 (m, 1H, H-2_ax_). ^13^C NMR (126 MHz, D_2_O) *δ* 94.32 (C-1′), 77.66 (C-4), 75.23 (C-5), 72.60 (C-6), 70.06 (C-5′), 64.62 (C-4′), 49.78 (C-1), 48.57 (C-3), 47.92 (C-2′), 39.98 (C-6′), 29.41 (C-3′), 28.35 (C-2). ESI-MS: *m*/*z* calculated for C_12_H_27_N_4_O_5_^+^ = 307.1981 [M + H]^+^; found = 307.1836 [M + H]^+^.

#### Synthesis of guanidino-nebramine (6)

Compound 5 (32.3 mg, 1 eq.) was exposed to conditions according to general procedure C. The pure product (39.9 mg, 32%) was eluted in 5% MeOH/DCM using a normal-phase flash column. Compound 6 was then synthesized by following general procedure B, using the Boc_8_-guanidino-nebramine (39.1 mg) intermediate from the previous step as the starting material. The pure product was purified by reverse-phase flash chromatography (13.1 mg, 90%) and eluted in 100% de-ionized H_2_O. ^1^H NMR (500 MHz, D_2_O) *δ* 5.47 (d, *J* = 3.6 Hz, 1H, anomeric H-1′), 3.84–3.75 (m, 1H), 3.74–3.60 (m, 4H), 3.60–3.48 (m, 4H), 3.48–3.40 (m, 1H), 2.29 (m, 1H), 2.26–2.18 (m, 1H), 1.78 (m, 1H), 1.69 (m, 1H). ^13^C NMR (126 MHz, D_2_O) *δ* 157.96, 157.14, 156.57, 156.11, 96.18, 80.45, 76.20, 75.22, 72.00, 63.99, 51.86, 50.64, 49.35, 41.91, 32.49, 31.80. ESI-MS: *m*/*z* calculated for C_16_H_36_N_12_O_5_^2+^ = 238.1461 [M + 2H]^2+^; found = 238.1469 [M + 2H]^2+^.

### Biological analyses

All antibacterial agents were purchased and used without further purification. Tobramycin was purchased from AKScientific. Aztreonam was purchased from Sigma-Aldrich. Meropenem and ceftazidime were purchased from TCI America. Cefepime was purchased from ThemoScientific. Taniborbactam, xeruborbactam and avibactam were purchased from MedChem express. Vaborbactam was purchased from Cayman chemicals.

#### Bacterial strains

Bacterial isolates were obtained from the American Type Culture Collection (ATCC), from the Canadian National Intensive Care Unit (CAN-ICU), or Canadian Ward (CANWARD) surveillance studies.^55,56^ Non-susceptibility was determined using CLSI guidelines and CLSI established breakpoints.^38^ If no CLSI breakpoint has yet been defined for a particular BL/BLI, the breakpoint of the β-lactam antibiotic alone was used for brevity purposes. MDR was defined as non-susceptibility to at least one agent in three or more antibiotic classes.^39^ Moreover, these agents must be therapeutically relevant and have established breakpoints according to the CLSI for *P. aeruginosa*.^39^ Strains PA86052, PA88949, PA107092, PA108590, PA109084 were assumed to express PDC as they tested negative for carbapenemases (GES, KPC, NDM, IMP, VIM, OXA-48)^24^ and the β-lactam antibiotic MIC being lowered upon addition of class C BLI, avibactam. Lastly, CRPA carrying a carbapenemase being known to be very rare (0.8%) in Canada.^57^ MBL-carriers PA86056 (VIM-2, IMP-18), PA93654 (VIM-4), PA259-96918 (IMP-18), PA106046 (VIM-4) were detected by PCR for notable carbapenemases (GES, KPC, NDM, IMP, VIM, OXA-48); specific gene variants were identified by DNA sequencing.^45^

#### Susceptibility assay

To assess the bacterial susceptibility, antibiotic susceptibility testing was performed using the broth microdilution method with 96-well microtiter plates.^58^ A stock culture of bacteria was incubated overnight in a culture tube with lysogeny broth (LB) and 250 rpm shaking. The culture was grown overnight and adjusted to a turbidity of 0.5 McFarland using 0.85% saline solution. The solution was further diluted with cation-adjusted Mueller–Hinton broth (CAMHB) to resemble a 1 : 50 bacterial solution : CAMHB ratio. Using 96-well plates, 128 μg ml^−1^ of the test compound was placed into the first well in each row. The concentration of test compound was then serially diluted by 2-fold along the row. The 1 : 50 bacterial solution was then placed into each well and incubated overnight for 18 hours at 37 °C. The MIC was determined manually by marking each well as either growth or no growth. An EMax Plus microplate reader (Molecular Devices, USA) operating at a 595 nm wavelength was used to verify the observed MIC. MICs were repeated at least twice on separate days to ensure accurate and reproducible MIC values.

#### Checkerboard assay

To determine synergy between two agents, a checkerboard assay was employed using 96-well plates according to a previous procedure.^59^ The antibiotic was serially diluted 2-fold across the *x*-axis, and the adjuvant along the *y*-axis, resulting in varying concentrations of the antibiotic and adjuvant in the resulting wells. The bacterial culture grown from the previous day was diluted with saline solution to 0.5 McFarland turbidity. This suspension was then further diluted to achieve a 1 : 50 bacterial : CAMHB solution (approximately 5 × 10^5^ CFU mL^−1^). The wells were then inoculated with this 1 : 50 solution and incubated for 18 hours at 37 °C. Plates were read manually by marking each well as growth or no growth. Then, an EMax Plus microplate reader (Molecular Devices, USA) operating at a 595 nm wavelength verified the empirical observation. Subsequently, fold-reduction in MIC and the FIC index of the antibiotic combinations were determined. The FIC index was calculated by dividing the MIC of the antibiotic/adjuvant combination by the MIC of the antibiotic alone to determine the FIC of the antibiotic. Then the FIC of the adjuvant was obtained by dividing the MIC of the combination by the MIC of the adjuvant. The FIC_antibiotic_ and FIC_adjuvant_ were then summed, and the resulting value was the FIC index. Synergy was reported as FIC indices ≤ 0.5, additive effects as 0.5 < *x* ≤ 4 and antagonism as FIC indices > 4.^42^ Checkerboard assays were repeated to ensure accurate and reproducible MICs (within 2-fold agreement according to previous and current data).

#### Time-kill assay

The protocol was adapted from a previous publication.^47^ PA107092 was grown in LB overnight. Cell density was adjusted to OD = 0.5, by dilution of the bacterial culture with phosphate buffer solution (PBS). Compound 4 (8 μg mL^−1^), AVI (2 μg mL^−1^) and β-lactam antibiotics CAZ or ATM (8 μg mL^−1^) were incorporated into the 0.5 McFarland (1 : 50 diluted bacterial culture) medium and LB. The culture tubes were incubated at 37 °C, whilst shaking (250 rpm) for several time intervals. At each interval, a 50 μl aliquot was taken from each culture tube, then serially diluted in PBS, and streaked on LB agar plates. The plates were then incubated for 20-hours wherein the bacterial colonies were counted. If a ≥3 log_10_ reduction of colonies from the initial inoculum was observed, the combination was deemed to display bactericidal activity and <3-fold reduction on a logarithmic scale was determined as bacteriostatic activity.^60,61^

#### Cell viability assay

The cell viability assay was performed against RPTEC and HK-2 cells, as previously described.^30^ The RPTEC cells were cultured in T75 flasks with Dubelcco's Modified Eagle's Medium:F12 supplemented with the human telomerase reverse transcriptase immortalized RPTEC growth kit from ATCC, G418 (0.1 mg mL^−1^ final concentration), and 2% fetal bovine serum (FBS). The HK-2 cells were cultured in T75 flasks with Keratinocyte Serum Free medium supplemented with bovine pituitary extract, human epidermal growth factor, and 2% FBS. The cells were incubated at 5% CO_2_ in a humidified atmosphere at 37 °C. Fifty μL of media containing approx. 8000 cells for RPTEC and approx. 5000 cells for HK-2 were added to designated wells in a 96-well plate. Meanwhile, wells that contained only media served as blanks. After a 24-hour incubation, the wells containing cells, as well as the blank wells, were treated with 50 μL of agent (at 2× the desired concentration). After a 48-hour incubation, PrestoBlue reagent from Invitrogen (10% v/v final concentration) was added to the wells, and the plate was incubated for an additional 1 hour. Fluorescence was measured using the SpectraMax M2 plate reader (Molecular Devices, USA) at excitation and emission wavelengths of 560 and 590 nm, respectively. Values from the blank wells were subtracted from the corresponding wells with cells. The cell viability relative to the controls with vehicle was calculated. The plots indicate the mean ± standard deviation of at least two experiments with five replicates each.

#### Mode of action study

A checkerboard assay was completed with compound 4 and the ansamycin antibiotic, RIF against overnight grown PAO1 in both normal CAMHB and CAMHB with elevated Mg^2+^ concentration. The potentiation of RIF was diminished in elevated Mg^2+^ CAMHB. This effect has been demonstrated in other experimental compounds with known outer membrane effects, such as compound 7.^27^

## Author contributions

FS and CL designed the experiments. CL conducted the synthetic work for compounds 1–6. GK conducted the synthetic work for compound 7. CL characterized the compounds. CL wrote the manuscript and supplementary information with input of all other authors. CL designed the graphical abstract. CL conducted the MIC testing. CL performed the checkerboard assays. DMR trained CL on checkerboard assays. CL performed the time-kill assays. DR trained CL on the time-kill assay. DMR performed the mode-of-action study. GZ reviewed the microbiological assays. GZ provided the bacterial strains. DR performed the cytotoxicity study. RA assisted in the cytotoxicity assays. GA supervised the cytotoxicity study. FS, GK, RA, DMR, DR, GZ and GA reviewed the manuscript.

## Conflicts of interest

The author(s) declare no conflicts of interest.

## Supplementary Material

MD-OLF-D5MD00375J-s001

## Data Availability

The data supporting this article have been included as part of the ESI.†
