# Supplementary material for: Amphiphilic nebramine analogs synergize with β-lactam/β-lactamase inhibitor combinations, including cefepime–taniborbactam and meropenem–xeruborbactam against metallo-β-lactamase-carrying Pseudomonas aeruginosa
Source: RSC Med Chem. 2025 Jul 17;16(9):4492–509. doi: 10.1039/d5md00375j (PMC12302231; doi:10.1039/d5md00375j)
Supplement: MD-016-D5MD00375J-s001 [file MD-016-D5MD00375J-s001.pdf]

Supplementary Information (SI) for RSC Medicinal Chemistry.
This journal is © The Royal Society of Chemistry 2025

Supplementary Information

Amphiphilic Nebramine Analogs Synergize with β-Lactam/β-Lactamase Inhibitor Combinations, Including Cefepime-Taniborbactam and Meropenem-Xeruborbactam Against Metallo-β-Lactamase-Carrying *Pseudomonas aeruginosa*

Authors: Christian Lozeau[a], Danzel Ramirez[a], Danyel Ramirez[a], Gulshan Kumar[a], Rajat Arora[a] George Zhanel[b], Gilbert Arthur[c], Frank Schweizer*[ab].

Affiliations: [a]Department of Chemistry, University of Manitoba, Winnipeg, MB R3T 2N2, Canada; lozeauc@myumanitoba.ca, ramirezd@myumanitoba.ca, ramiredm@myumanitoba.ca, gulshan.kumar@umanitoba.ca, arorar5@myumanitoba.ca

[b]Department of Medical Microbiology and Infectious Diseases, University of Manitoba, Winnipeg, MB, R3E 0J9, Canada; ggzhanel@pcsinternet.ca

[c]Department of Biochemistry and Medical Genetics, University of Manitoba, Winnipeg, MB, R3E 0J9, Canada; gilbert.arthur@umanitoba.ca

*Correspondence: frank.schweizer@cc.umanitoba.ca

Table of Contents

Microbiology

Current CLSI breakpoints 3

Susceptibility profile of *P. aeruginosa* isolates 4

Additional synergy data for compound 2 .... 5 - 8

Synergy data for comparison .... 9 - 10

Comparison of compound 4 vs. compound 2 9

Comparison of compound 4 vs. compound 7 10

Chemistry

1H, 13C, HSQC, HMBC, COSY, and DEPT135 NMR of compounds 1 – 6 .... 11 - 46

1H and 13C NMR of compound 7 and intermediates .... 47 - 62

References 63

Current CLSI Breakpoints

Table S1. Current CLSI breakpoints (2024) of β-lactam antibiotics against *P. aeruginosa* by the broth microdilution method in CAMHB.[1]

	***P. aeruginosa* MIC Breakpoints (μg/ml)**		
Monobactam(s)	Susceptible (S)	Intermediate (I)	Resistant (R)
ATM	≤ 8	16^	≥ 32
Cephem(s)			
CAZ	≤ 8	16^	≥ 32
FEP	≤ 8	16^	≥ 32
Carbapenem(s)			
MEM	≤ 2	4^	≥ 8

Table S2. Susceptibility profile of *P. aeruginosa* isolates used in this study.[2]

	Minimum Inhibitory Concentration, MIC (µg/mL)																		
***P. aeruginosa* Strain**	**TZP[b]**	**CRO**	**CAZ**	**CAZ-AVI[a]**	**C/T[b]**	**FEP**	**FEP-TAN[a]**	**FEP-XER[a]**	**IPM**	**MEM**	**MEM-VAB[a]**	**MEM-XER[a]**	**ATM**	**ATM-AVI[a]**	**TOB**	**GEN**	**CIP**	**TGC**	**CST**
PAO1	8	ND	4	ND	0.5	2	ND	ND	ND	1	ND	ND	4	ND	1	1	0.125	4	1
PA101243	128	>64	512	ND	1	64	ND	ND	16	16	ND	ND	256	ND	128	>32	1	ND	1024
PA114228	ND	ND	8	ND	ND	4	ND	ND	ND	16	ND	ND	16	ND	2	ND	ND	ND	4
PA262-101856	64	64	16	ND	1	32	ND	ND	32	32	ND	ND	32	ND	1024	>32	>16	32	1
PA264-104354	256	>64	64	ND	2	64	ND	ND	32	64	ND	ND	64	ND	128	>32	>16	32	1
PA86052	256	>64	256	32	2	64	16	32	32	16	16	16	64	8	1	4	>16	ND	1
PA88949	256	>64	128	8	2	32	8	16	>32	32	32	16	32	16	4	16	4	ND	2
PA107092	512	>64	64	64	2	64	8	32	32	64	64	64	128	128	>64	>32	>16	ND	1
PA108590	256	>64	64	4	2	32	8	16	32	16	8	8	64	4	4	16	4	ND	1
PA109084	512	>64	128	8	2	32	8	16	32	16	16	16	128	16	4	16	4	ND	1
PA86056	128	>64	512	512	>64	128	128	128	>32	128	128	64	16	16	>64	8	>16	>16	1
PA93654	512	>64	128	128	>64	128	16	32	>32	512	512	256	16	16	>64	>32	>16	16	1
PA259-96918	128	>64	512	512	>64	512	256	256	32	1024	1024	1024	32	32	>64	>32	>16	>16	0.5
PA106046	>512	>64	128	16	4	32	16	16	16	4	4	2	32	4	32	>32	1	8	0.5

ND = Not determined; TZP = piperacillin-tazobactam; CRO = ceftriaxone; CAZ = ceftazidime; CAZ-AVI = ceftazidime-avibactam; C/T = ceftolozane-tazobactam; FEP = cefepime; FEP-TAN = cefepime-taniborbactam; FEP-XER = cefepime-xeruborbactam; IPM = imipenem; MEM = meropenem; MEM-VAB = meropenem-vaborbactam; MEM-XER = meropenem-xeruborbactam; ATM = aztreonam; ATM-AVI = aztreonam-avibactam; TOB = tobramycin; GEN = gentamicin; CIP = ciprofloxacin; TGC = tigecycline; CST = colistin; Wild-type *P. aeruginosa* = PAO1 (blue); multi/extensively drug-resistant *P. aeruginosa* (MDR) = PA101243, PA114228, PA262-101856, PA264-104354; PDC-expressing *P. aeruginosa* = PA86052, PA88949, PA107092, PA108590, PA109084; MBL-carrying *P. aeruginosa* = PA86056 (VIM-2 and IMP-18), PA93654 (VIM-4), PA259-96918 (IMP-18), PA106046 (VIM-4), [a] = BLI fixed at 2 µg/mL; [b] = BLI fixed at 4 µg/mL.

Additional Compound 2 Synergy Data

Table S3. Synergy determination of compound **2** + ATM or CAZ dual combinations against MDR *P. aeruginosa*.

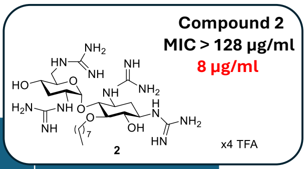

Strain	Antibiotic	MIC antibiotic alone (µg/ml)	MIC comb (µg/ml)	Fold Potentiation	FIC index
PA 101243	ATM	256 (R)	256 (R)	1	0.5 < x < 0.56
	CAZ	512 (R)	256 (R)	2	0.5 < x < 0.56
PA 114228	ATM	16 (I)	8 (S)	2	0.5 < x < 0.56
	CAZ	4 (S)	4 (S)	1	1 < x < 1.06
PA 259-96918	ATM	32 (R)	8 (S)	4	0.25 < x < 0.313
	CAZ	512 (R)	128 (R)	4	0.25 < x < 0.313
PA 262	ATM	32 (R)	8 (S)	4	0.25 < x < 0.313
	CAZ	16 (I)	4 (S)	4	0.25 < x < 0.313
PA 264	ATM	64 (R)	16 (I)	4	0.25 < x < 0.313
	CAZ	64 (R)	16 (I)	4	0.25 < x < 0.313

Table S4. Synergy determination of compound **2** + MEM dual combinations against MDR *P. aeruginosa*.

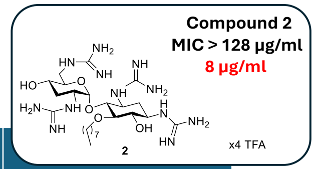

Strain	Antibiotic	MIC antibiotic alone (µg/ml)	MIC comb (µg/ml)	Fold Potentiation	FIC index
PA 101243	MEM	16 (R)	8 (R)	2	0.5 < x < 0.56
PA 114228	MEM	16 (R)	8 (R)	2	0.5 < x < 0.56
PA 259-96918	MEM	1024 (R)	256 (R)	4	0.25 < x < 0.313
PA 262	MEM	32 (R)	8 (R)	4	0.25 < x < 0.313
PA 264	MEM	64 (R)	16 (R)	4	0.25 < x < 0.313

Table S5. Synergy determination of compound **2** + ATM or CAZ dual combinations against PDC-producing *P. aeruginosa*.

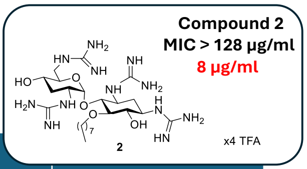

Strain Strain	Antibiotic	MIC Antibiotic alone (μg/ml)	MIC comb (μg/ml)	Fold Potentiation	FIC index
PA 86052	ATM	64 (R)	16 (I)	4	0.25 < x < 0.313
	CAZ	128 (R)	64 (R)	2	0.5 < x < 0.56
PA 88949	ATM	64 (R)	8 (S)	8	0.125 < x < 0.188
	CAZ	64 (R)	16 (I)	4	0.25 < x < 0.313
PA 107092	ATM	64 (R)	8 (S)	8	0.125 < x < 0.188
	CAZ	64 (R)	16 (I)	4	0.25 < x < 0.313
PA 108590	ATM	32 (R)	2 (S)	16	0.063 < x < 0.125
	CAZ	64 (R)	8 (S)	8	0.125 < x < 0.188
PA 109084	ATM	128 (R)	16 (I)	8	0.125 < x < 0.188
	CAZ	64 (R)	16 (I)	4	0.25 < x < 0.313

Table S6. Synergy determination of compound **2** + ATM-AVI or CAZ-AVI triple combinations against PDC-producing *P. aeruginosa*.

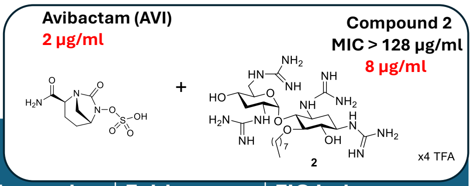

Strain	β-lactam/β-lactamase inhibitor	MIC β-lactam/β-lactamase inhibitor combo (μg/ml)	MIC triple combo (μg/ml)	Fold Potentiation	FIC Index
PA 86052	ATM + AVI	8 (S)	1 (S)	8	0.13 < x < 0.188
	CAZ + AVI	64 (R)	8 (S)	8	0.13 < x < 0.188
PA 88949	ATM + AVI	16 (I)	4 (S)	4	0.25 < x < 0.313
	CAZ + AVI	16 (R)	4 (S)	4	0.25 < x < 0.313
PA 107092	ATM + AVI	64 (R)	8 (S)	8	0.125 < x < 0.188
	CAZ + AVI	32 (R)	4 (S)	8	0.125 < x < 0.188
PA 108590	ATM + AVI	4 (S)	0.125 (S)	32	0.031 < x < 0.094
	CAZ + AVI	4 (S)	0.25 (S)	16	0.063 < x < 0.125
PA 109084	ATM + AVI	16 (I)	4 (S)	4	0.25 < x < 0.313
	CAZ + AVI	16 (R)	4 (S)	4	0.25 < x < 0.313

Table S7. Synergy determination of compound **2** + FEP-TAN triple combinations against PDC-producing *P. aeruginosa*.

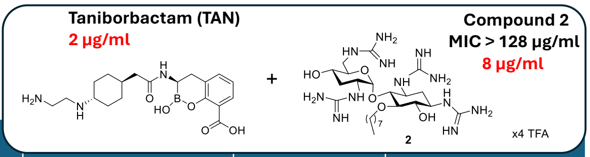

Strain	β-lactam/β-lactamase inhibitor	MIC β-lactam/β-lactamase inhibitor combo (µg/ml)	MIC triple combo (µg/ml)	Fold Potentiation	FIC Index
PA 86052	FEP + TAN	16 (I)	8 (S)	2	0.5 < x < 0.56
PA 88949	FEP + TAN	8 (S)	4 (S)	2	0.5 < x < 0.56
PA 107092	FEP + TAN	16 (I)	2 (S)	8	0.13 < x < 0.188
PA 108590	FEP + TAN	8 (S)	1 (S)	8	0.13 < x < 0.188
PA 109084	FEP + TAN	8 (S)	2 (S)	4	0.25 < x < 0.313

Table S8. Synergy determination of compound **2** + ATM-AVI or CAZ-AVI triple combinations against MBL-carrying *P. aeruginosa*.

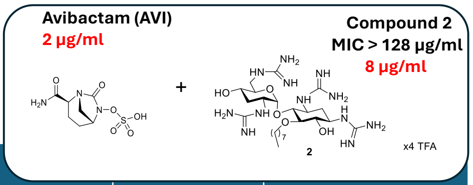

Strain	β-lactam/β-lactamase inhibitor	MIC β-lactam/β-lactamase inhibitor combo (µg/ml)	MIC triple combo (µg/ml)	Fold Potentiation	FIC Index
PA 86056-MBL	ATM + AVI	16 (I)	4 (S)	4	0.25 < x < 0.313
	CAZ + AVI	512 (R)	64 (R)	8	0.125 < x < 0.188
PA 93654-MBL	ATM + AVI	16 (I)	8 (S)	2	0.5 < x < 0.563
	CAZ + AVI	128 (R)	32 (R)	4	0.25 < x < 0.313
PA 259-96918-MBL	ATM + AVI	32 (R)	8 (S)	4	0.25 < x < 0.313
	CAZ + AVI	512 (R)	128 (R)	4	0.25 < x < 0.313
PA 106046-MBL	ATM + AVI	2 (S)	0.25 (S)	8	0.125 < x < 0.188
	CAZ + AVI	4 (S)	0.5 (S)	8	0.125 < x < 0.188

Table S9. Synergy determination of compound **2** + FEP-TAN triple combinations against MBL-carrying *P. aeruginosa*.

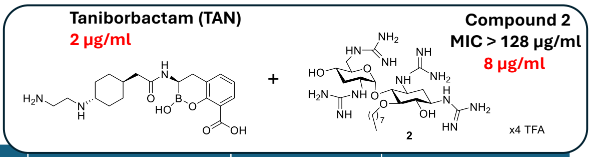

Strain	β-lactam/β-lactamase inhibitor	MIC β-lactam/β-lactamase inhibitor combo (μg/ml)		MIC triple combo (μg/ml)		Fold Potentiation	FIC Index
PA 86056-MBL	FEP + TAN	128	(R)	32	(R)	4	0.25 < x < 0.313
PA 93654-MBL	FEP + TAN	16	(I)	4	(S)	4	0.25 < x < 0.313
PA 259-96918-MBL	FEP + TAN	256	(R)	64	(R)	4	0.25 < x < 0.313
PA 106046-MBL	FEP + TAN	16	(I)	2	(S)	8	0.25 < x < 0.313

45

Synergy data for comparison

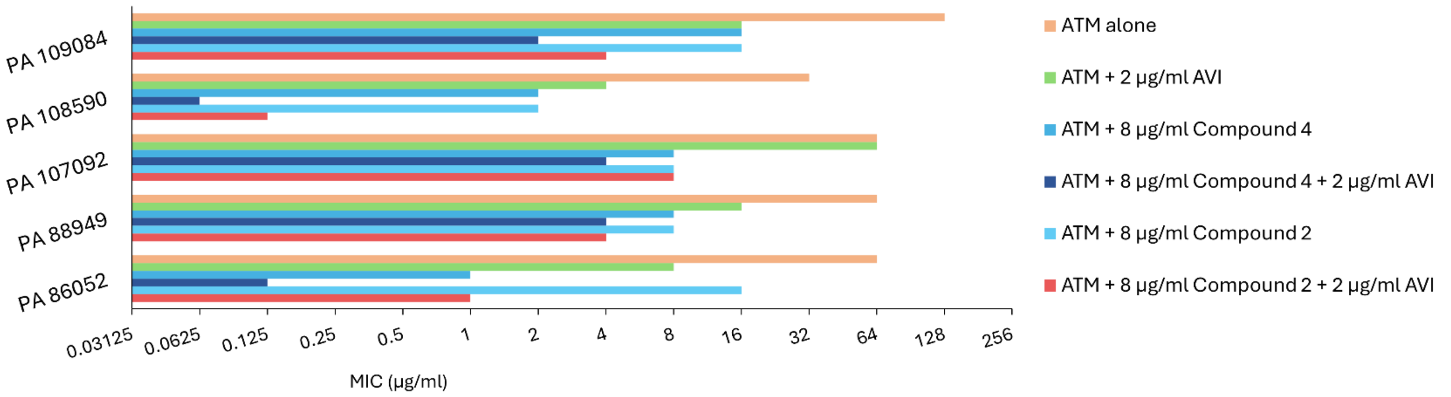

Figure S1. Synergy comparison of compound **4** vs compound **2** + ATM combinations against PDC-producing *P. aeruginosa*.

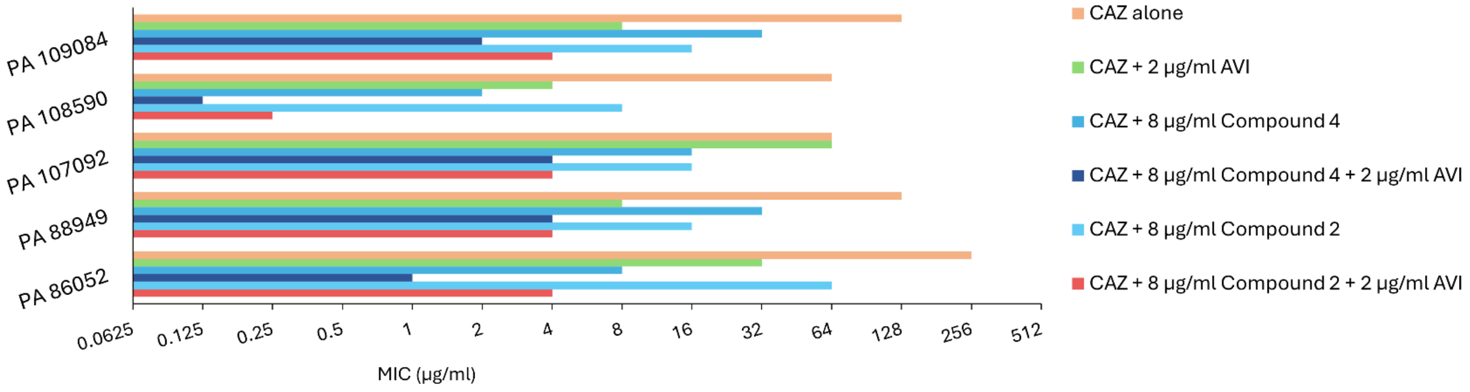

Figure S2. Synergy comparison of compound **4** vs compound **2** + CAZ combinations against PDC-producing *P. aeruginosa*.

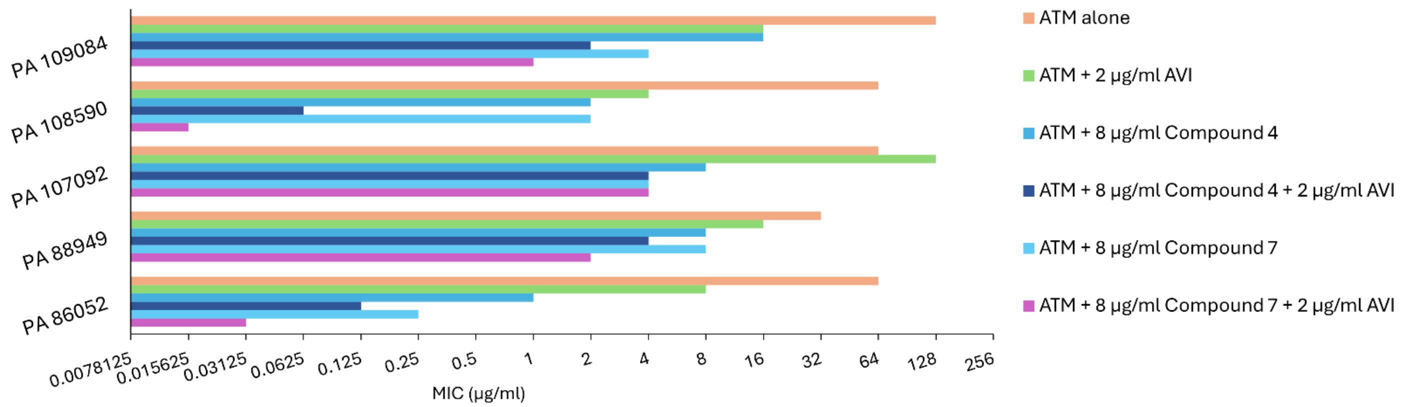

Figure S3. Synergy comparison of compound **4** vs compound **7** + ATM combinations against PDC-producing *P. aeruginosa*.

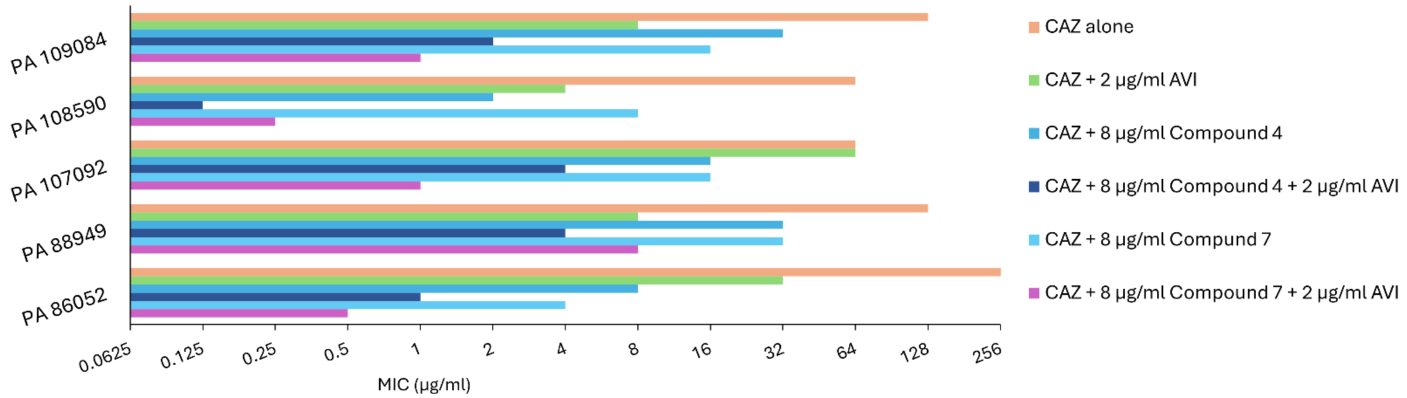

Figure S4. Synergy comparison of compound **4** vs compound **7** + CAZ combinations against PDC-producing *P. aeruginosa*.

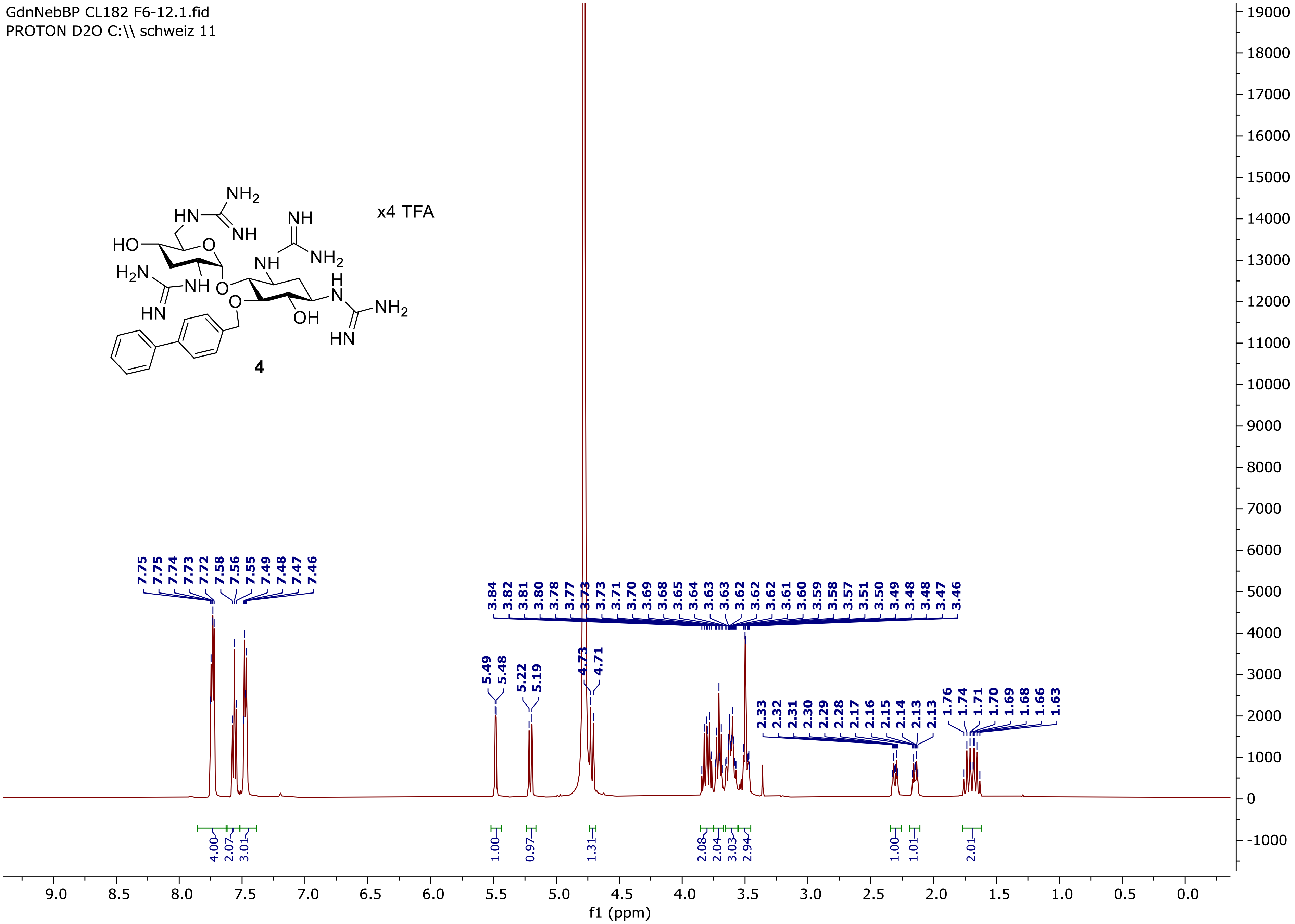

Figure S5. ^{1}H NMR spectrum for compound **4** in D_2O.

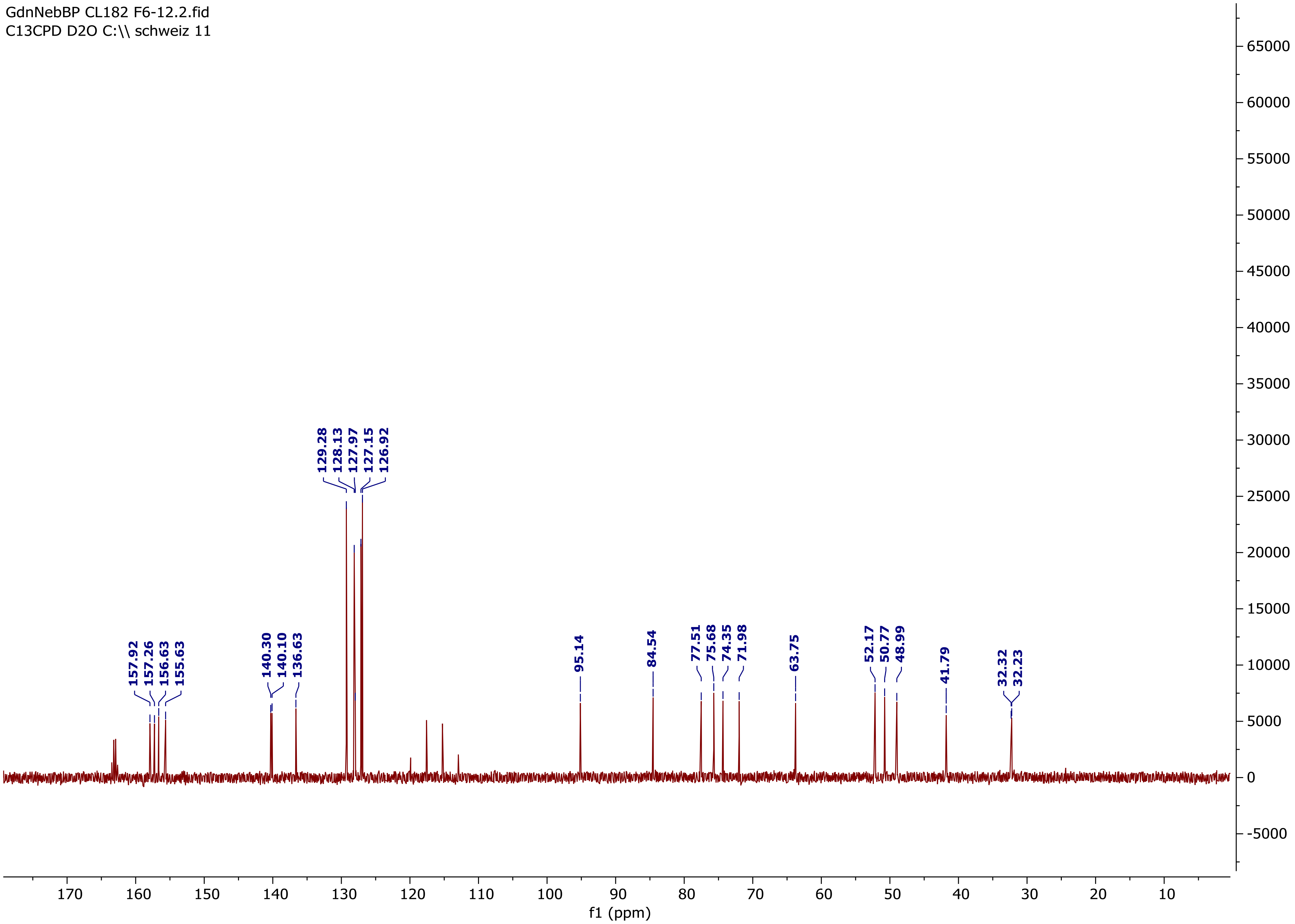

Figure S6. ^{13}C NMR spectrum for compound **4** in D_2O

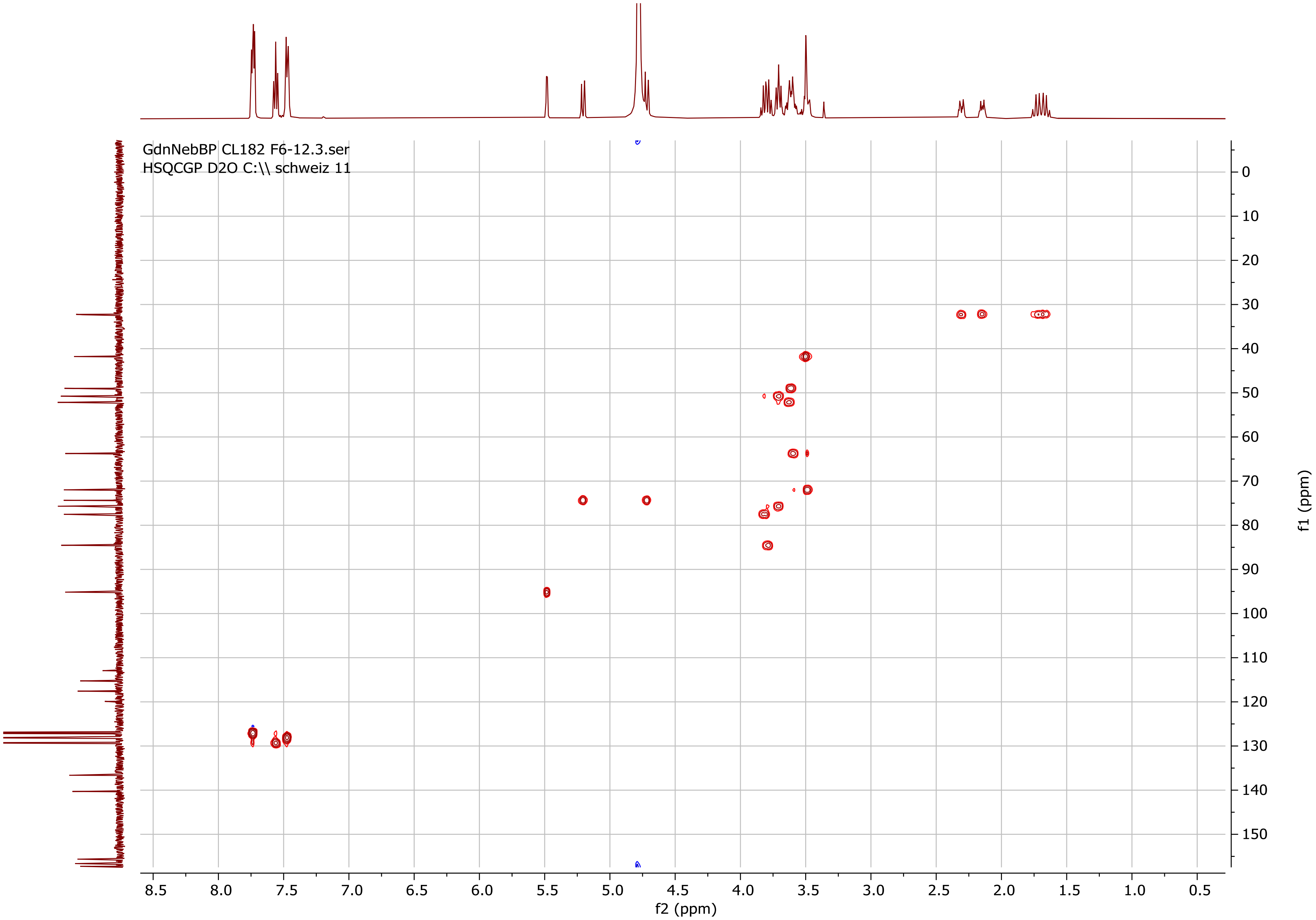

Figure S7. HSQC NMR spectrum for compound **4** in D_2O.

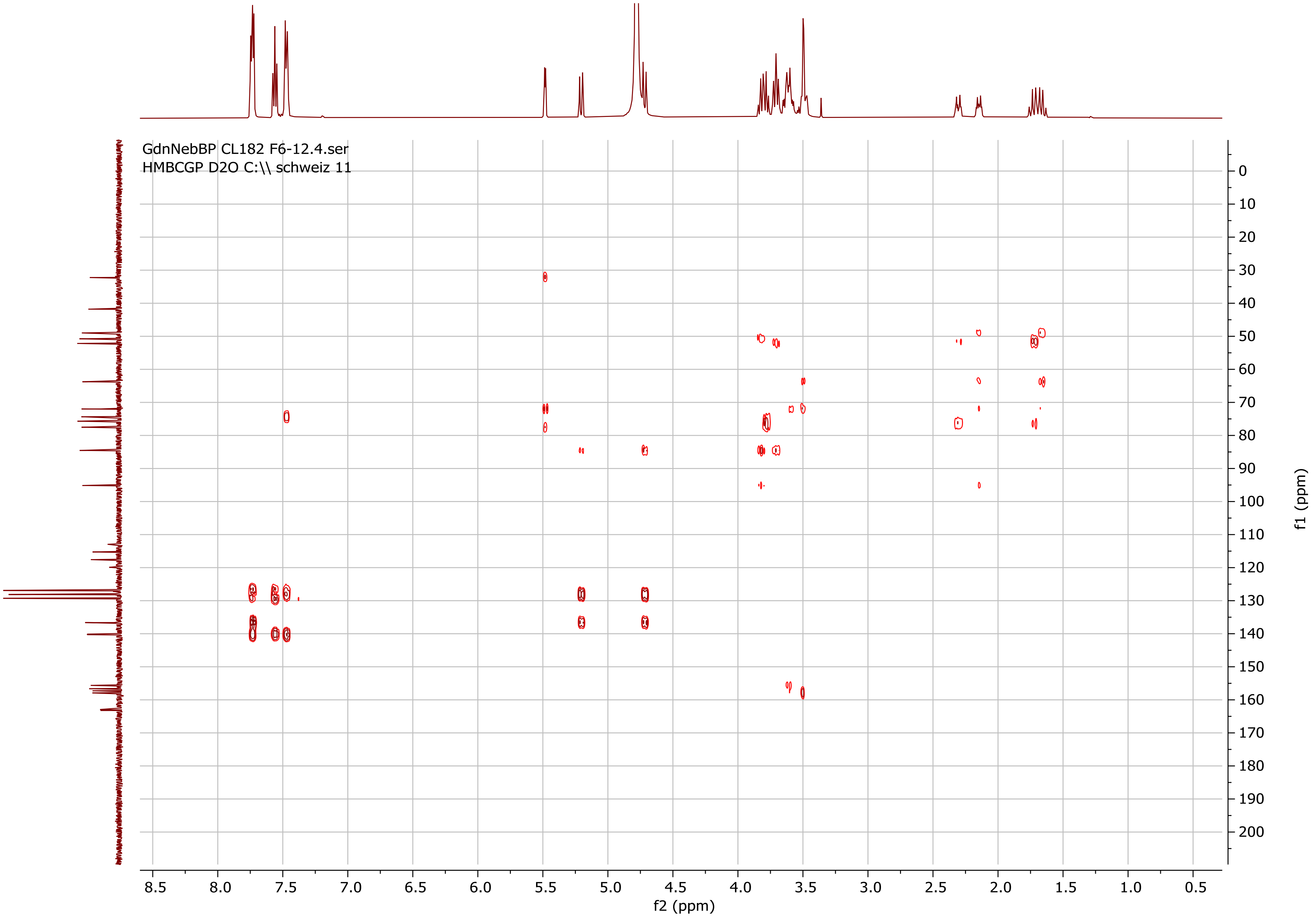

Figure S8. HMBC NMR spectrum for compound **4** in D_2O.

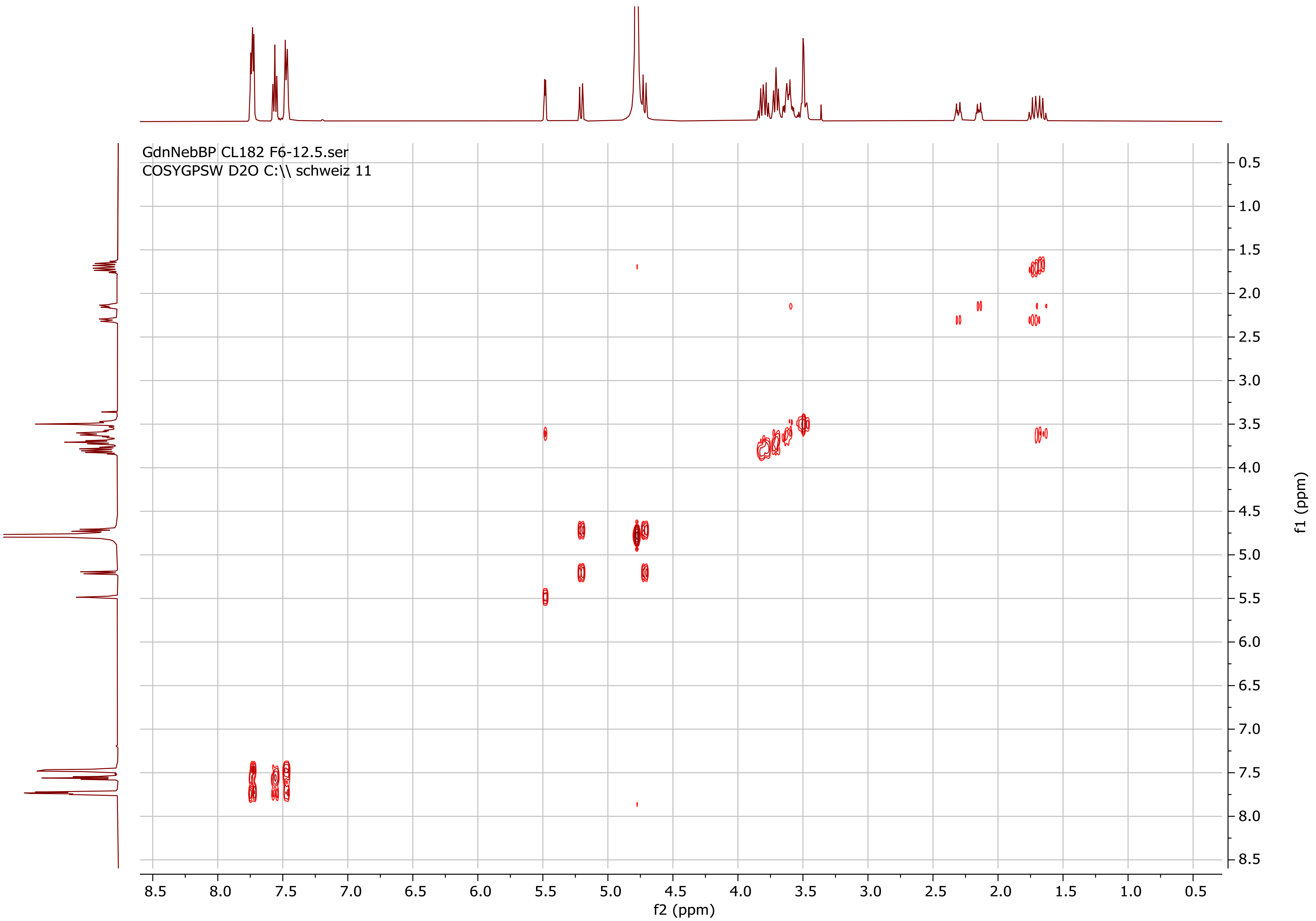

Figure S9. COSY NMR spectrum for compound **4** in D_2O.

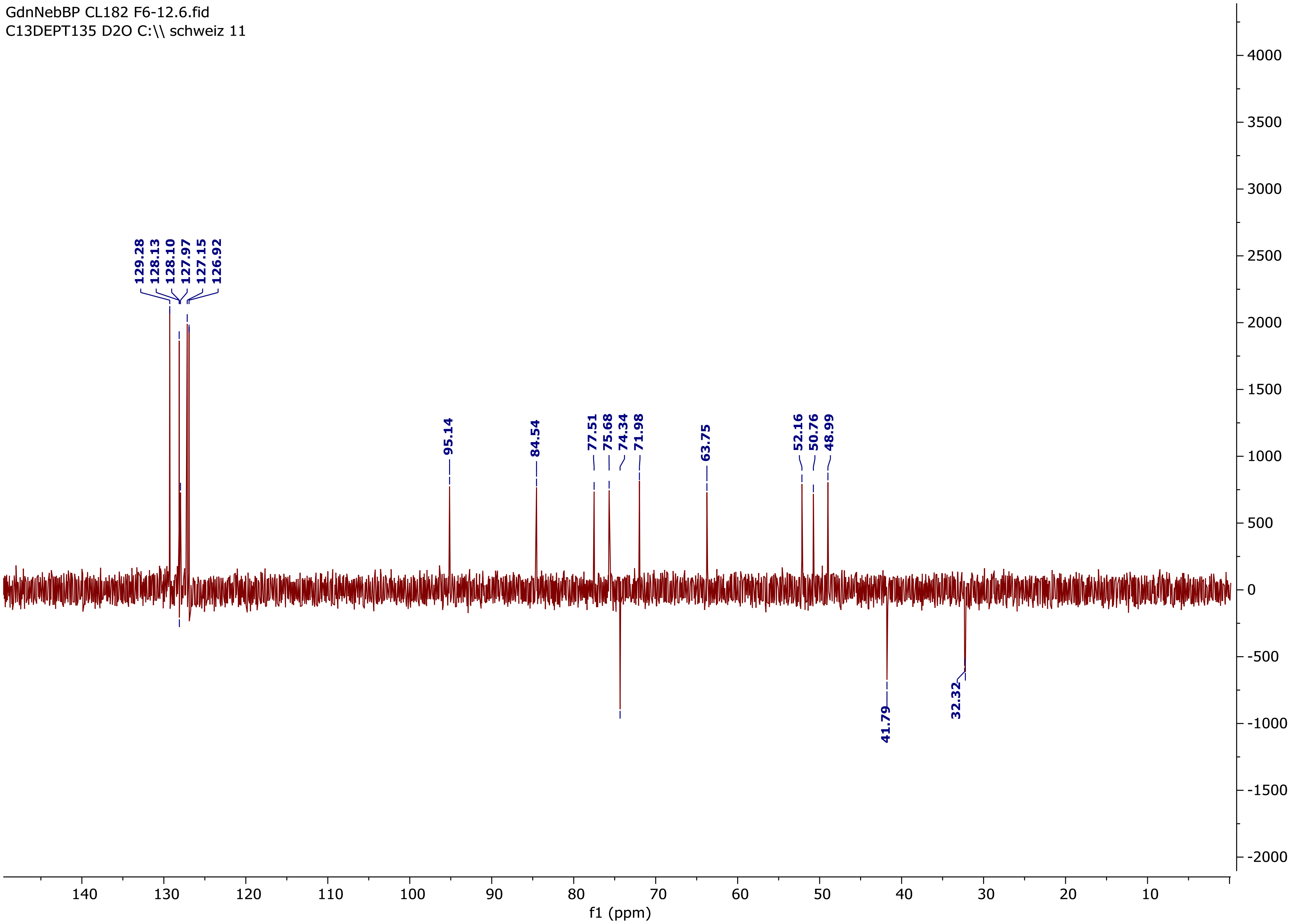

Figure S10. DEPT135 NMR spectrum for compound **4** in D_2O.

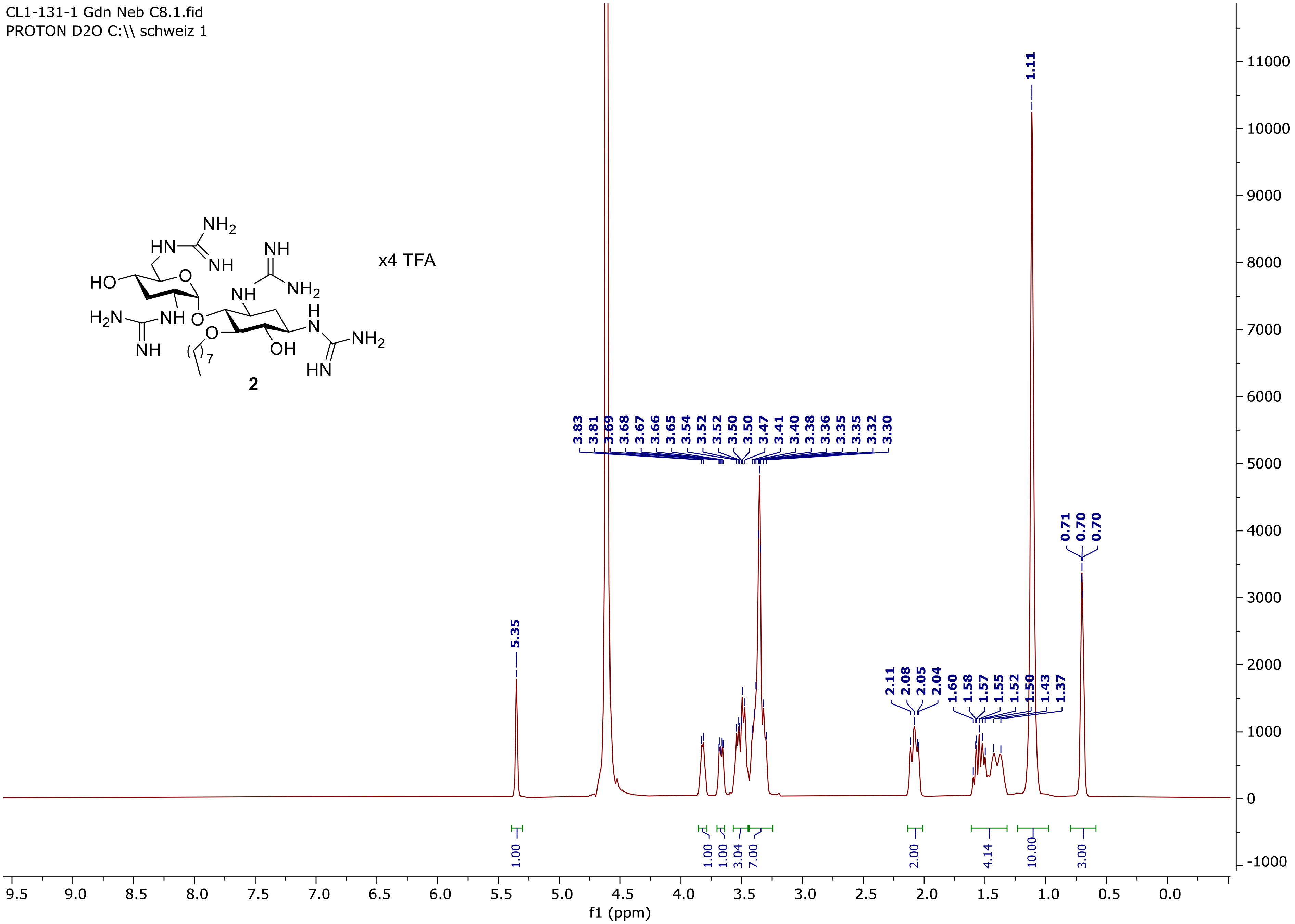

Figure S11. ^{1}H NMR spectrum for compound **2** in D_2O.

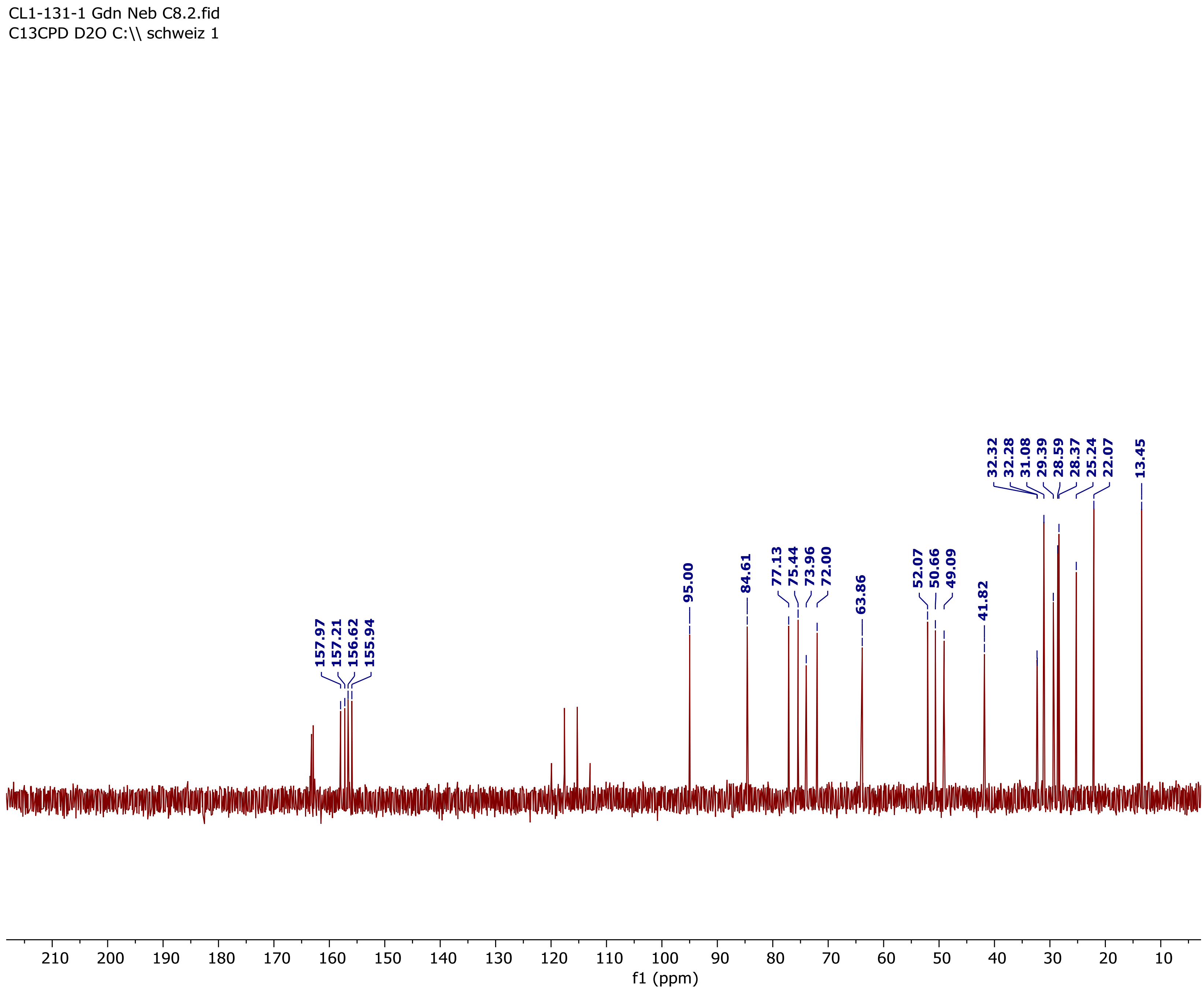

Figure S12. ^{13}C NMR spectrum for compound **2** in D_2O.

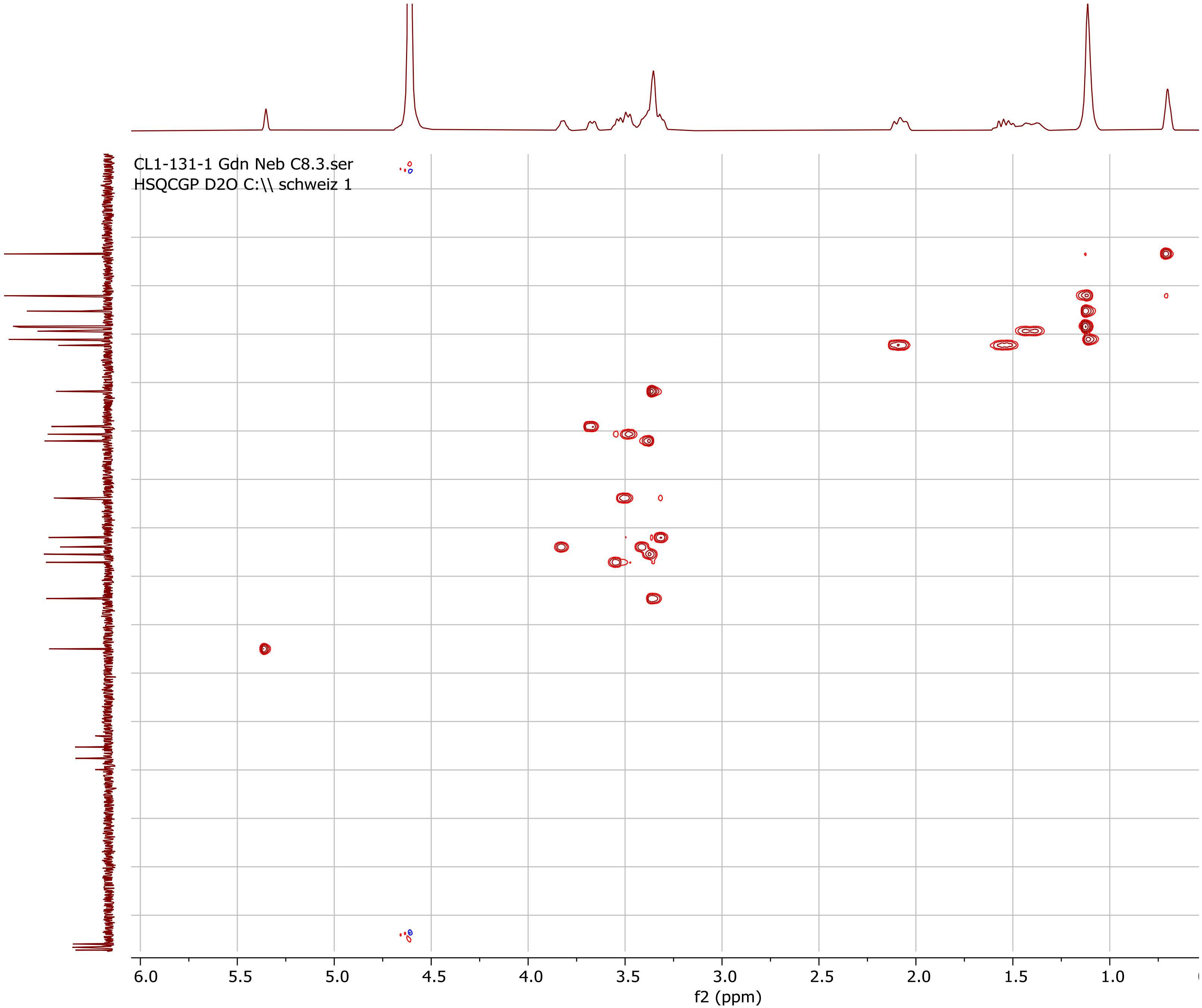

Figure S13. HSQC NMR spectrum for compound **2** in D_2O.

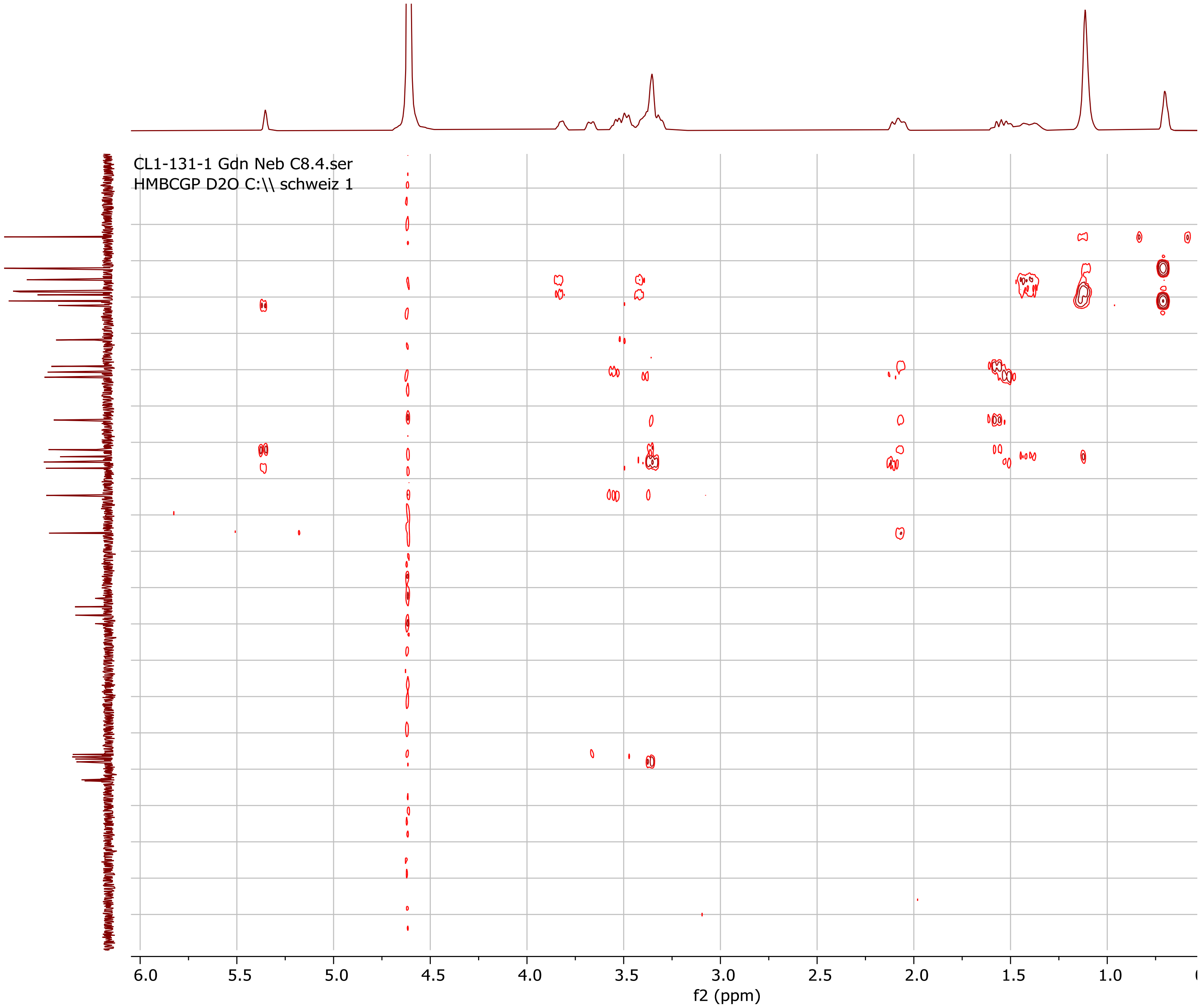

Figure S14. HMBC NMR spectrum for compound **2** in D_2O.

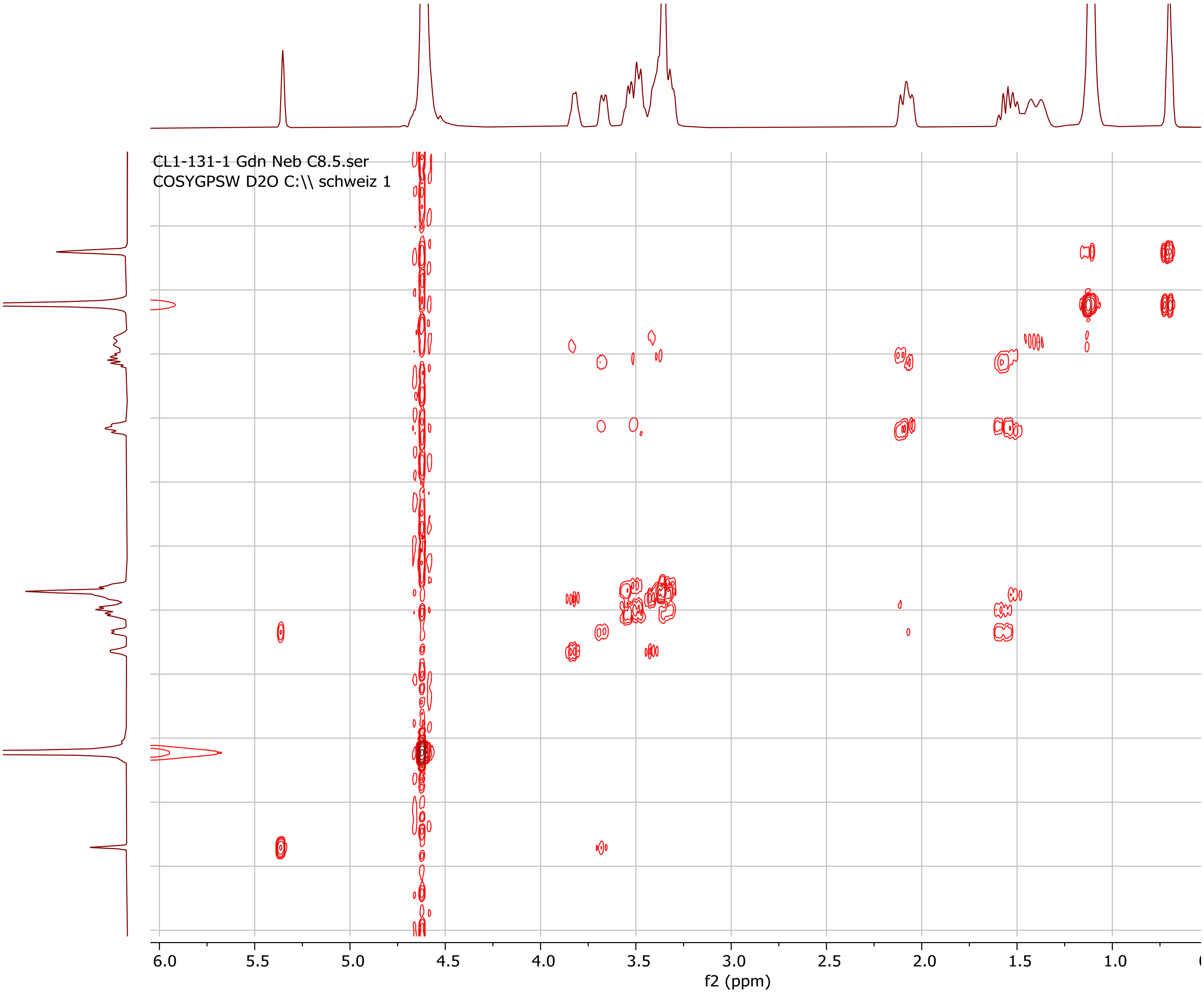

Figure S15. COSY NMR spectrum for compound **2** in D_2O.

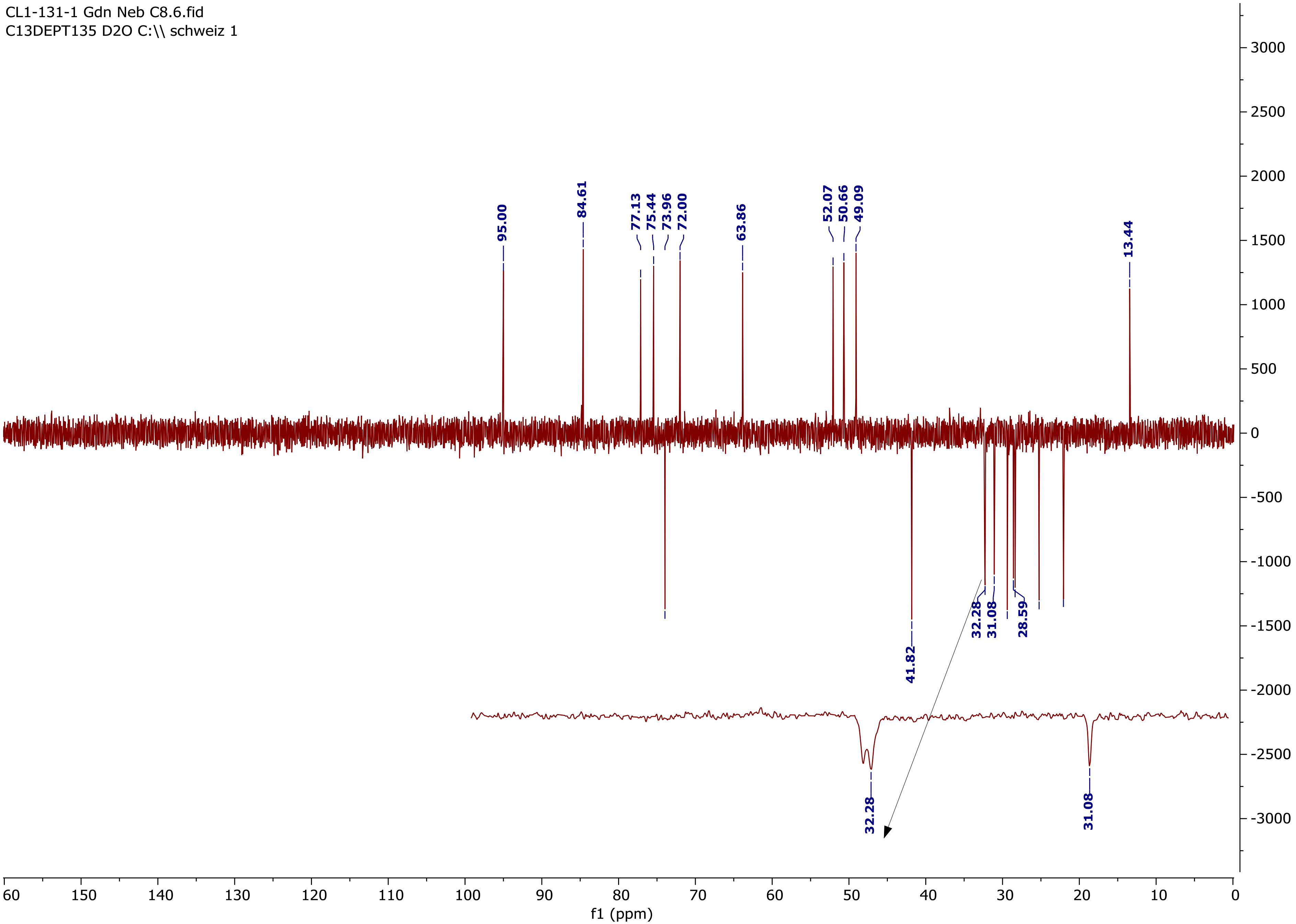

Figure S16. DEPT135 NMR spectrum for compound **2** in D_2O.

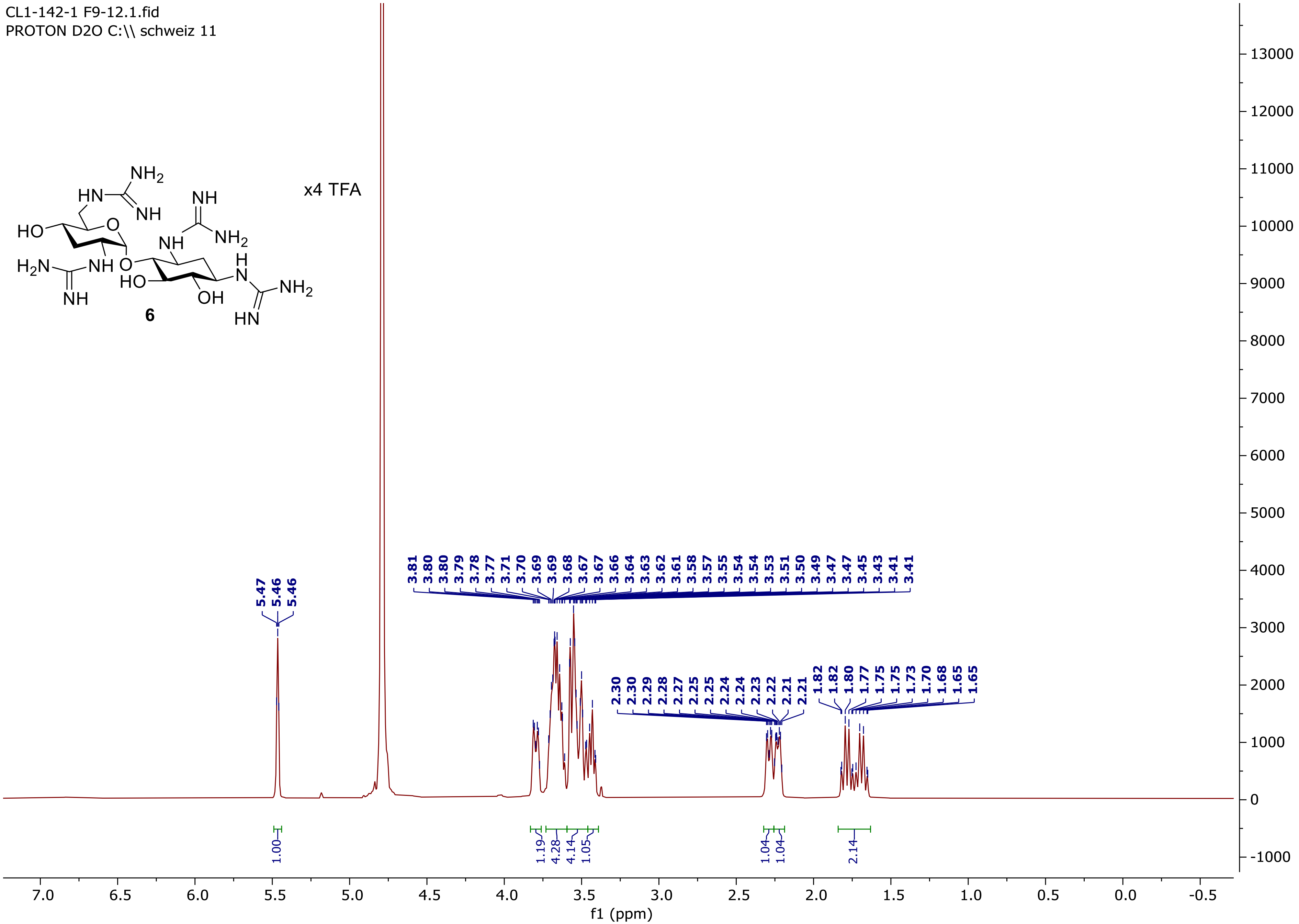

Figure S17. ^{1}H NMR spectrum for compound **6** in D_2O.

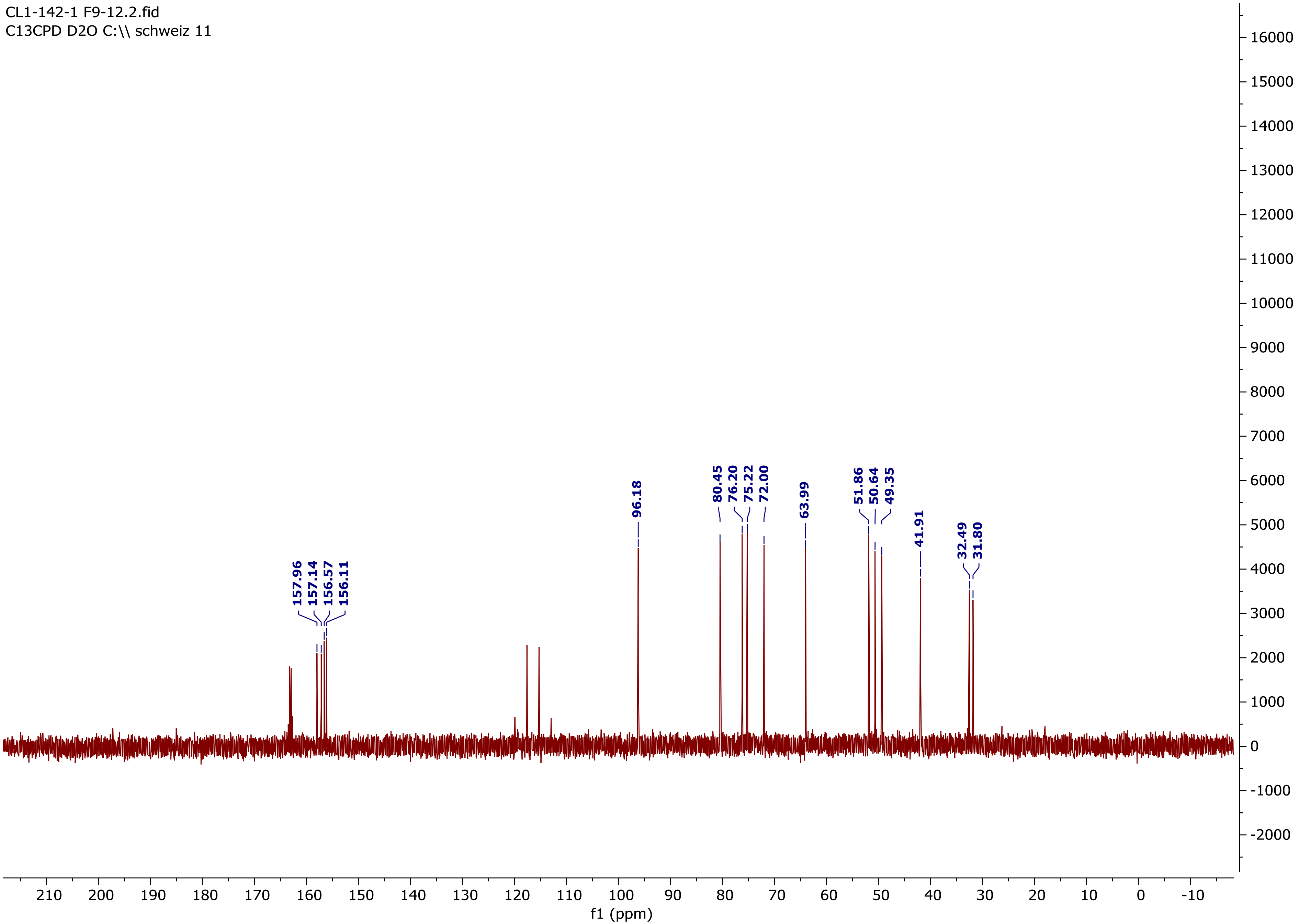

Figure S18. ^{13}C NMR spectrum for compound **6** in D_2O.

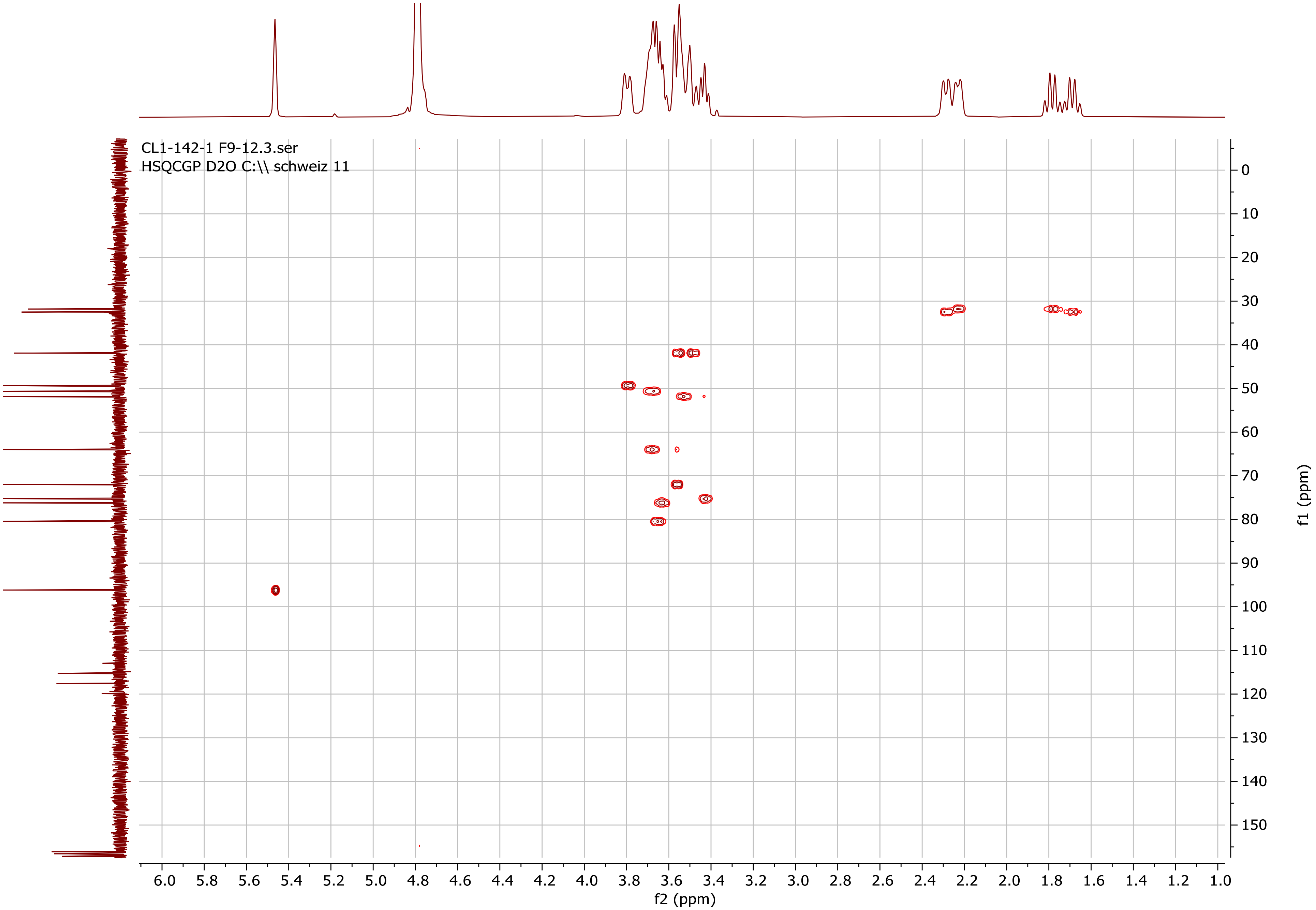

Figure S19. HSQC NMR spectrum for compound **6** in D_2O.

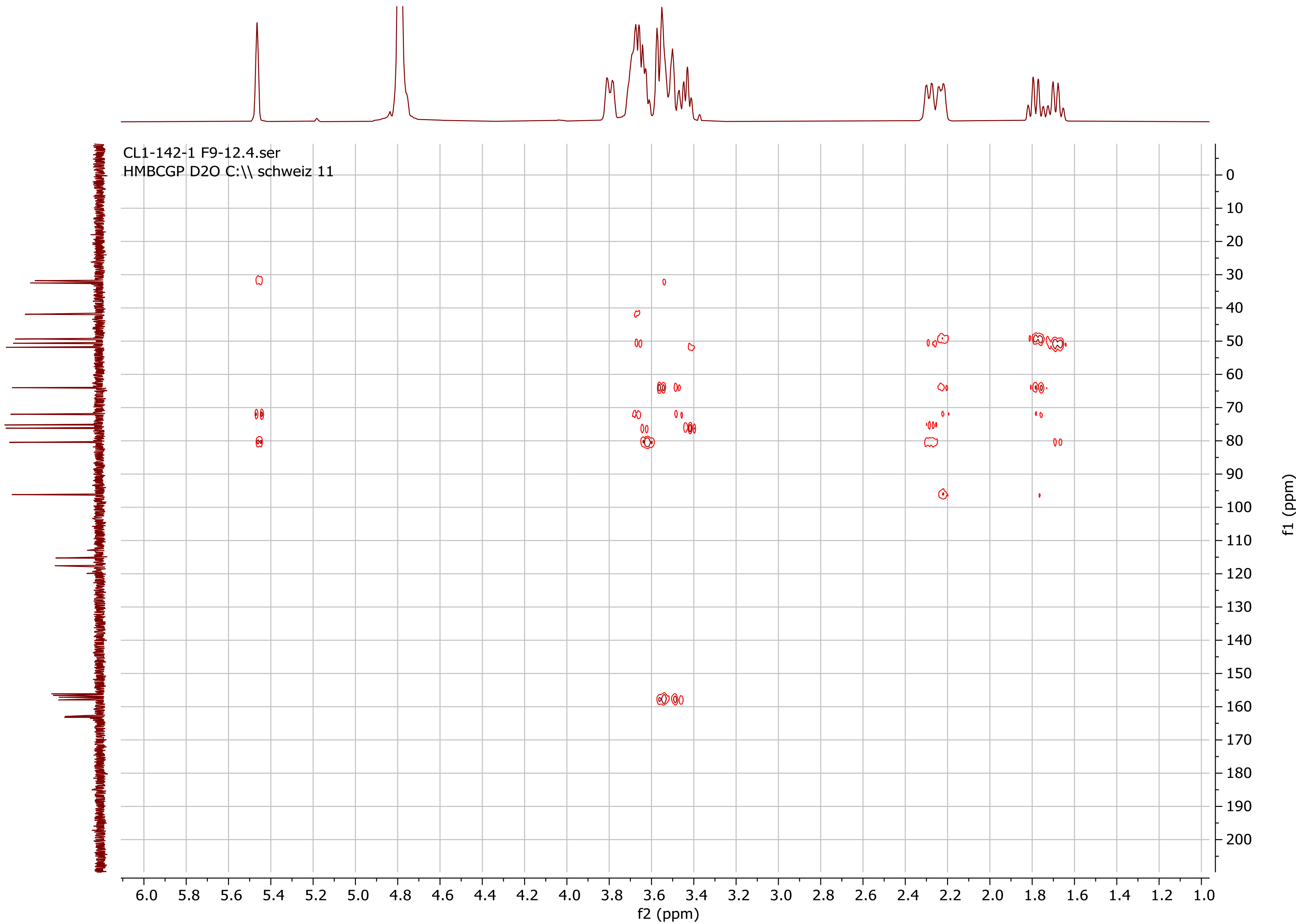

Figure S20. HMBC NMR spectrum for compound **6** in D_2O.

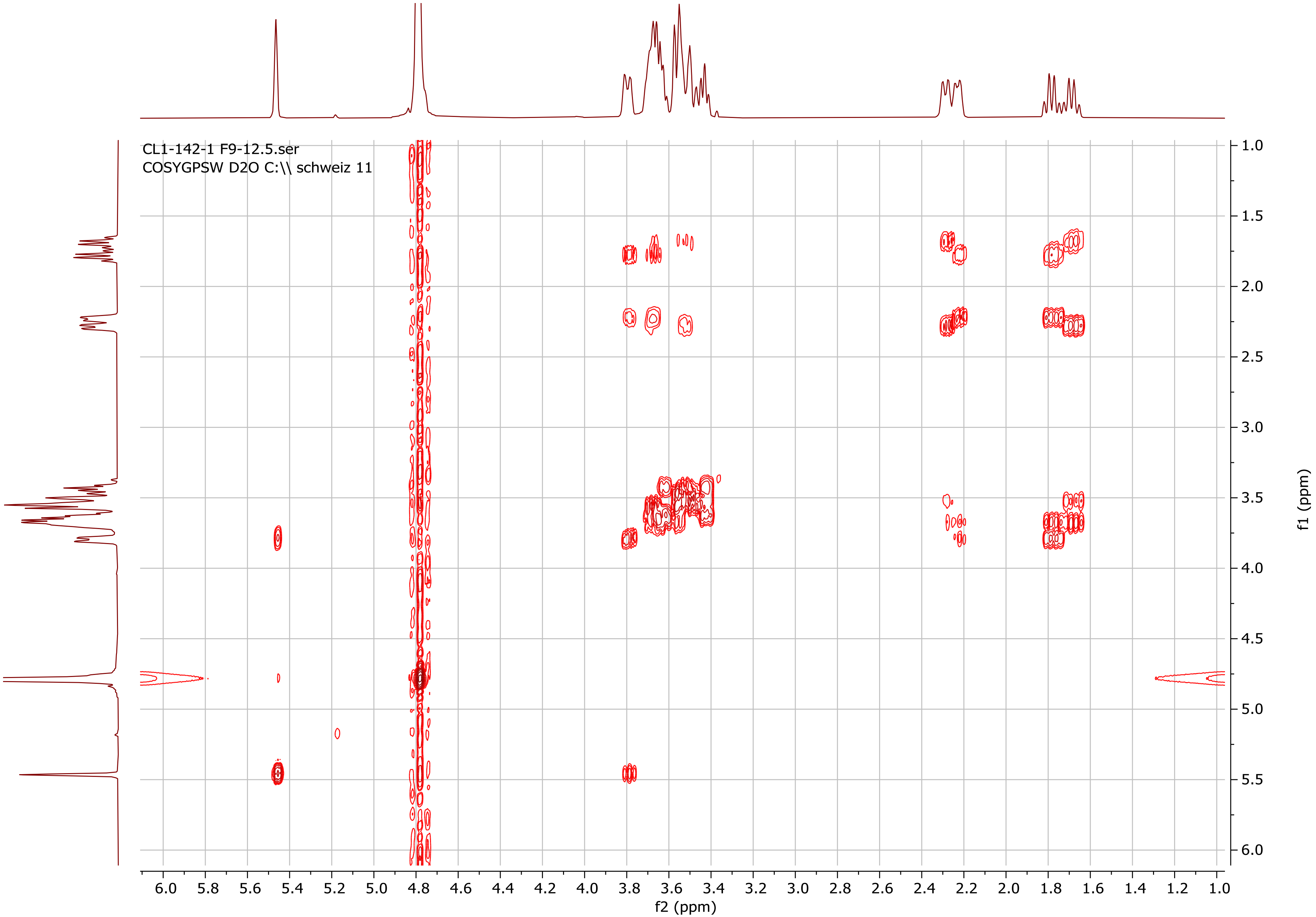

Figure S21. COSY NMR spectrum for compound **6** in D_2O.

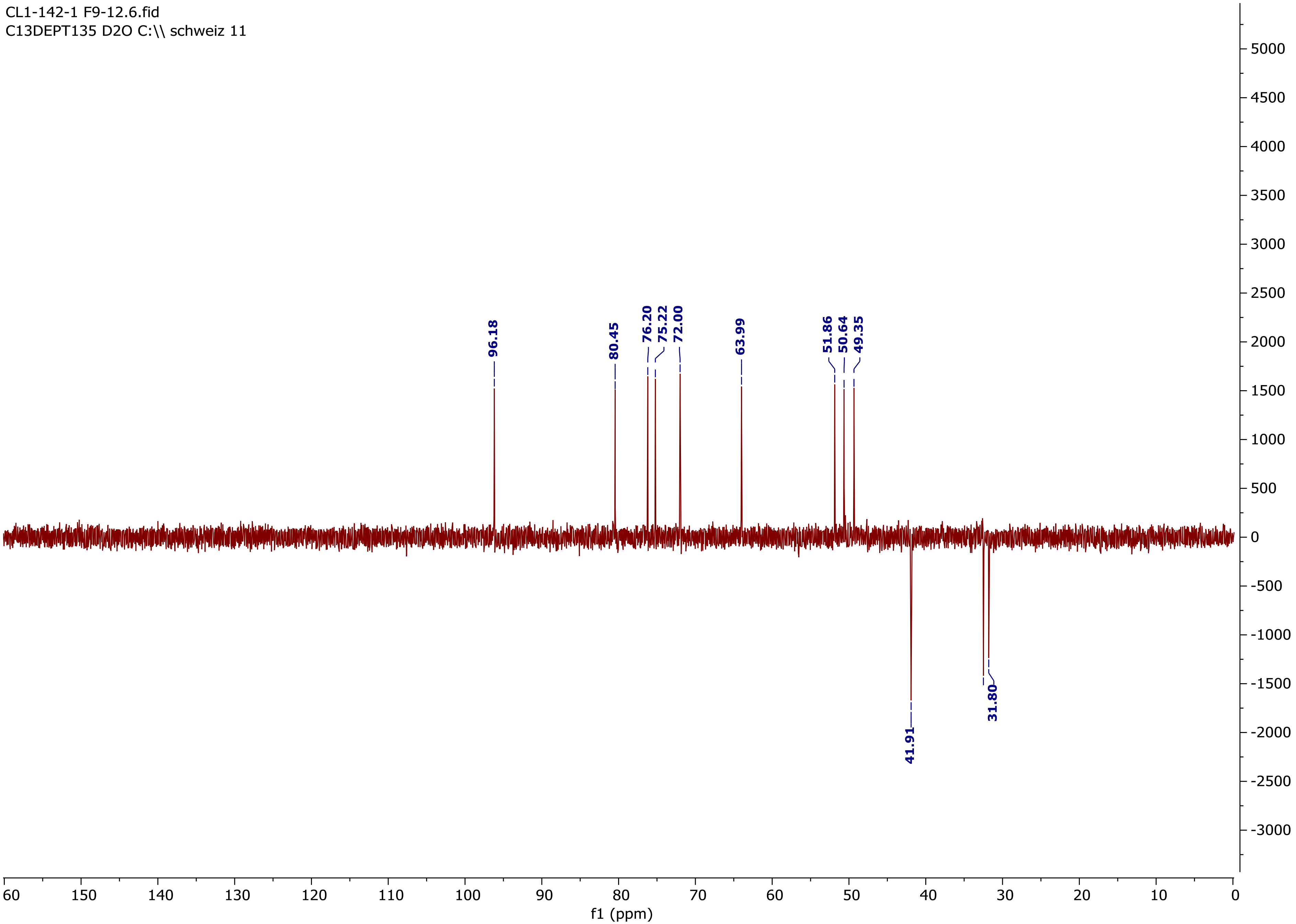

Figure S22. DEPT135 NMR spectrum for compound **6** in D_2O.

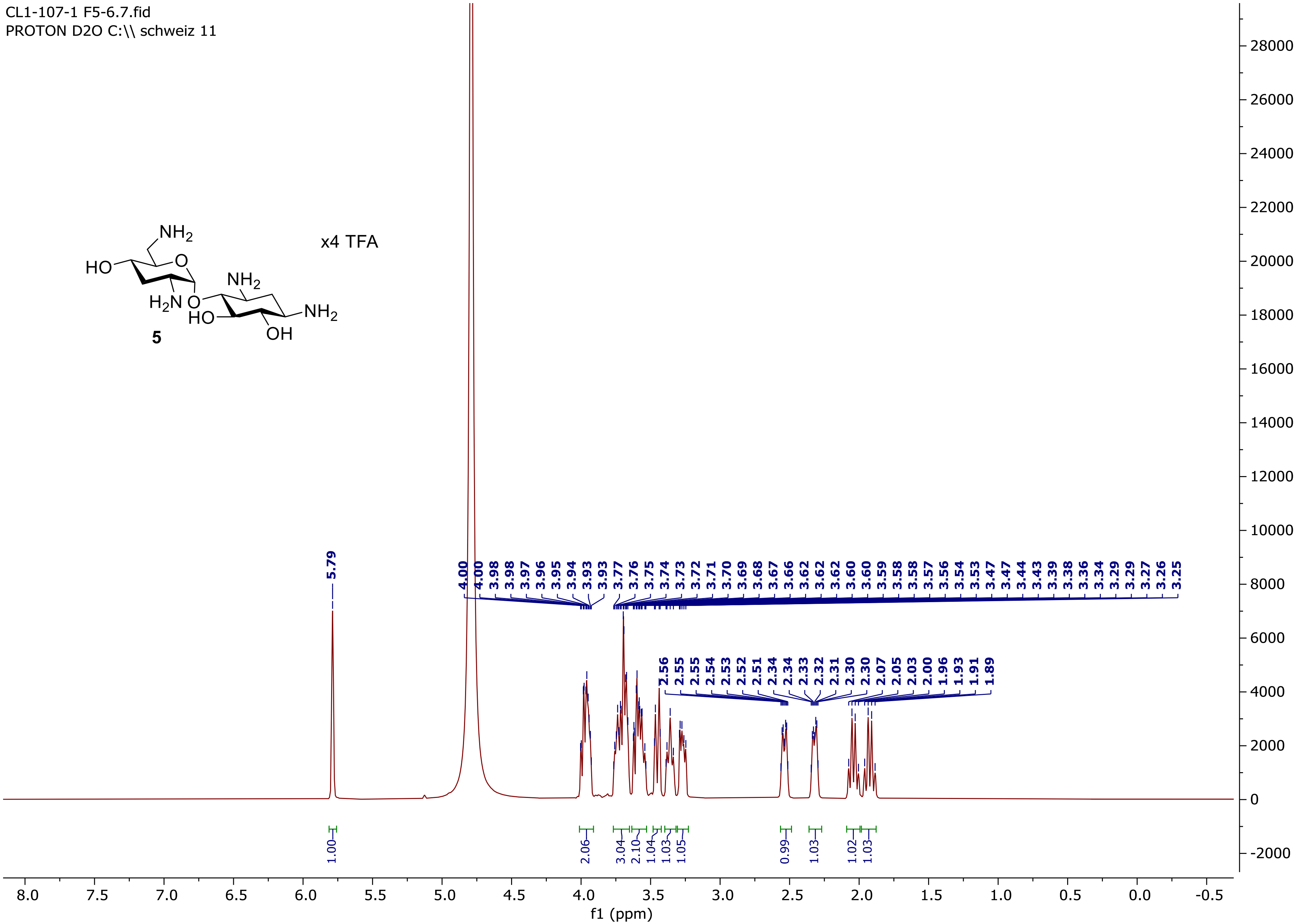

Figure S23. ^{1}H NMR spectrum for compound **5** in D_2O.

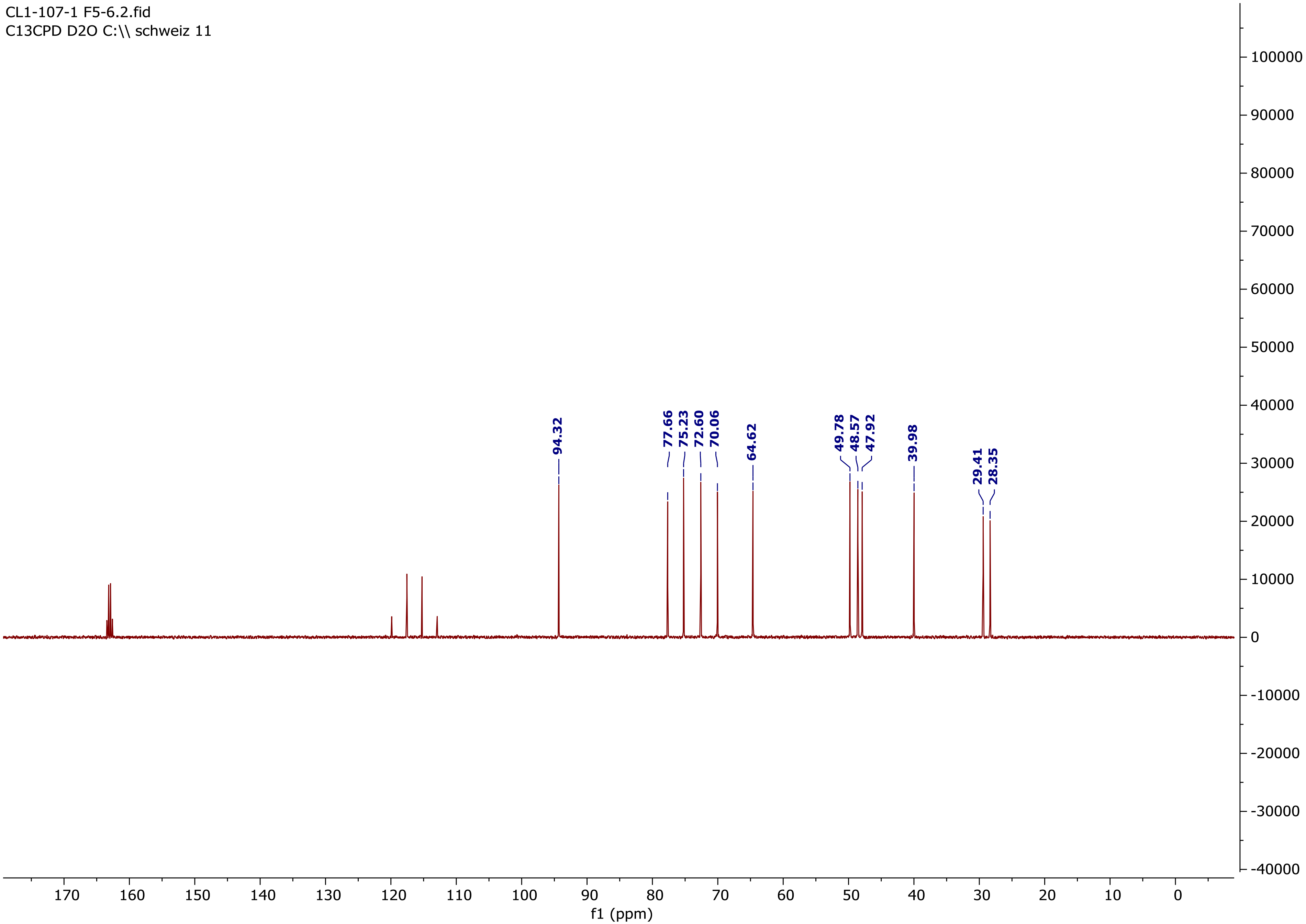

Figure S24. ^{13}C NMR spectrum for compound **5** in D_2O.

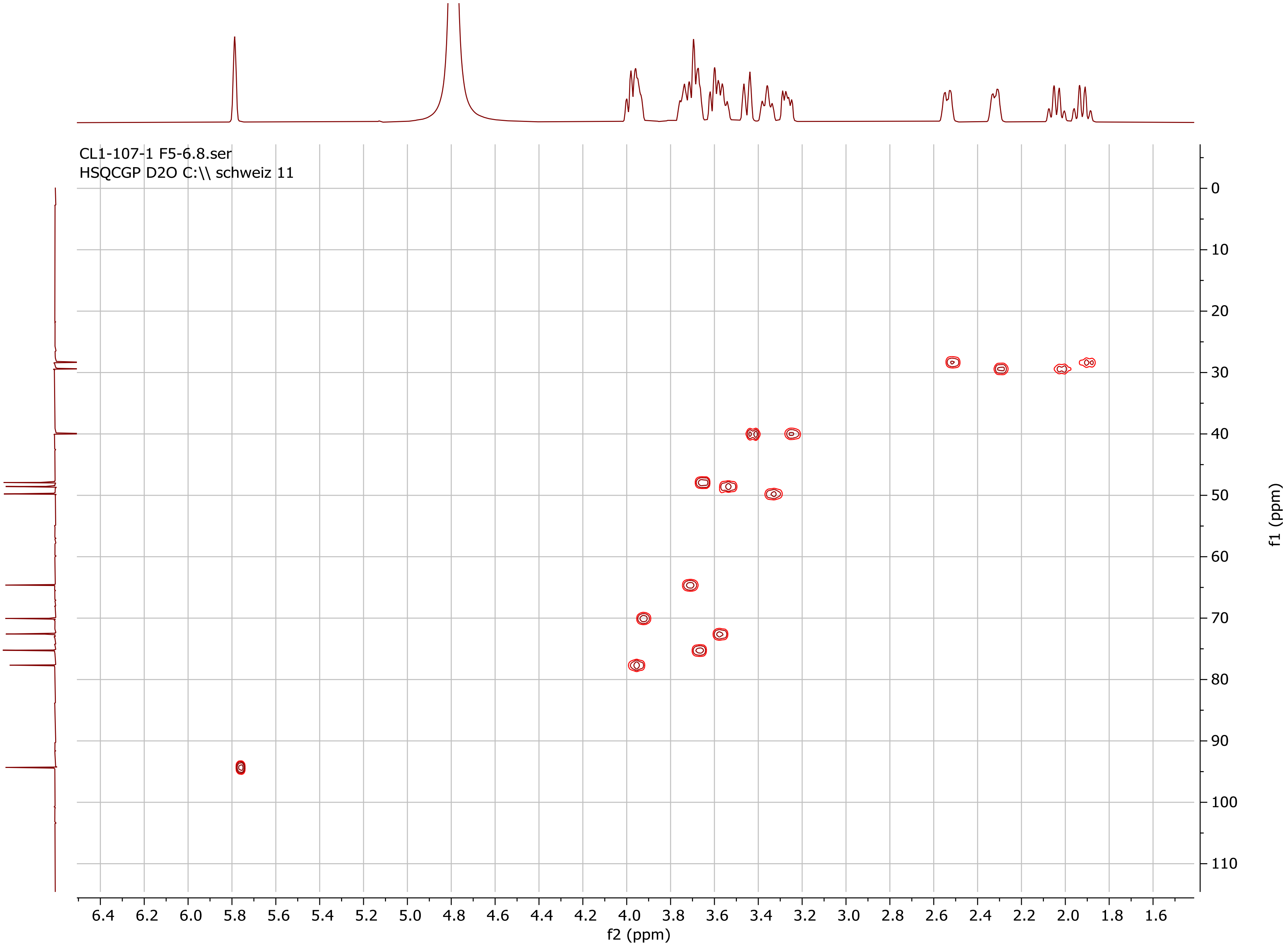

Figure S25. HSQC NMR spectrum for compound **5** in D_2O.

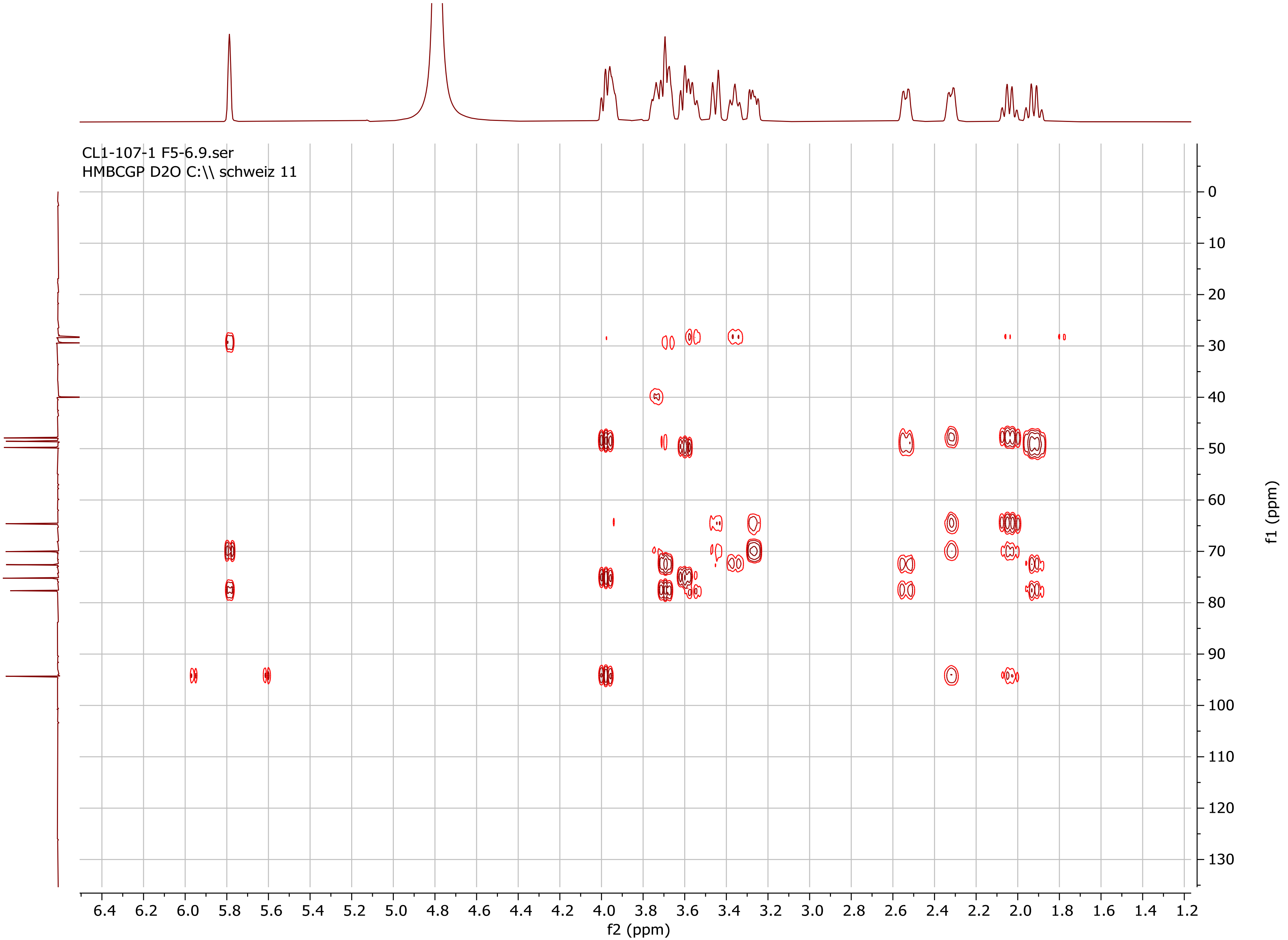

Figure S26. HMBC NMR spectrum for compound **5** in D_2O.

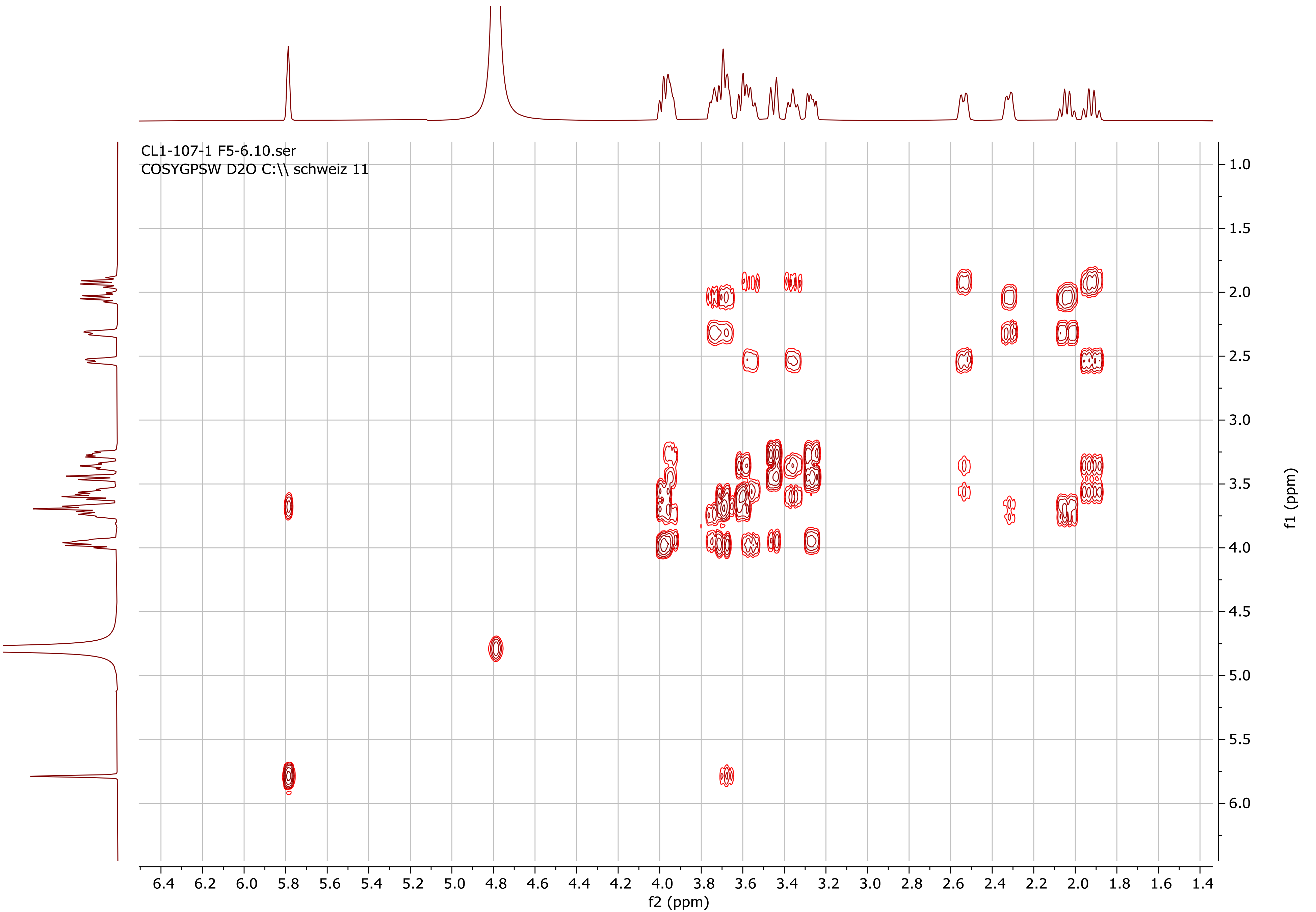

Figure S27. COSY NMR spectrum for compound **5** in D_2O.

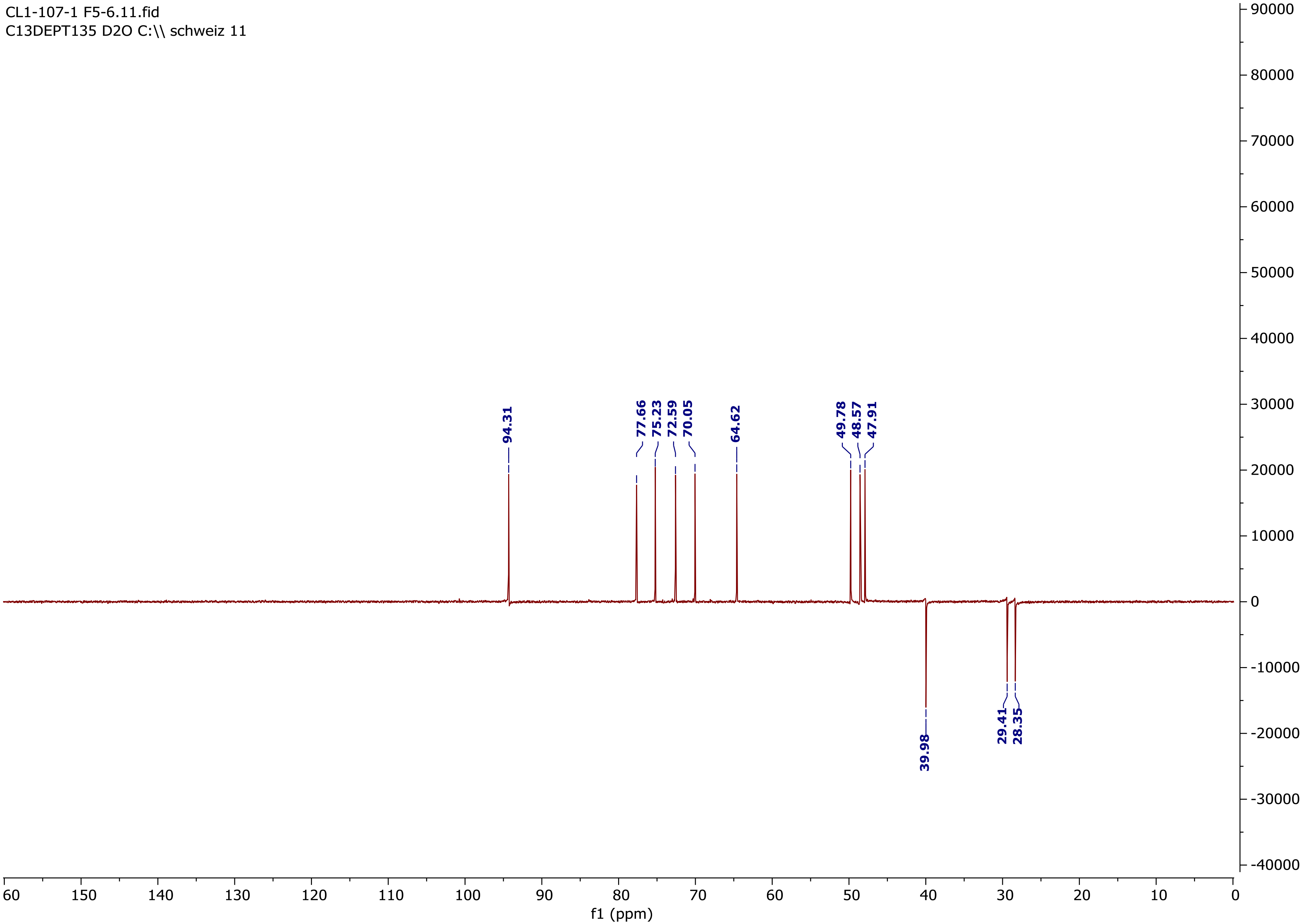

Figure S28. DEPT135 NMR spectrum for compound **5** in D_2O.

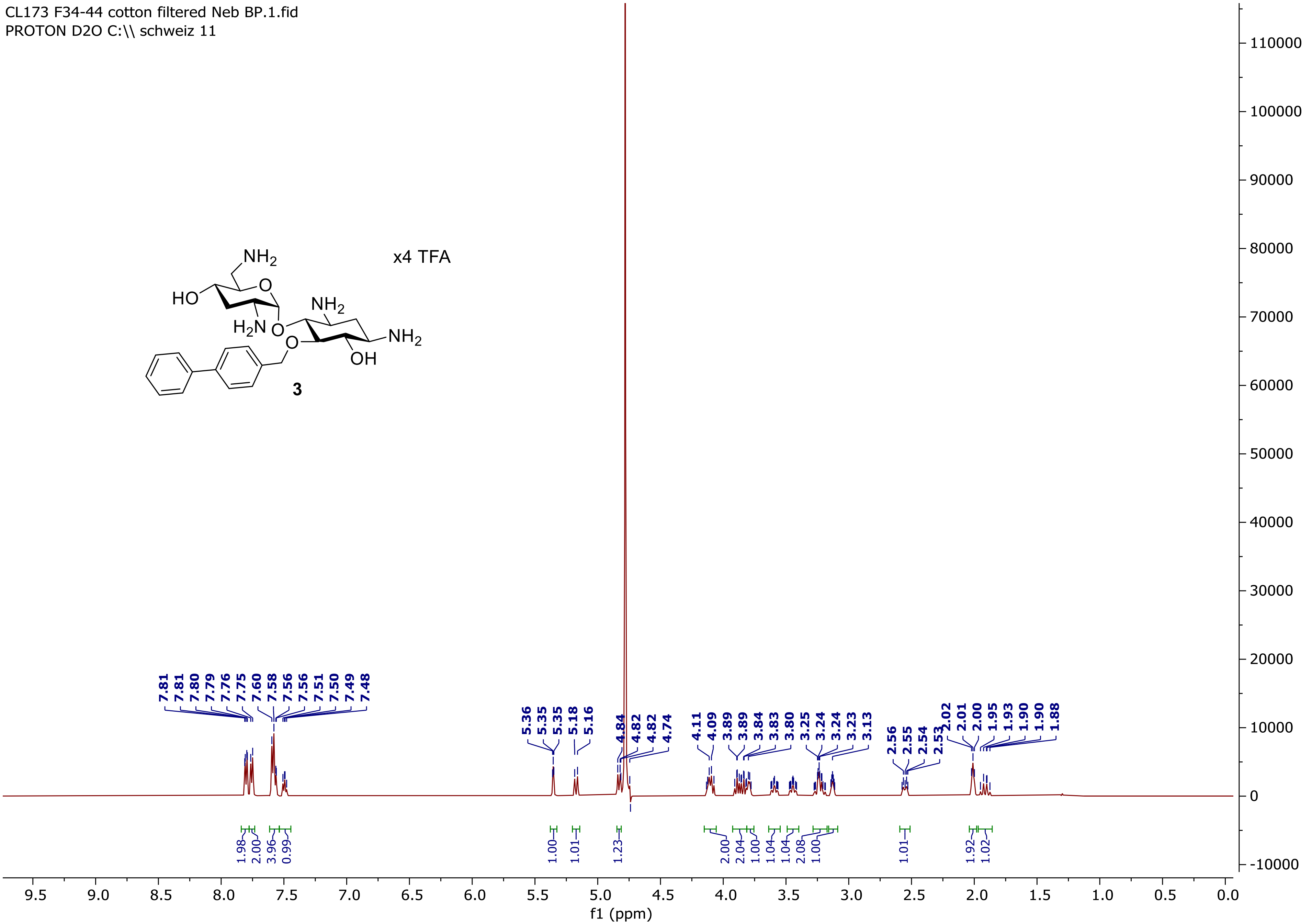

Figure S29. ^{1}H NMR spectrum for compound **3** in D_2O.

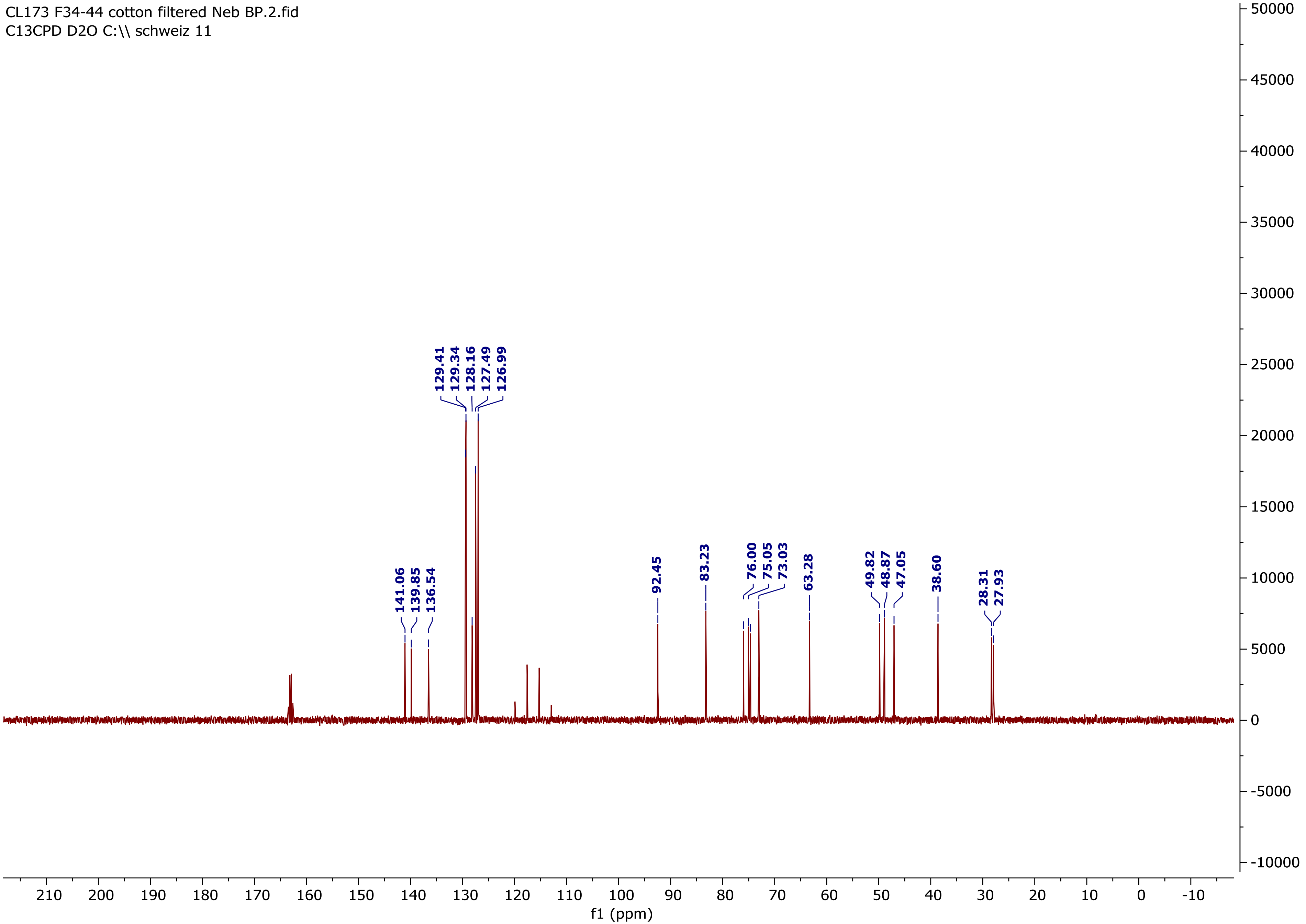

Figure S30. ^{13}C NMR spectrum for compound **3** in D_2O.

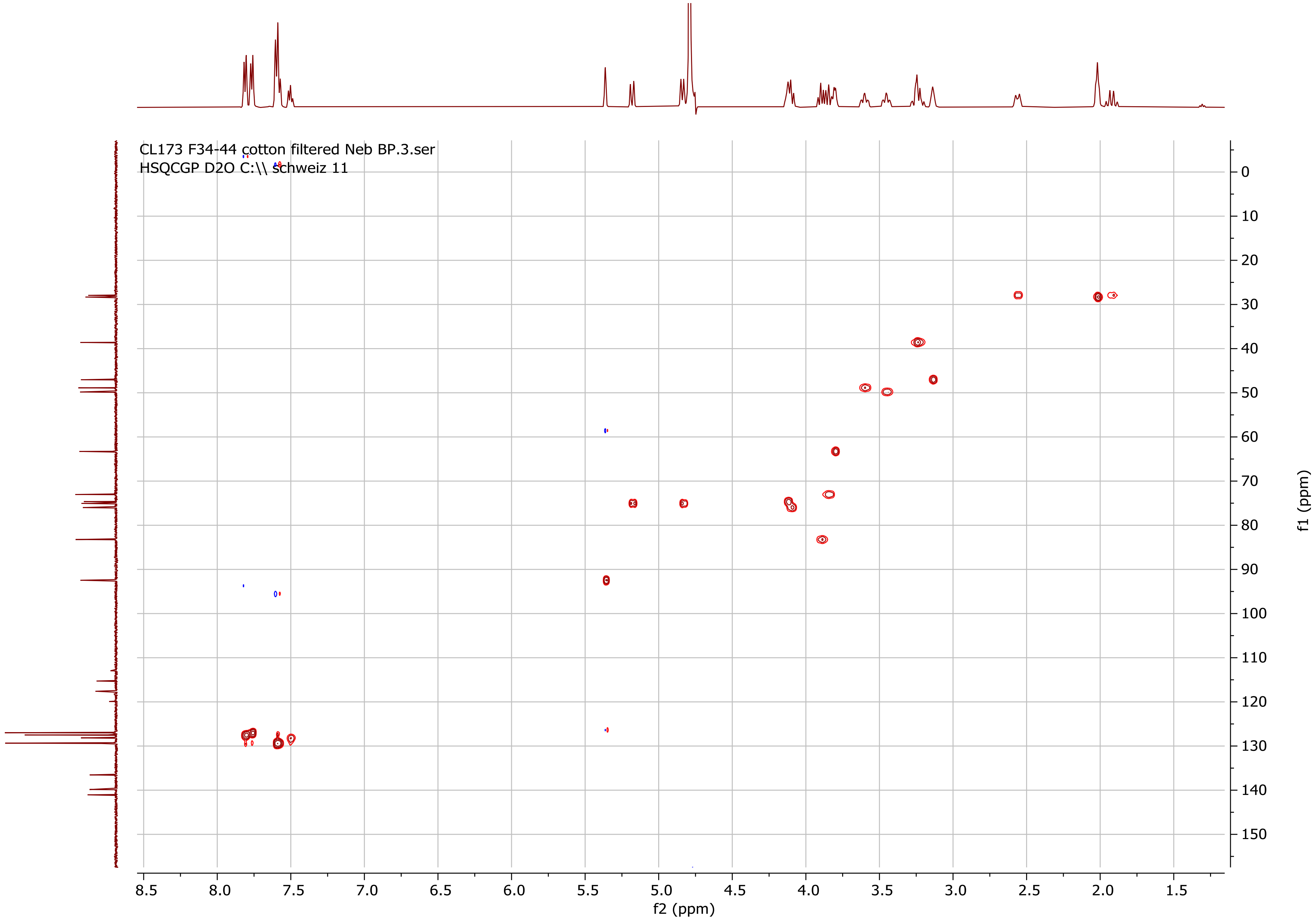

Figure S31. HSQC NMR spectrum for compound **3** in D_2O.

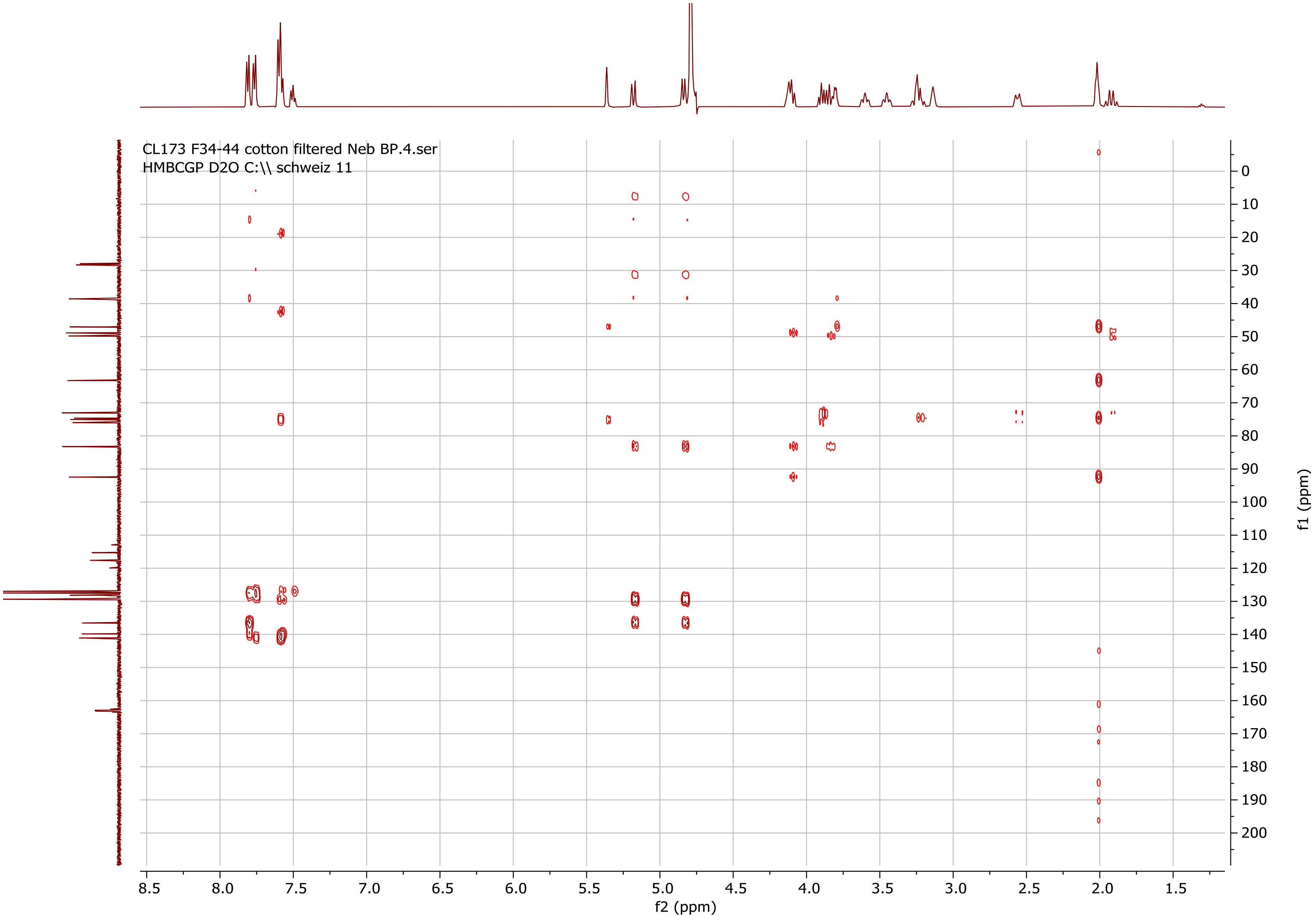

Figure S32. HMBC NMR spectrum for compound **3** in D_2O.

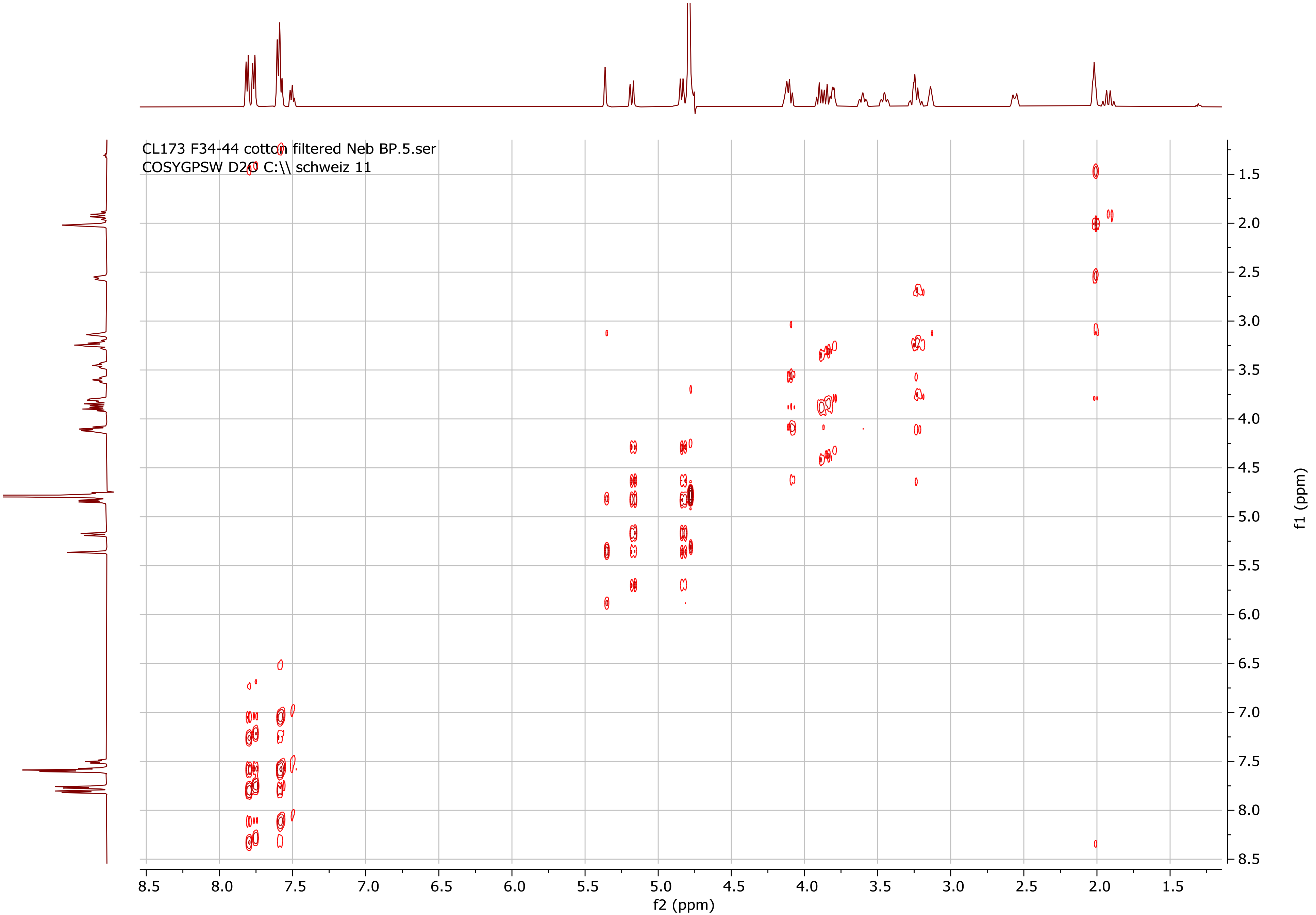

Figure S33. COSY NMR spectrum for compound **3** in D_2O.

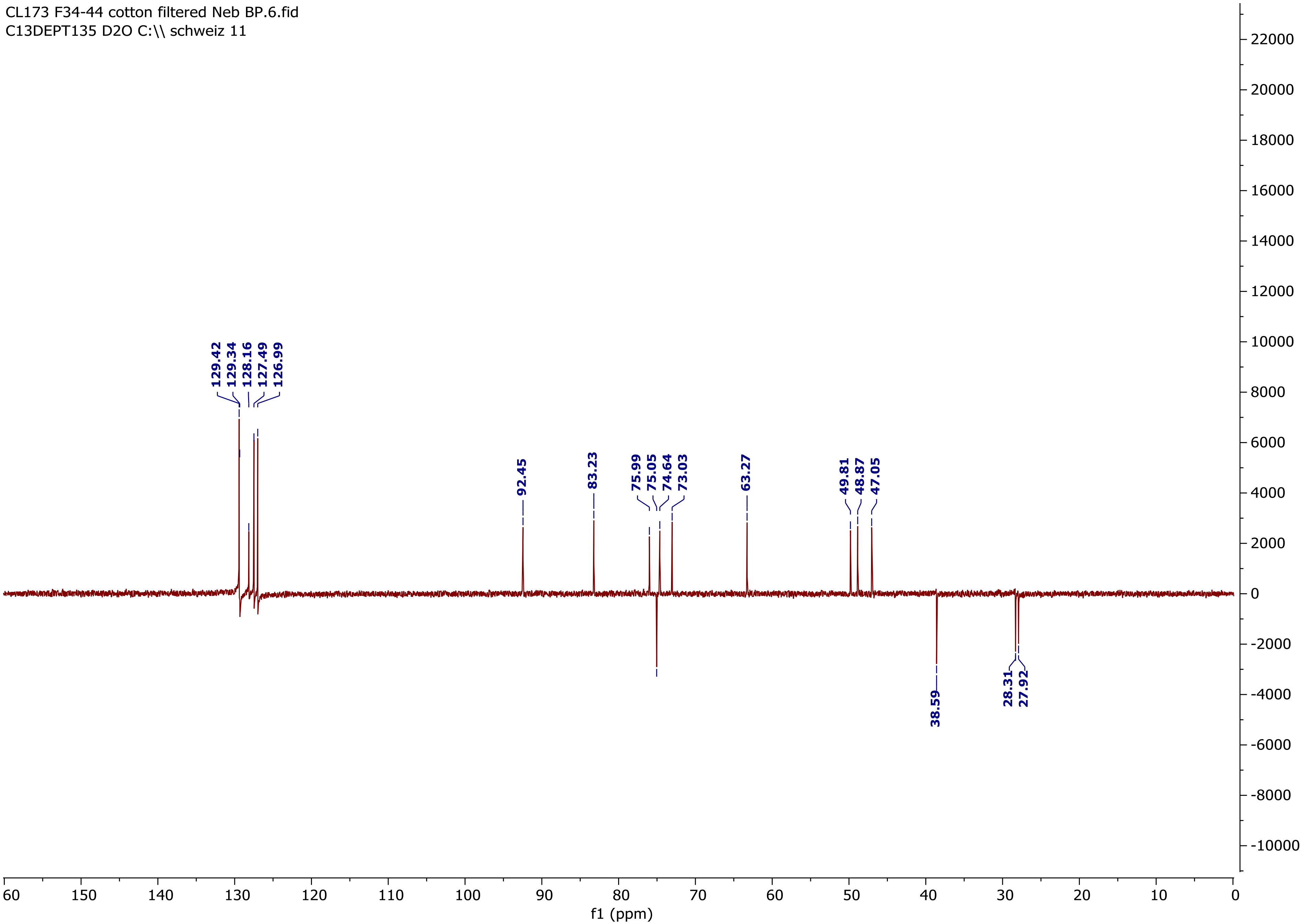

Figure S34. DEPT135 NMR spectrum for compound **3** in D_2O.

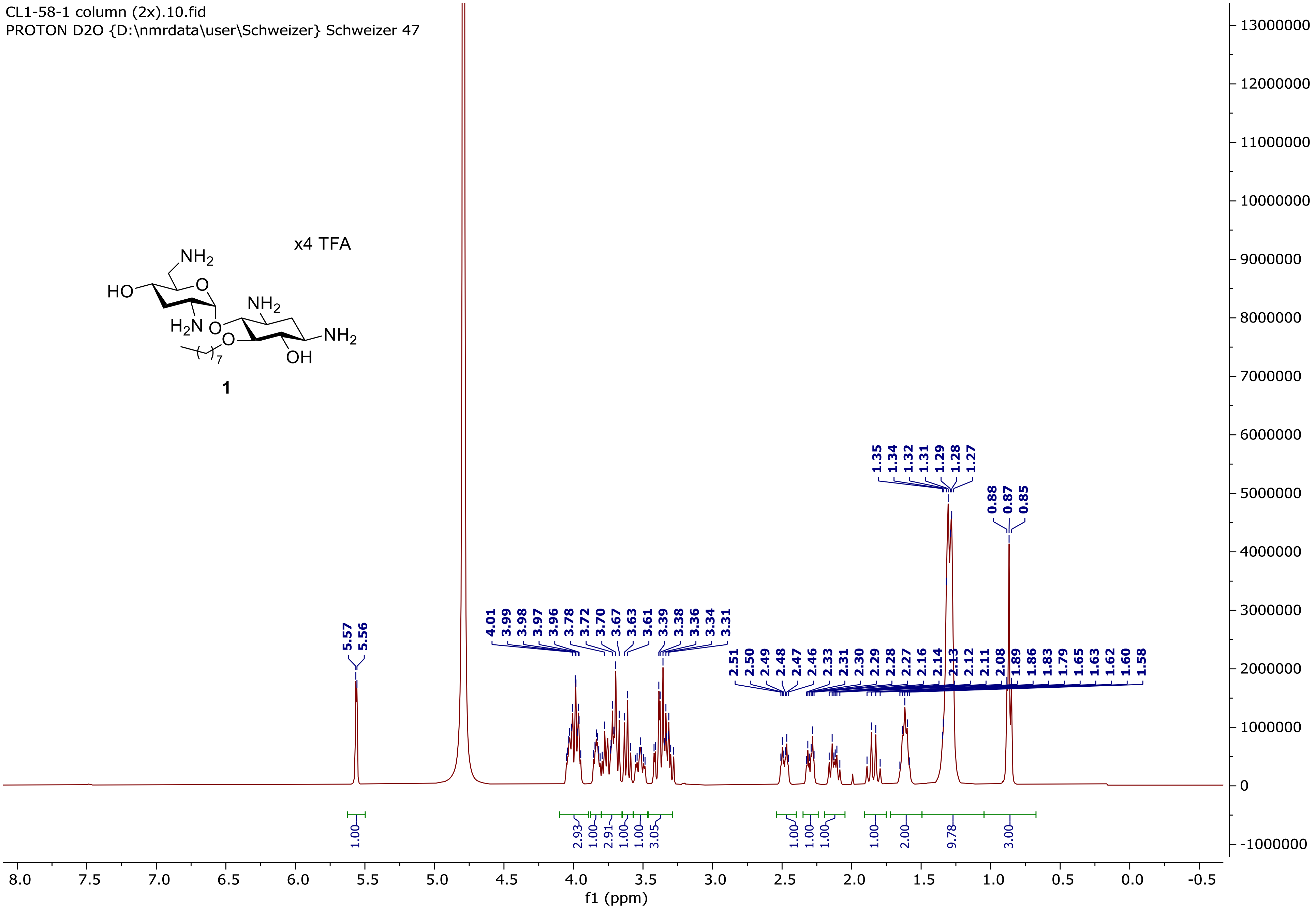

Figure S35. ^{1}H NMR spectrum for compound **1** in D_2O.

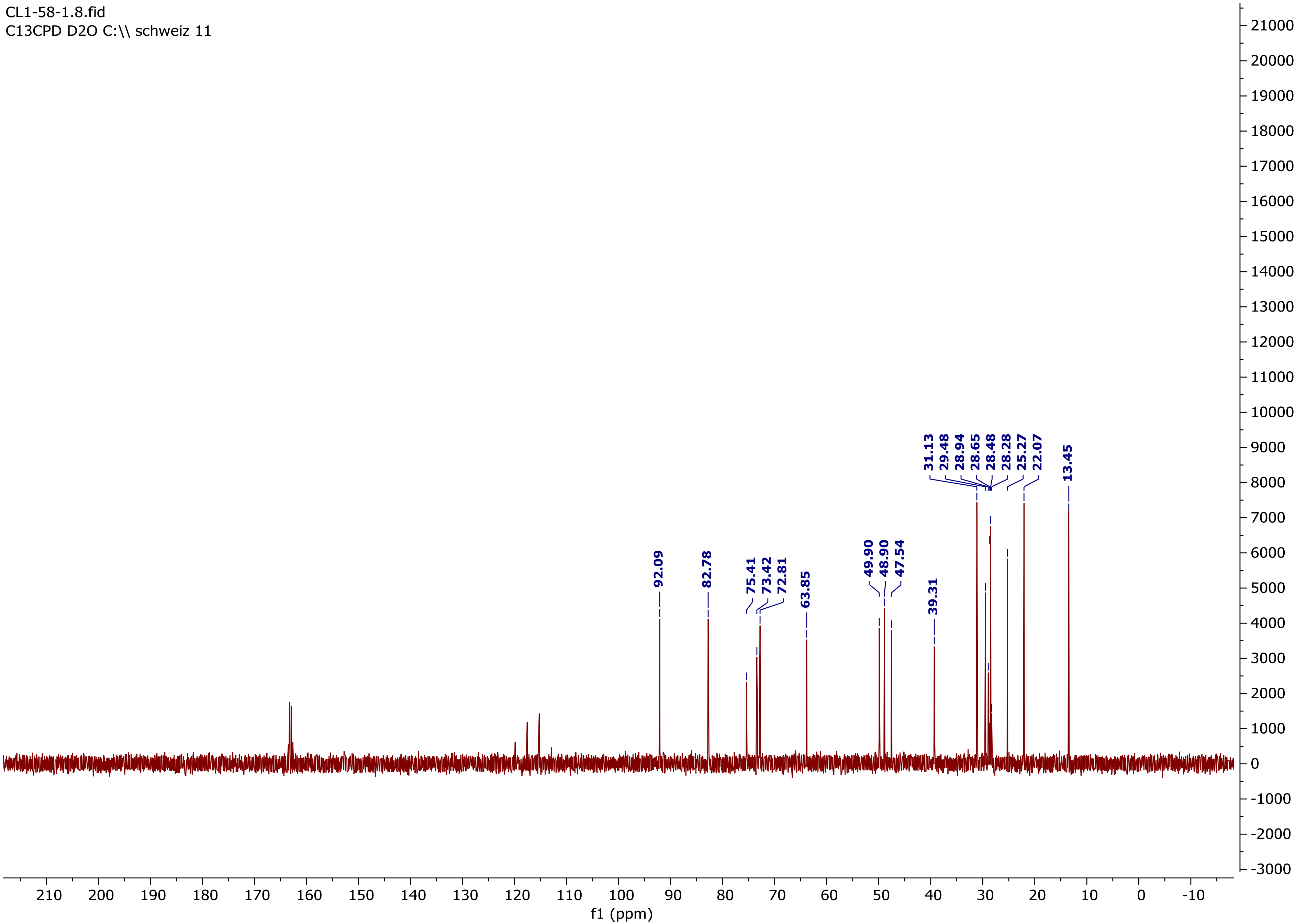

Figure S36. ^{13}C NMR spectrum for compound **1** in D_2O.

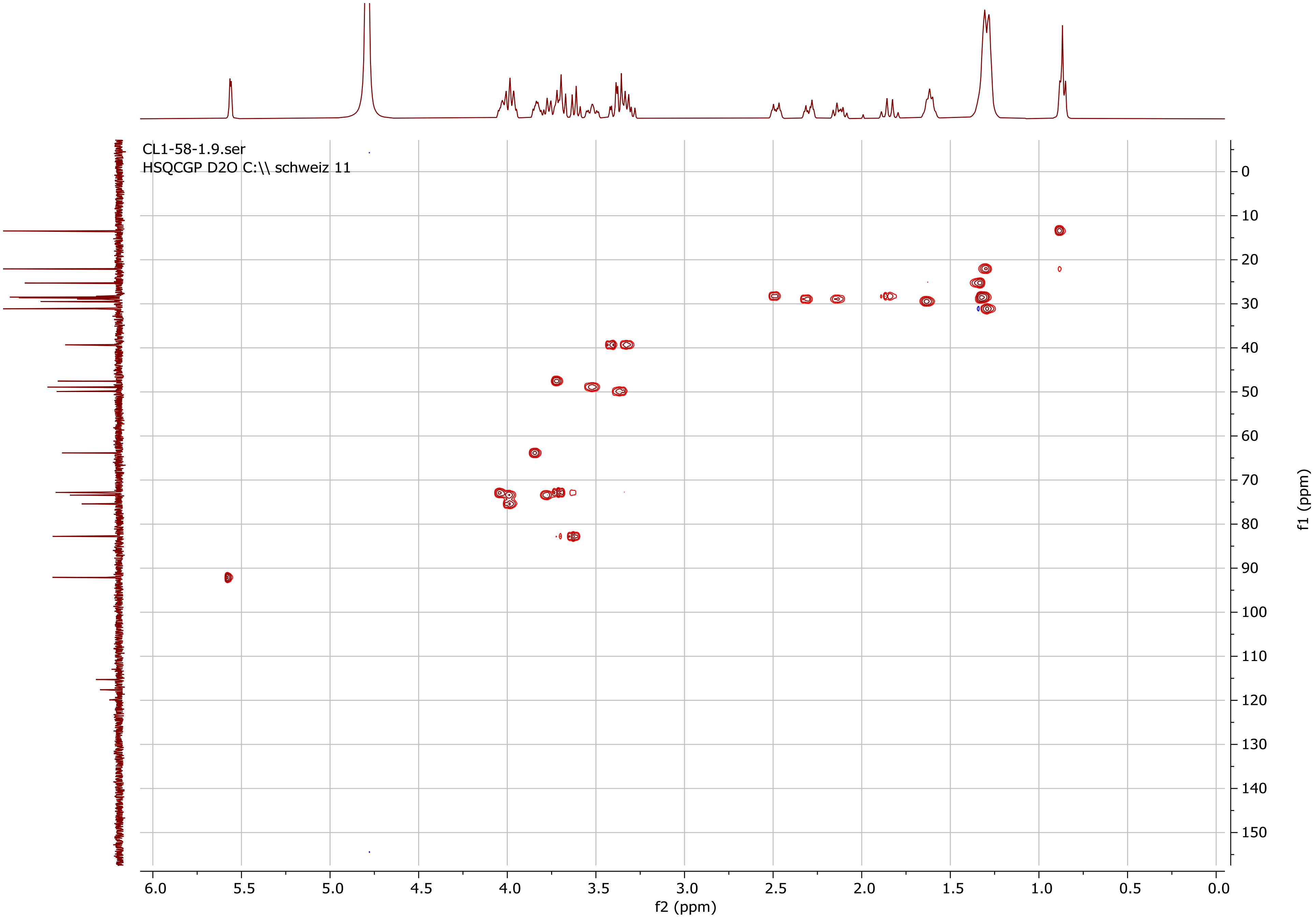

Figure S37. HSQC NMR spectrum for compound **1** in D_2O.

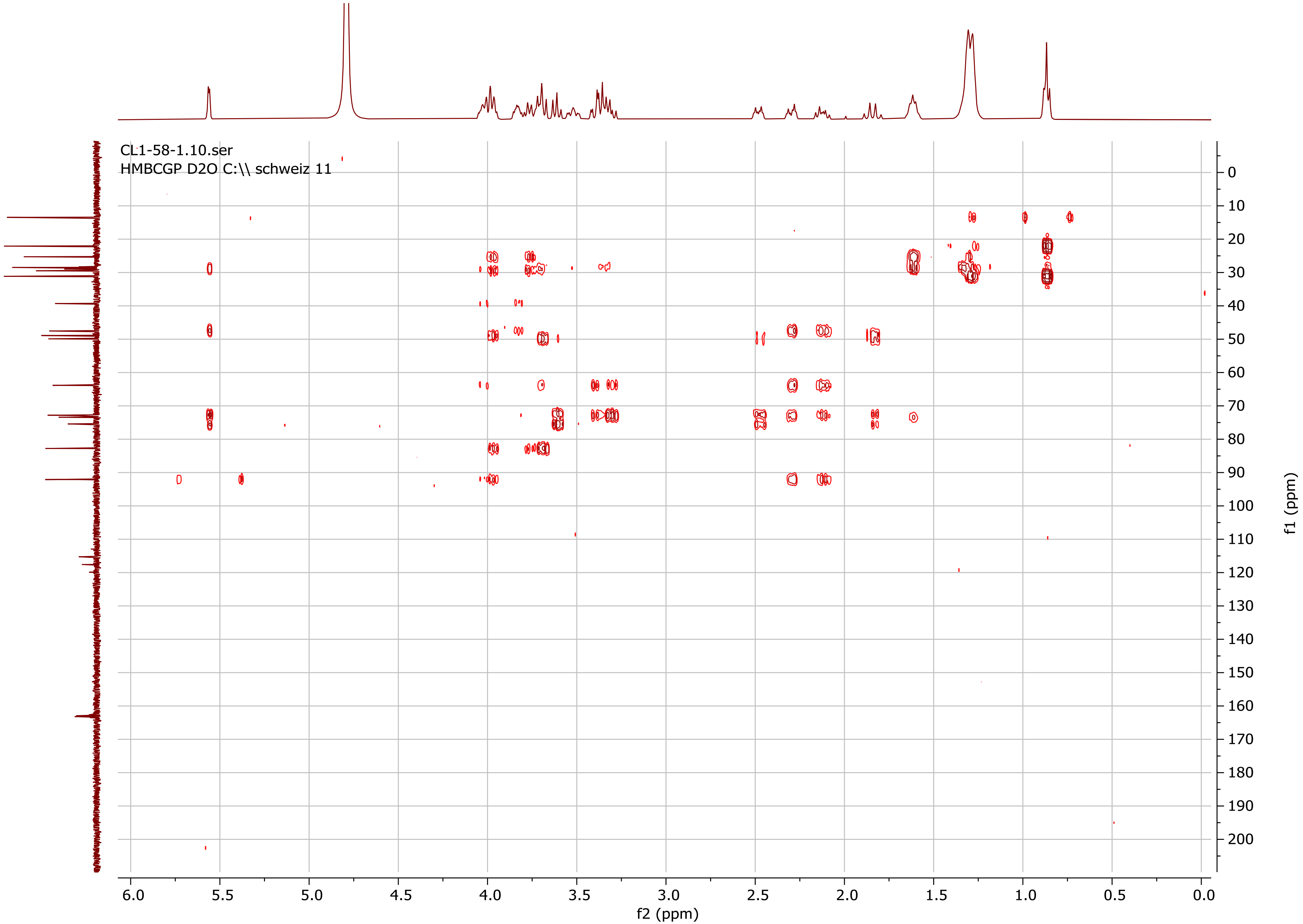

Figure S38. HMBC NMR spectrum for compound **1** in D_2O.

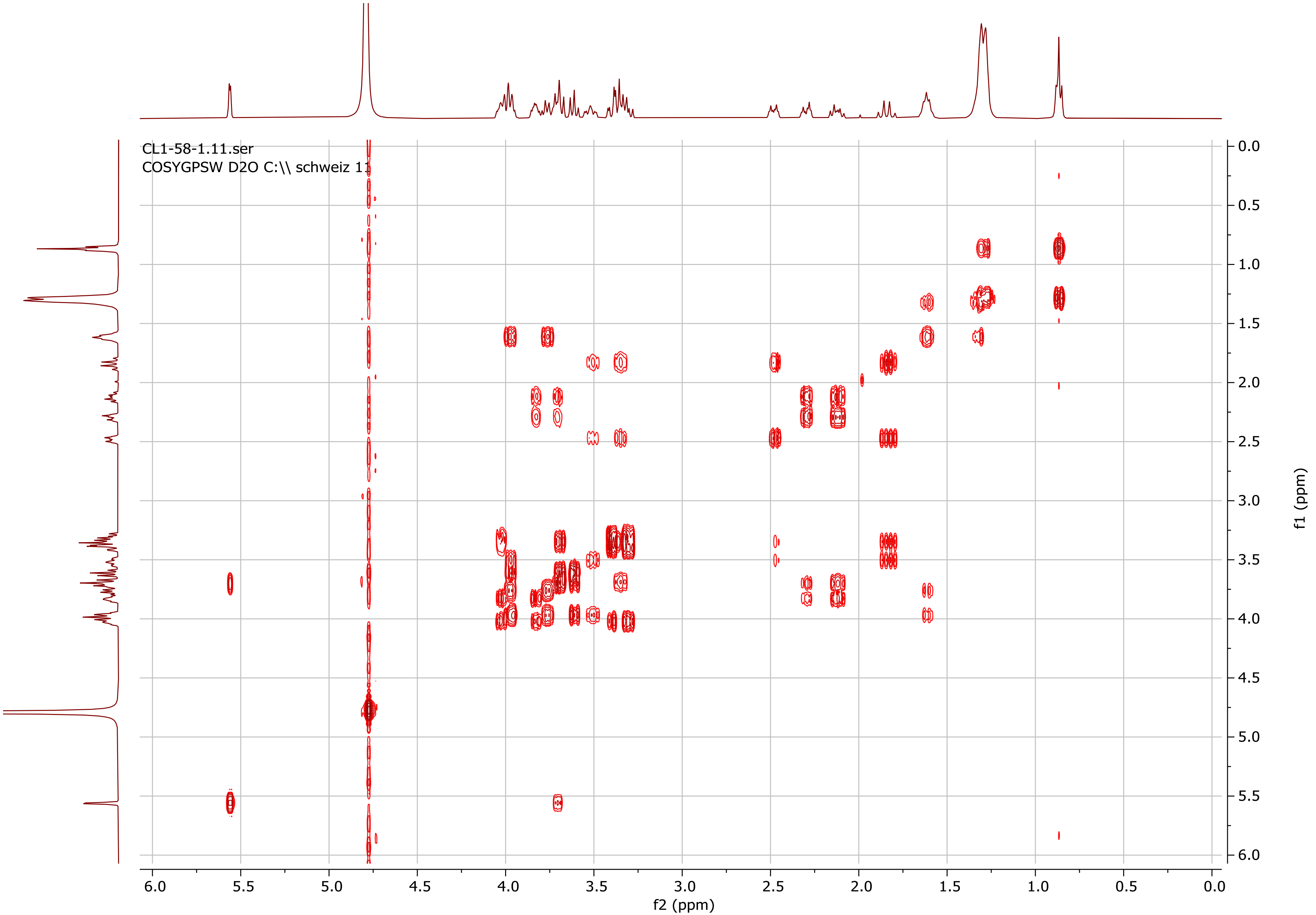

Figure S39. COSY NMR spectrum for compound **1** in D_2O.

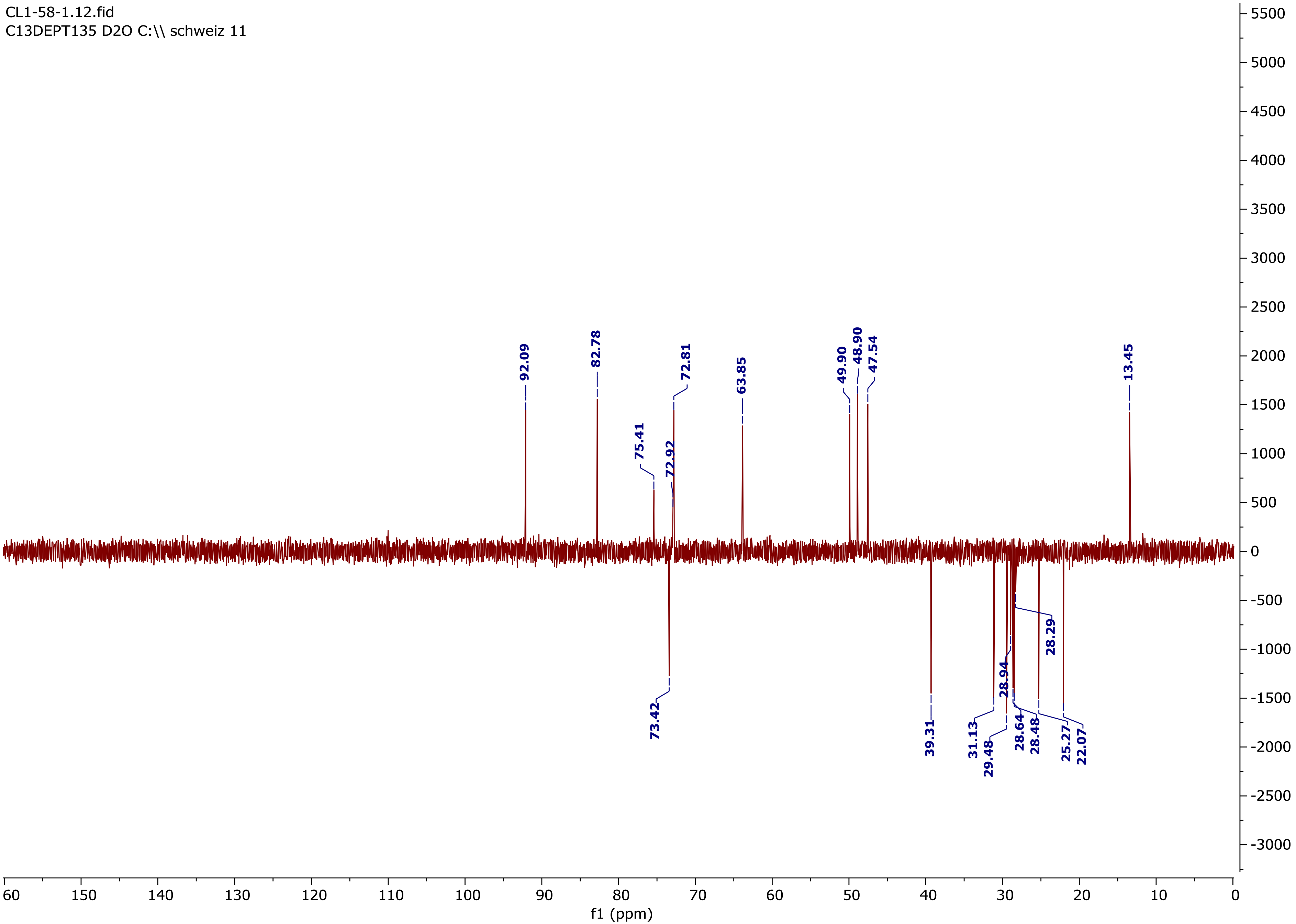

Figure S40. DEPT135 NMR spectrum for compound **1** in D_2O.

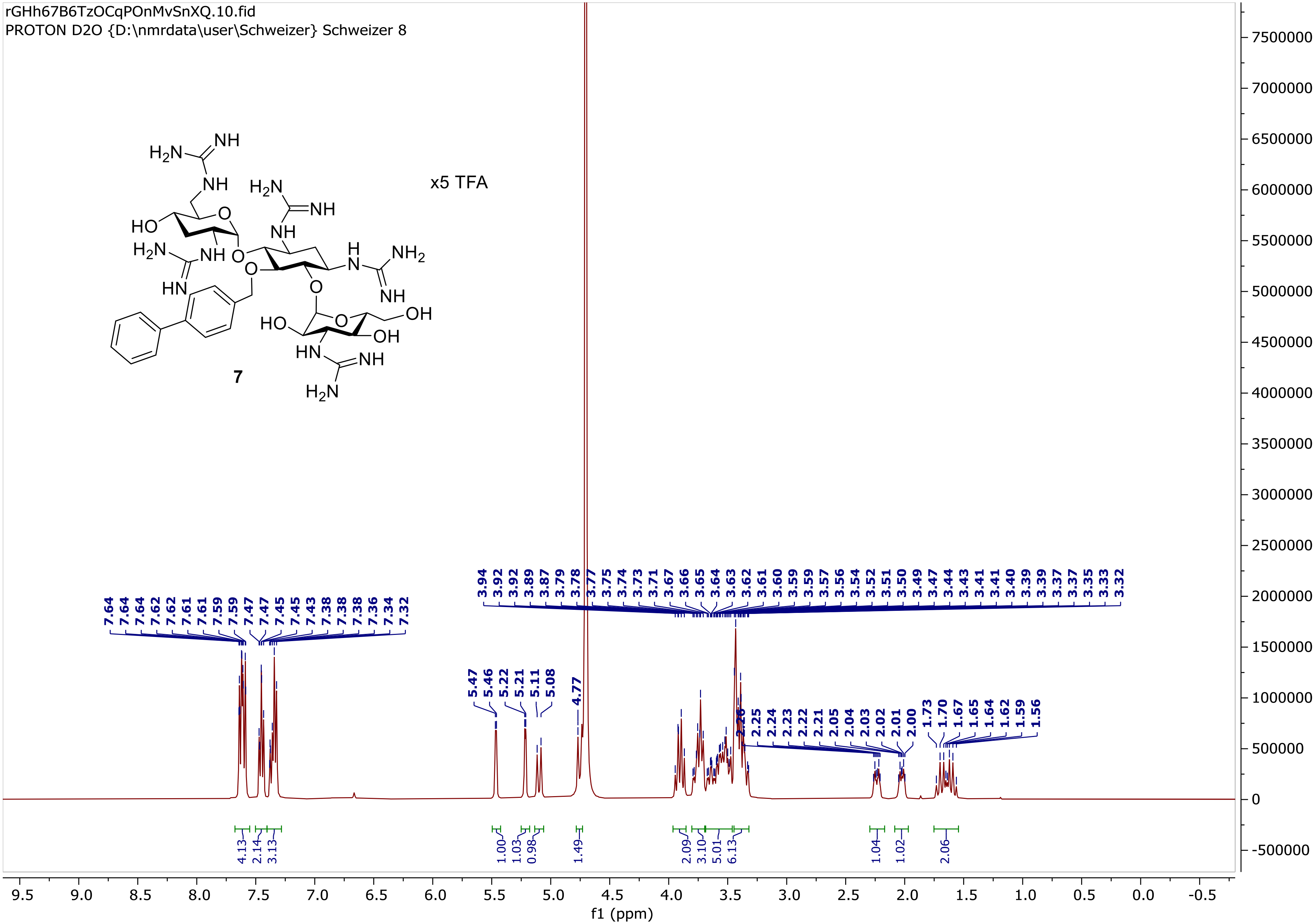

Figure S41. ^{1}H NMR spectrum for compound **7** in D_2O.

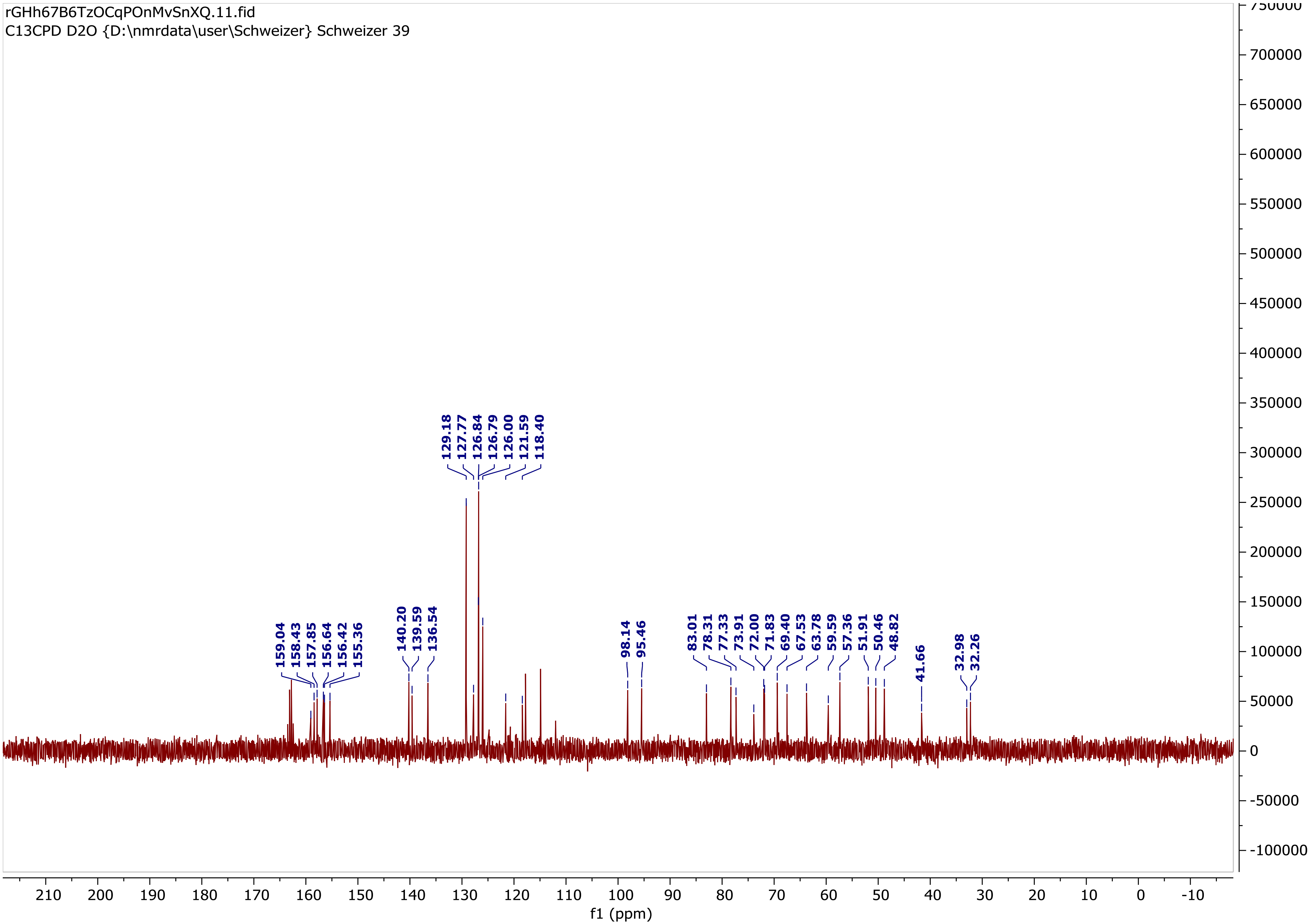

Figure S42. ^{13}C NMR spectrum for compound **7** in D_2O.

CL1-134 BocNeb F0.1.fid
PROTON MeOD C:\\ schweiz 1

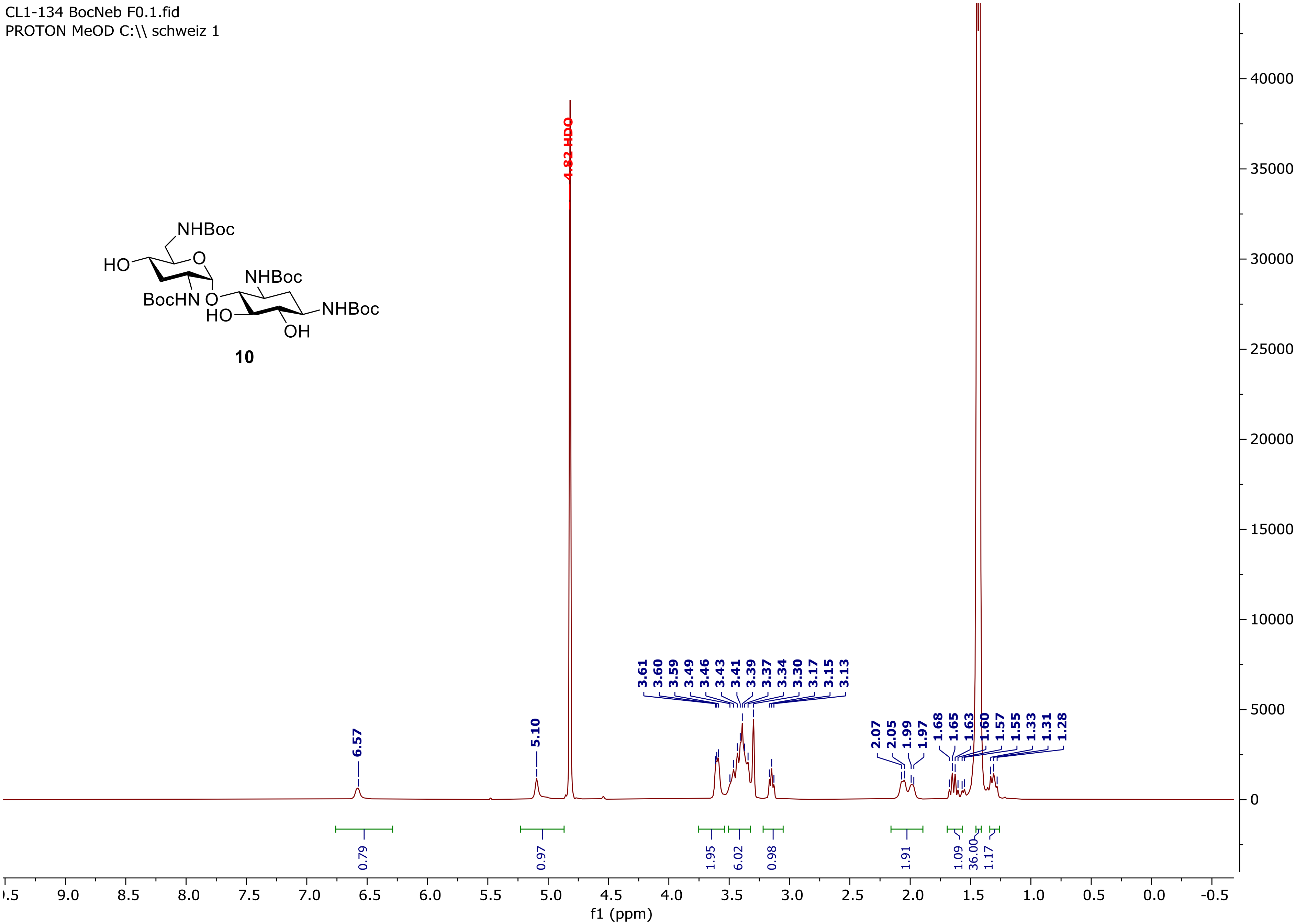

Figure S43. ^{1}H NMR spectrum for compound **10** in MeOD.

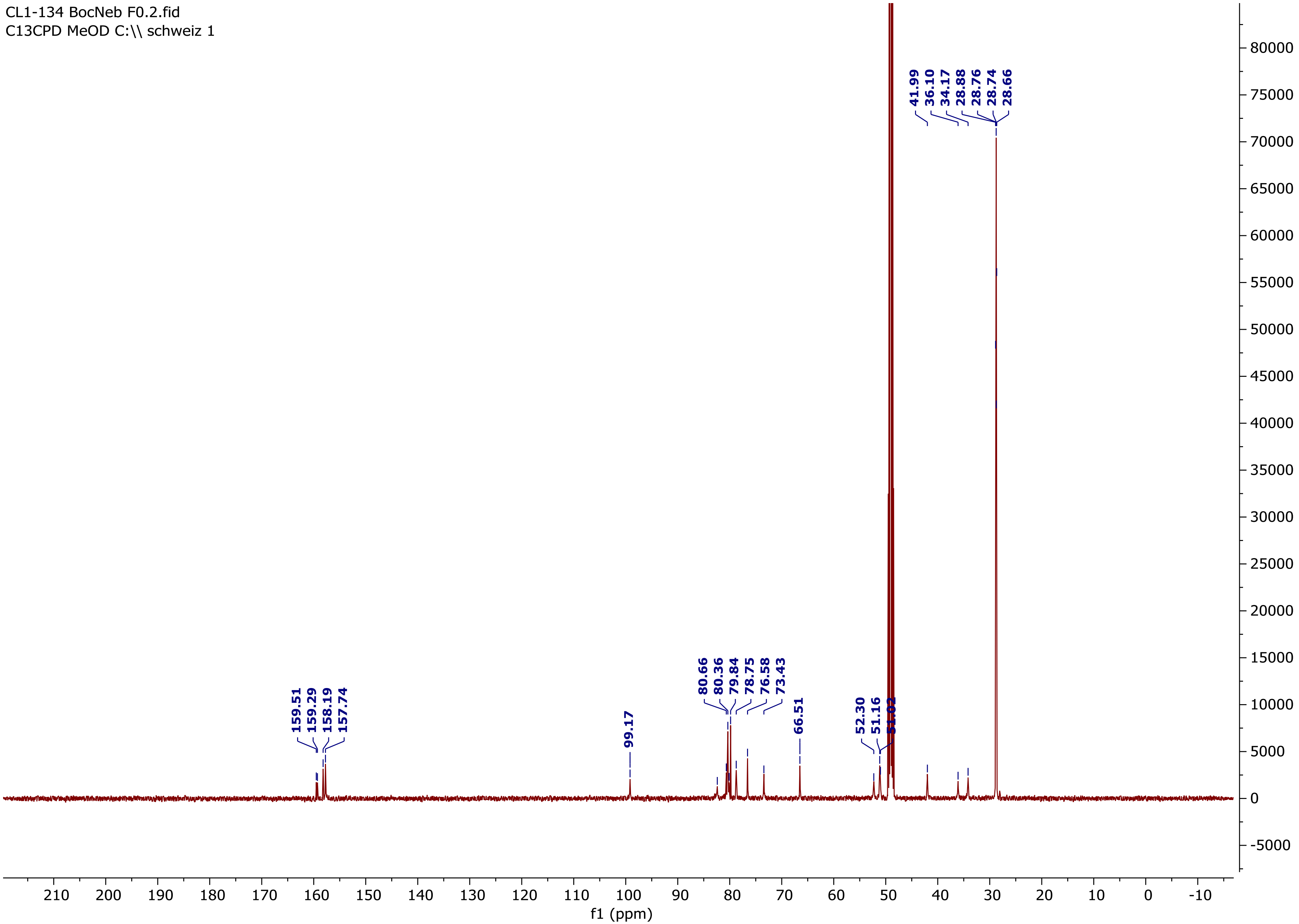

Figure S44. ^{13}C NMR spectrum for compound **10** in MeOD.

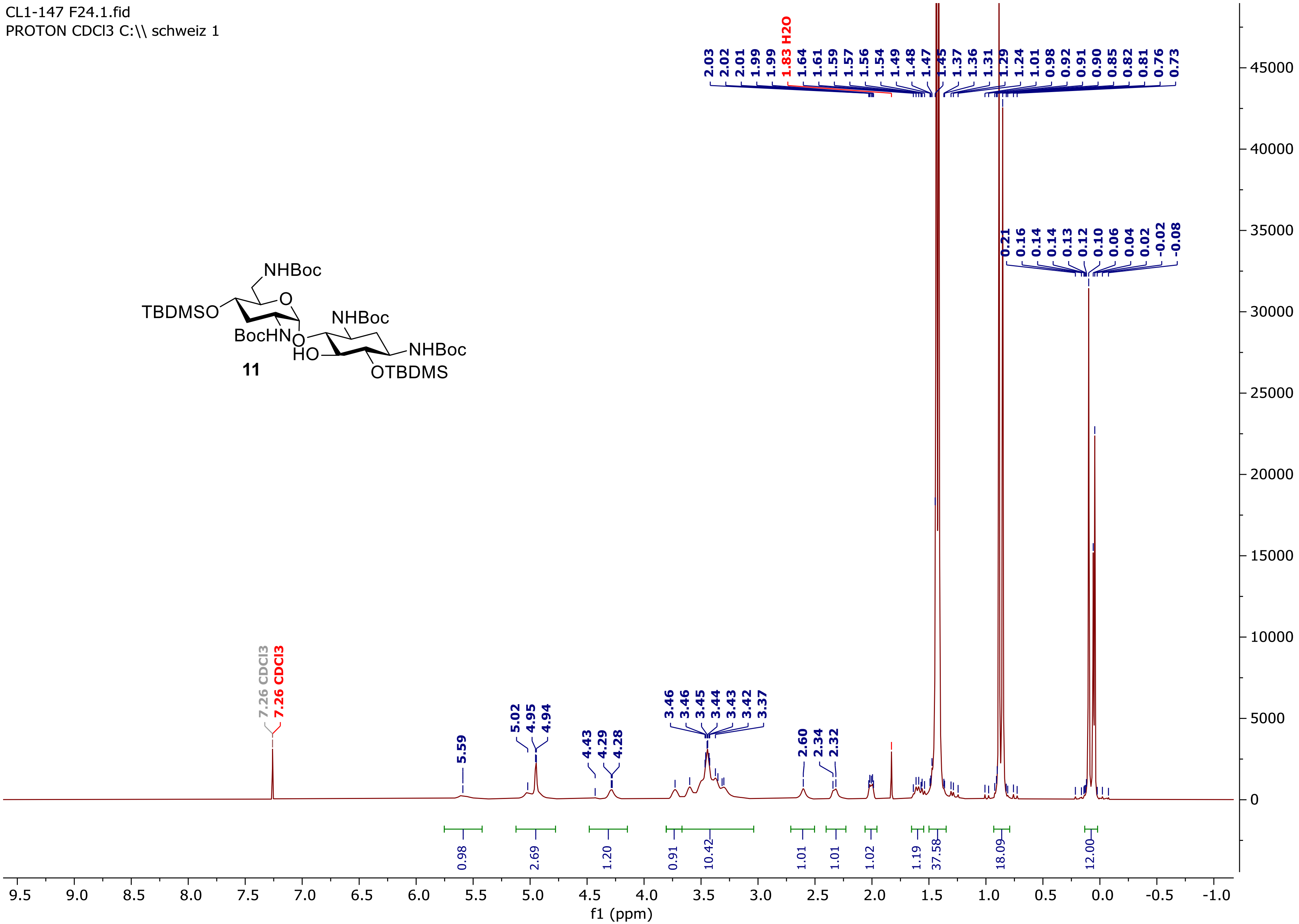

Figure S45. ¹H NMR spectrum for compound **11** in $CDCl_3$.

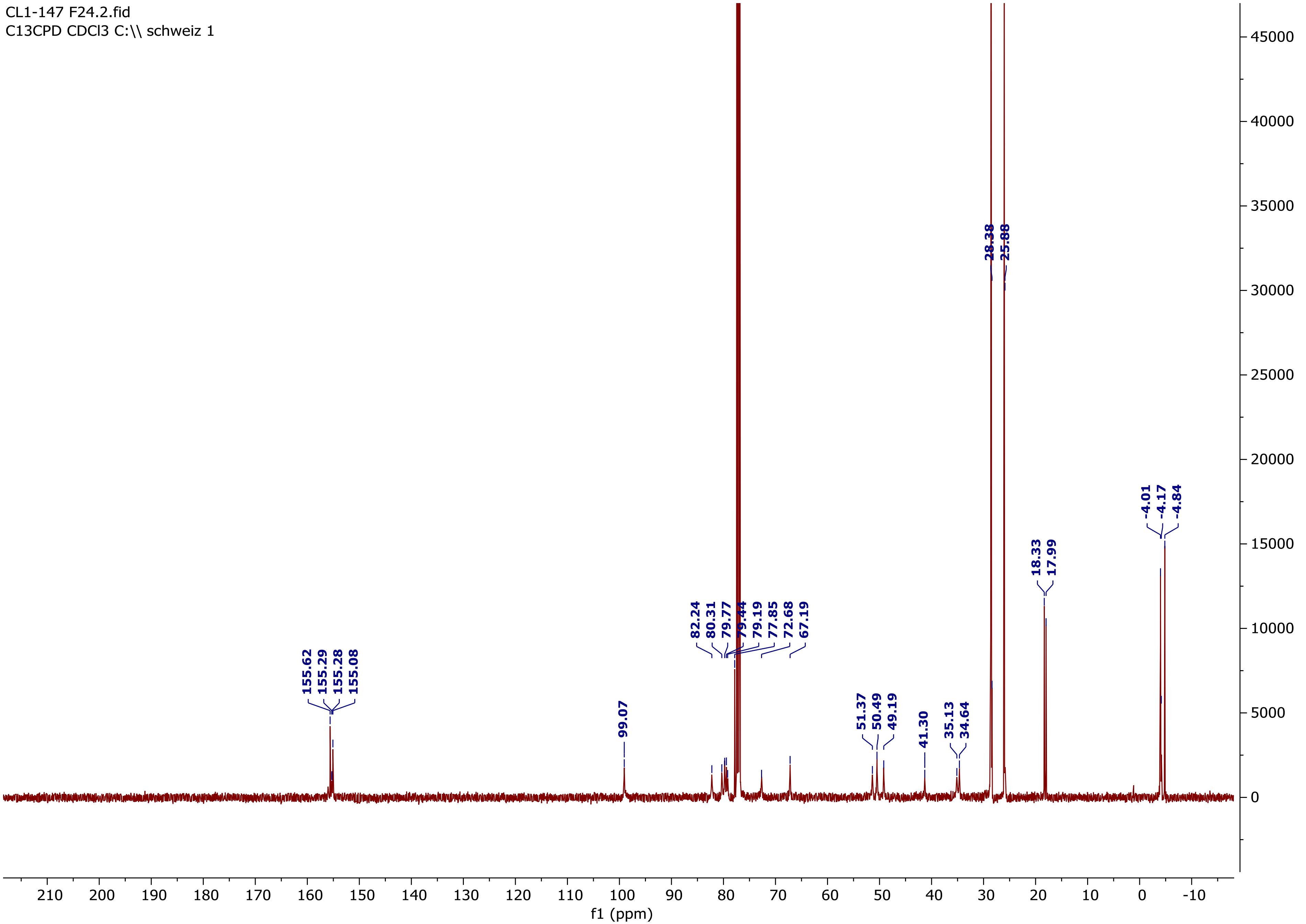

Figure S46. ^{13}C NMR spectrum for compound **11** in $CDCl_3$.

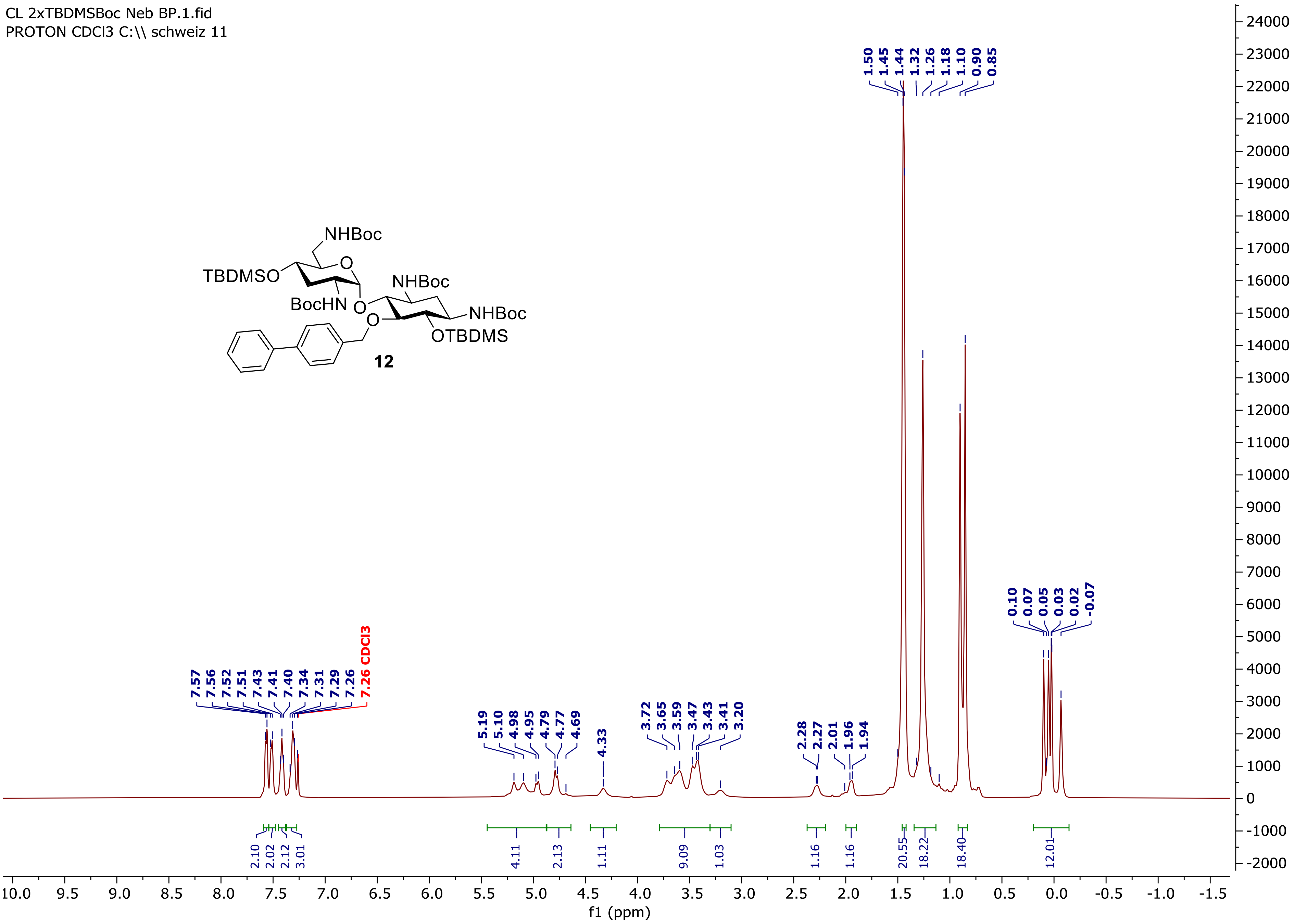

Figure S47. ^{1}H NMR spectrum for compound **12** in $CDCl_3$.

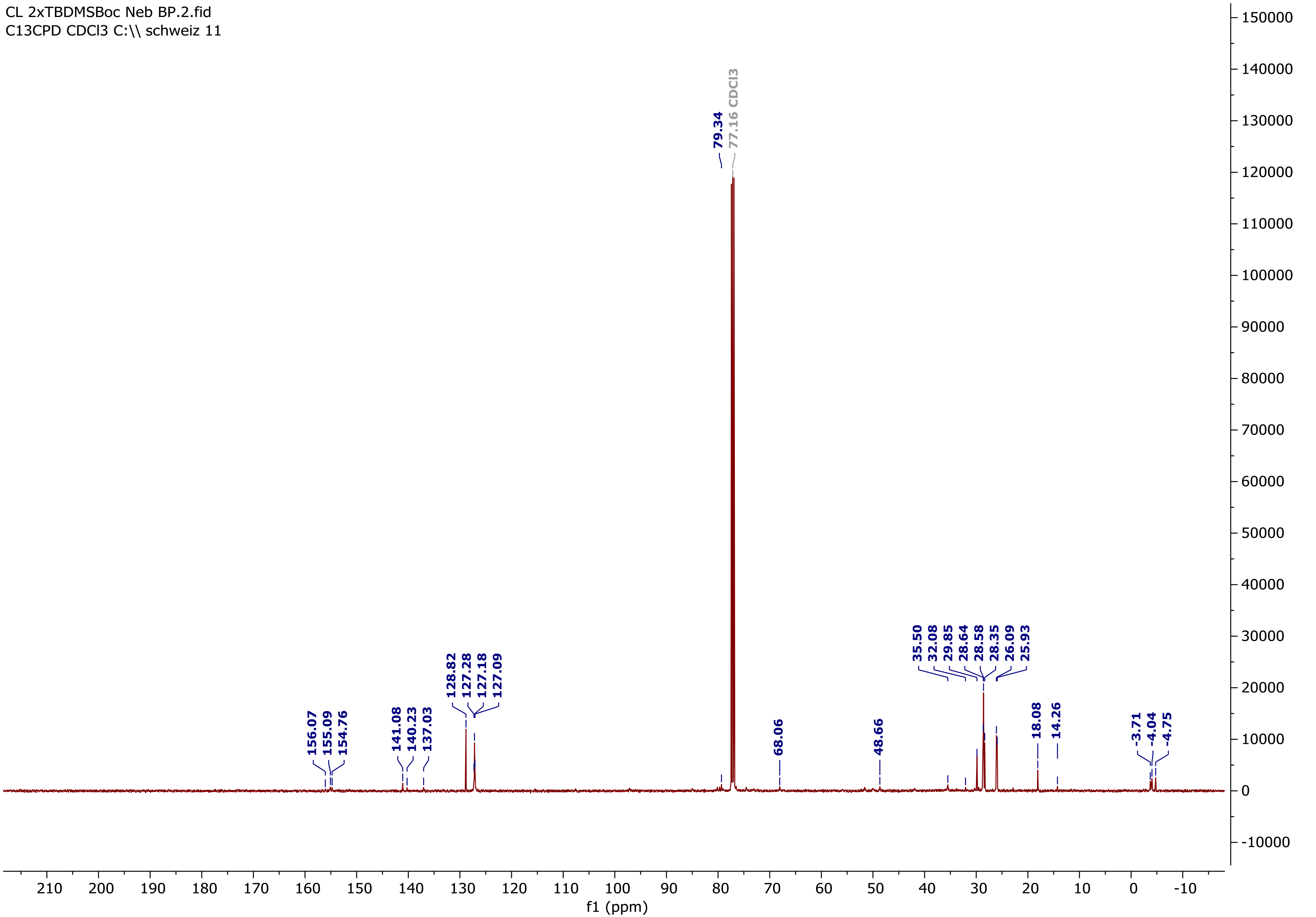

Figure S48. ^{13}C NMR spectrum for compound **12** in $CDCl_3$.

CL1-169 F18 Boc Neb BP.1.fid
PROTON CDCl3 C:\\ schweiz 11

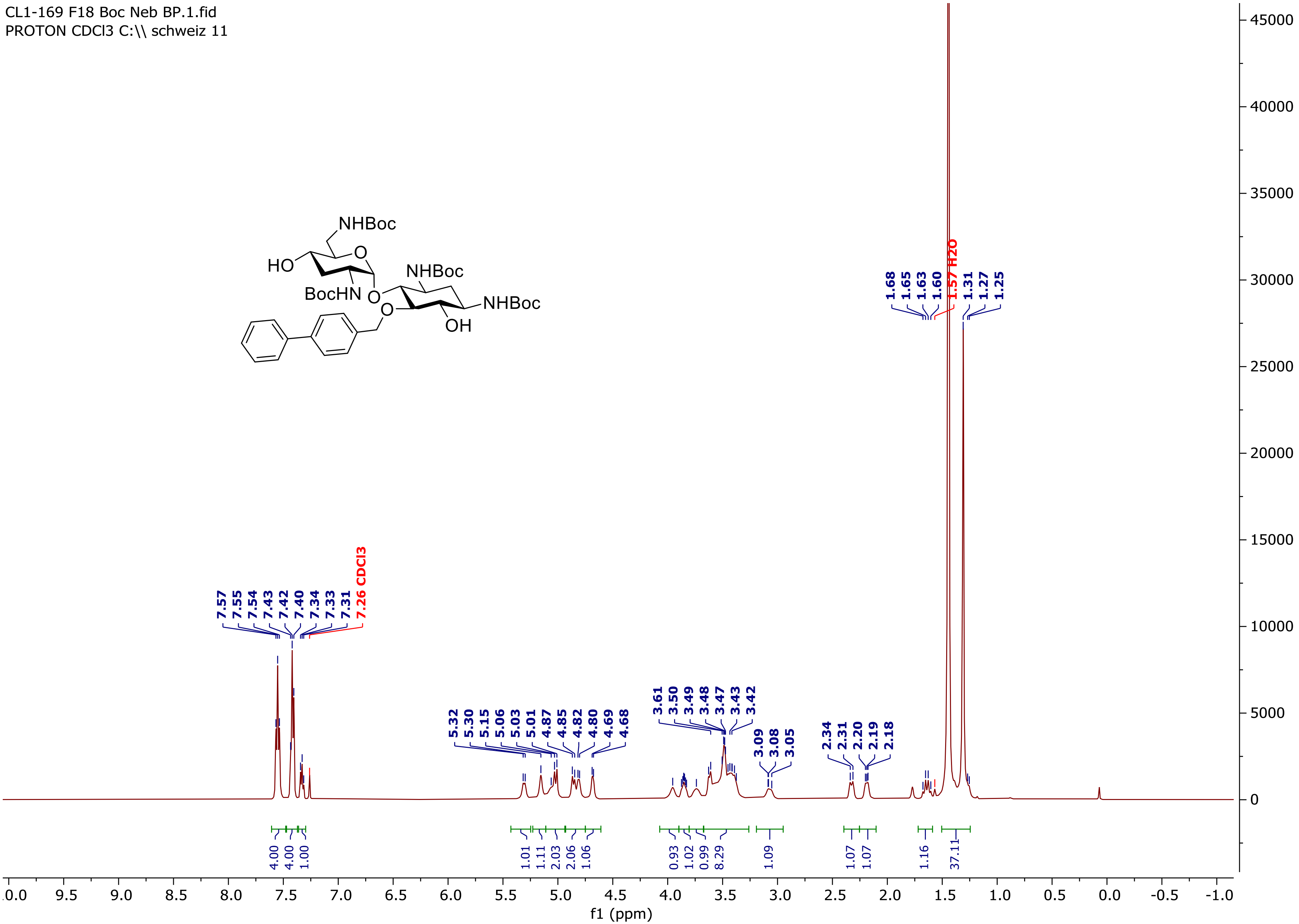

Figure S49. ^{1}H NMR spectrum for 5-O-(methylenebiphenyl)-1,3,2′,6′-tetra-N-Boc-Nebramine in $CDCl_3$.

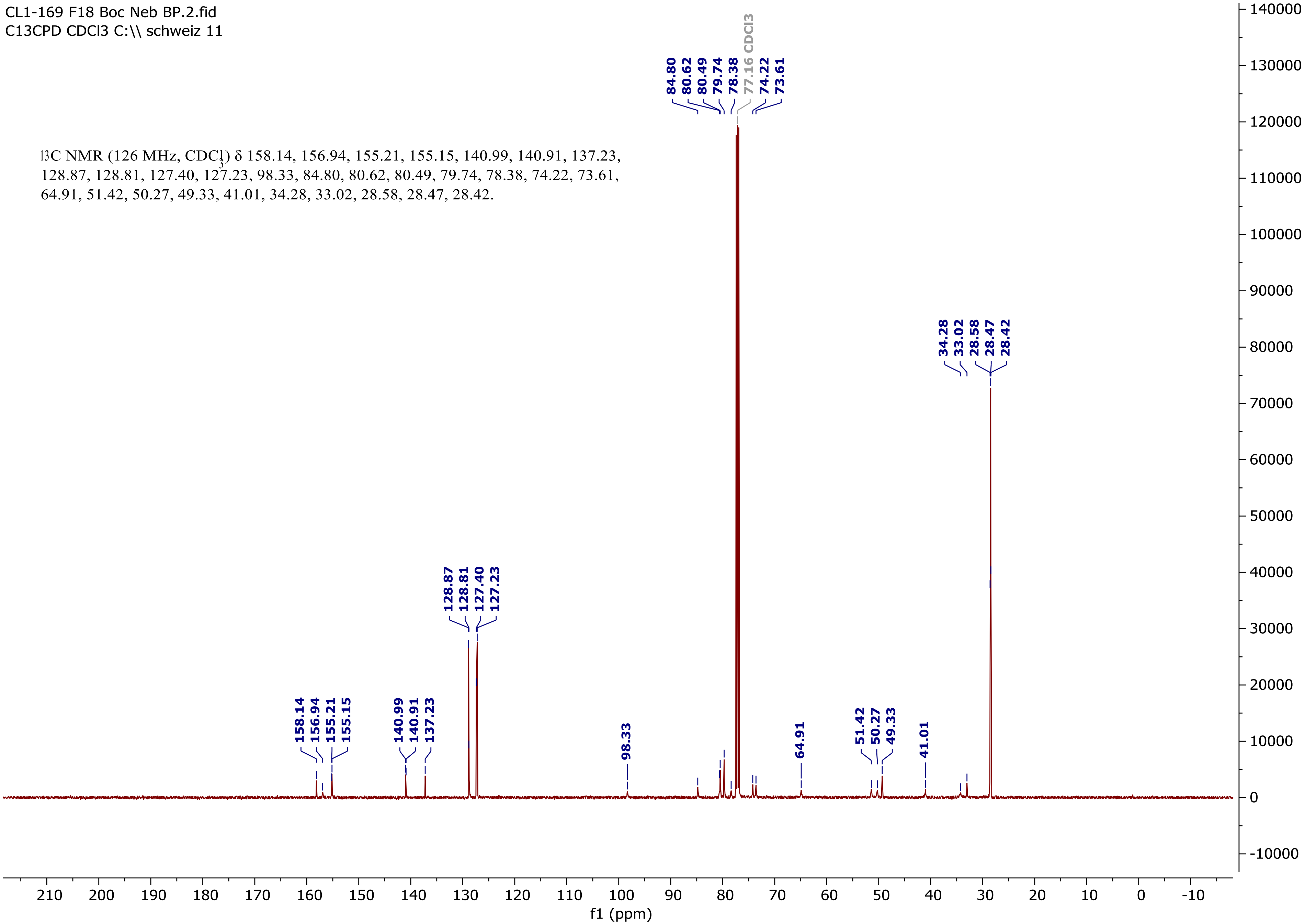

Figure S50. ^{13}C NMR spectrum for compound 5-O-(methylenebiphenyl)-1,3,2′,6′-tetra-N-Boc-Nebramine in $CDCl_3$.

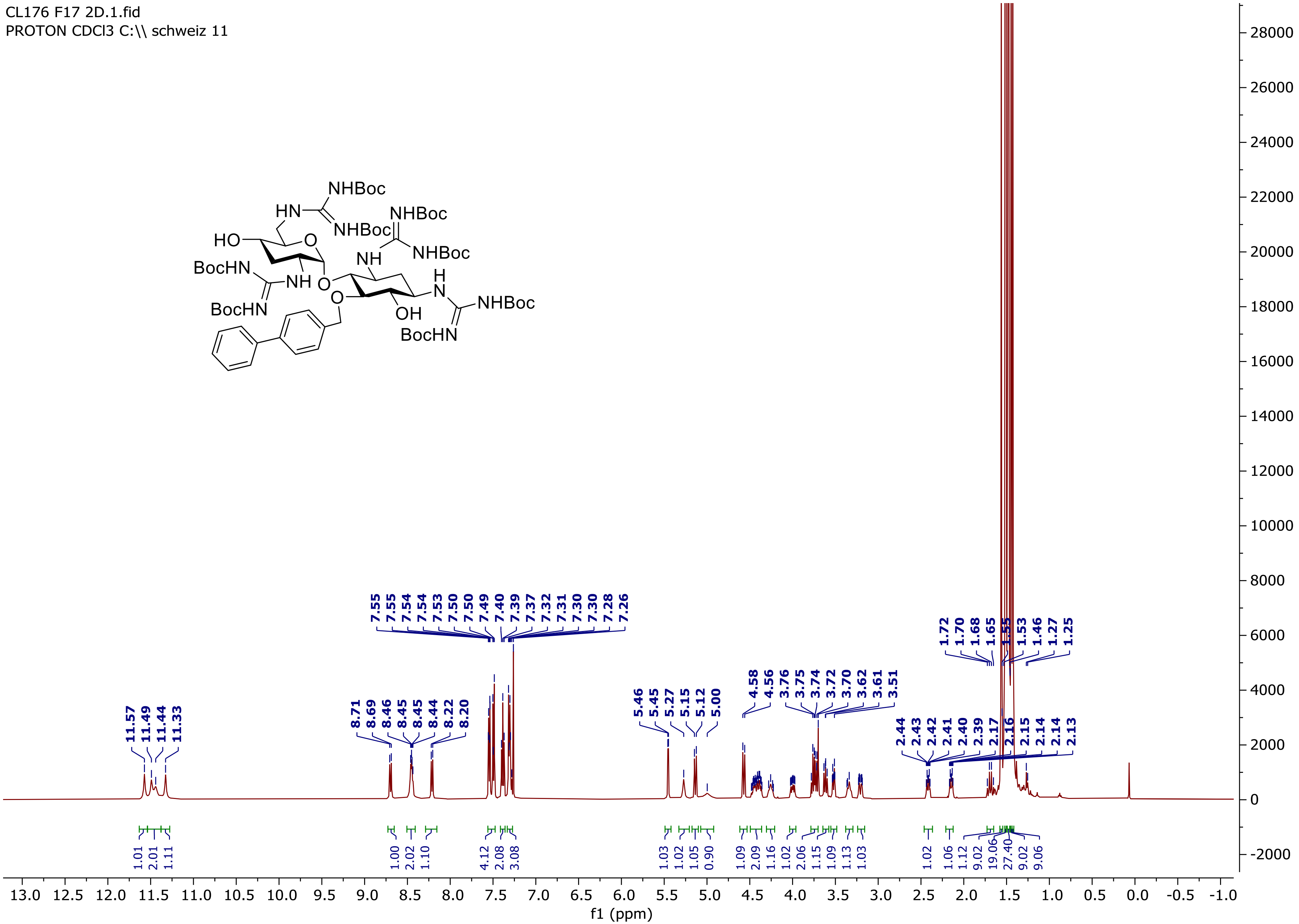

Figure S51. ^{1}H NMR spectrum for compound 5-O-(methylenebiphenyl)-Boc$_8$-Guanidino-Nebramine in $CDCl_3$.

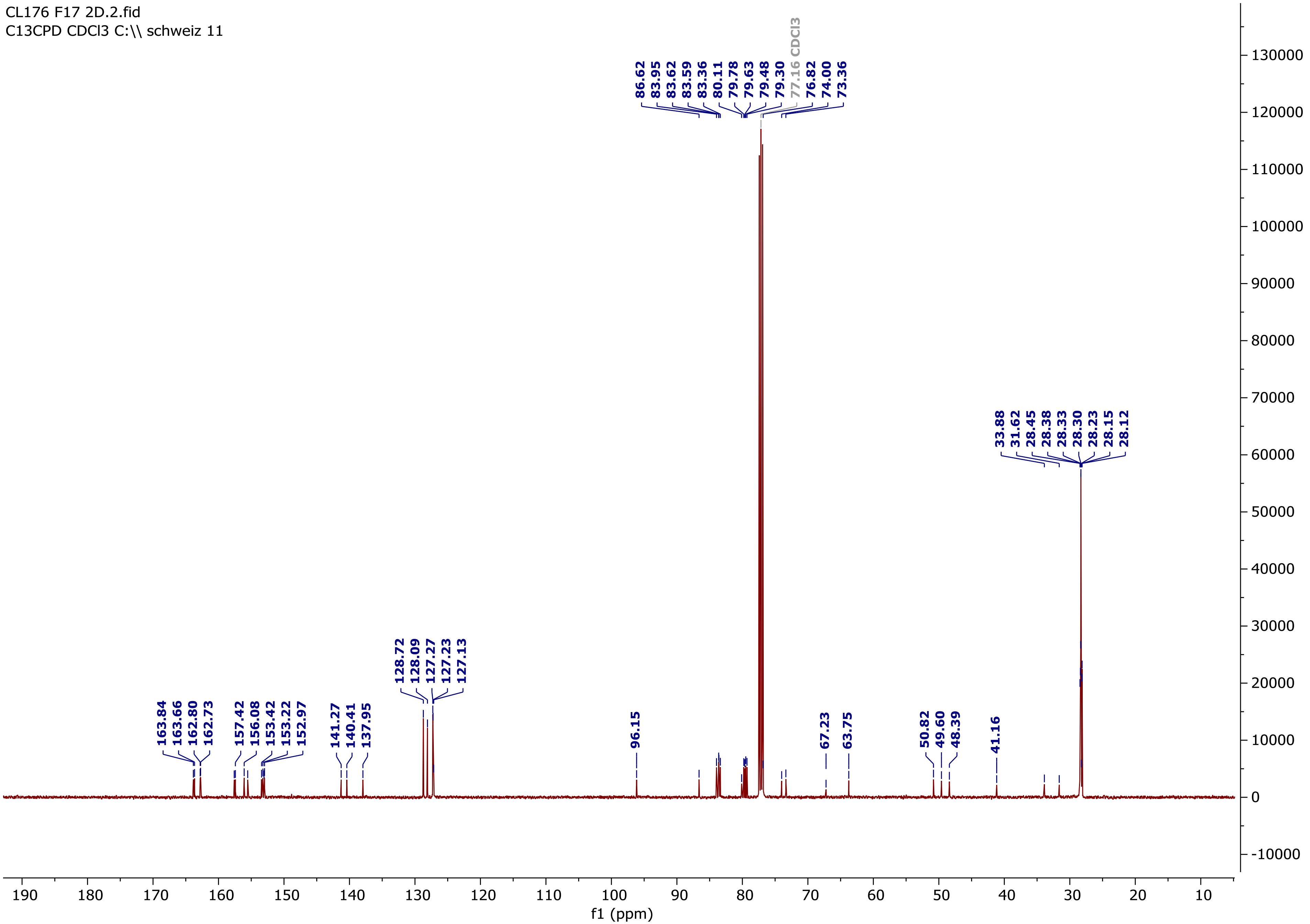

Figure S52. ^{13}C NMR spectrum for compound 5-O-(methylenebiphenyl)-Boc$_8$-Guanidino-Nebramine in $CDCl_3$.

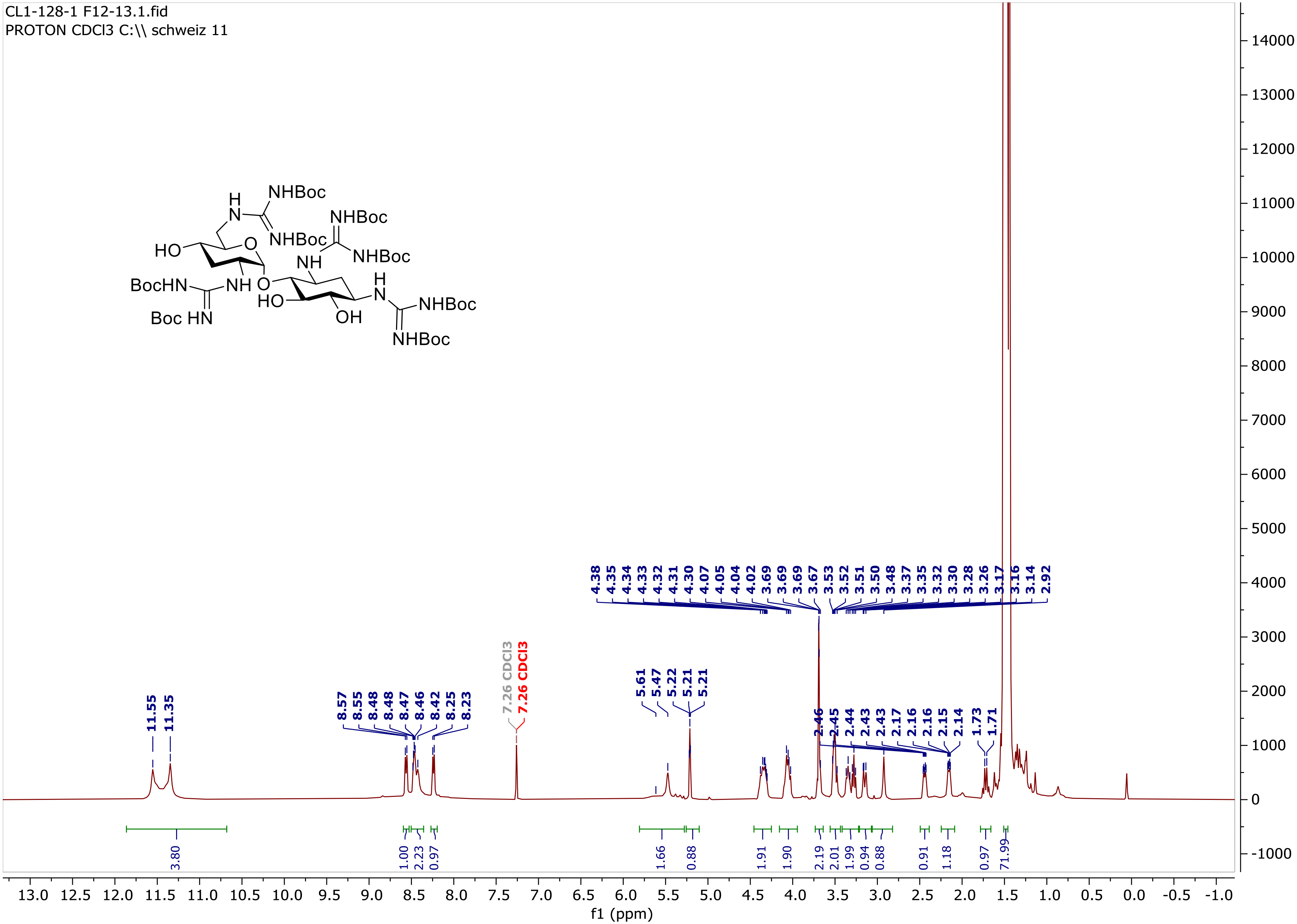

Figure S53. ^{1}H NMR spectrum for compound Boc$_8$-Guanidino-Nebramine in $CDCl_3$.

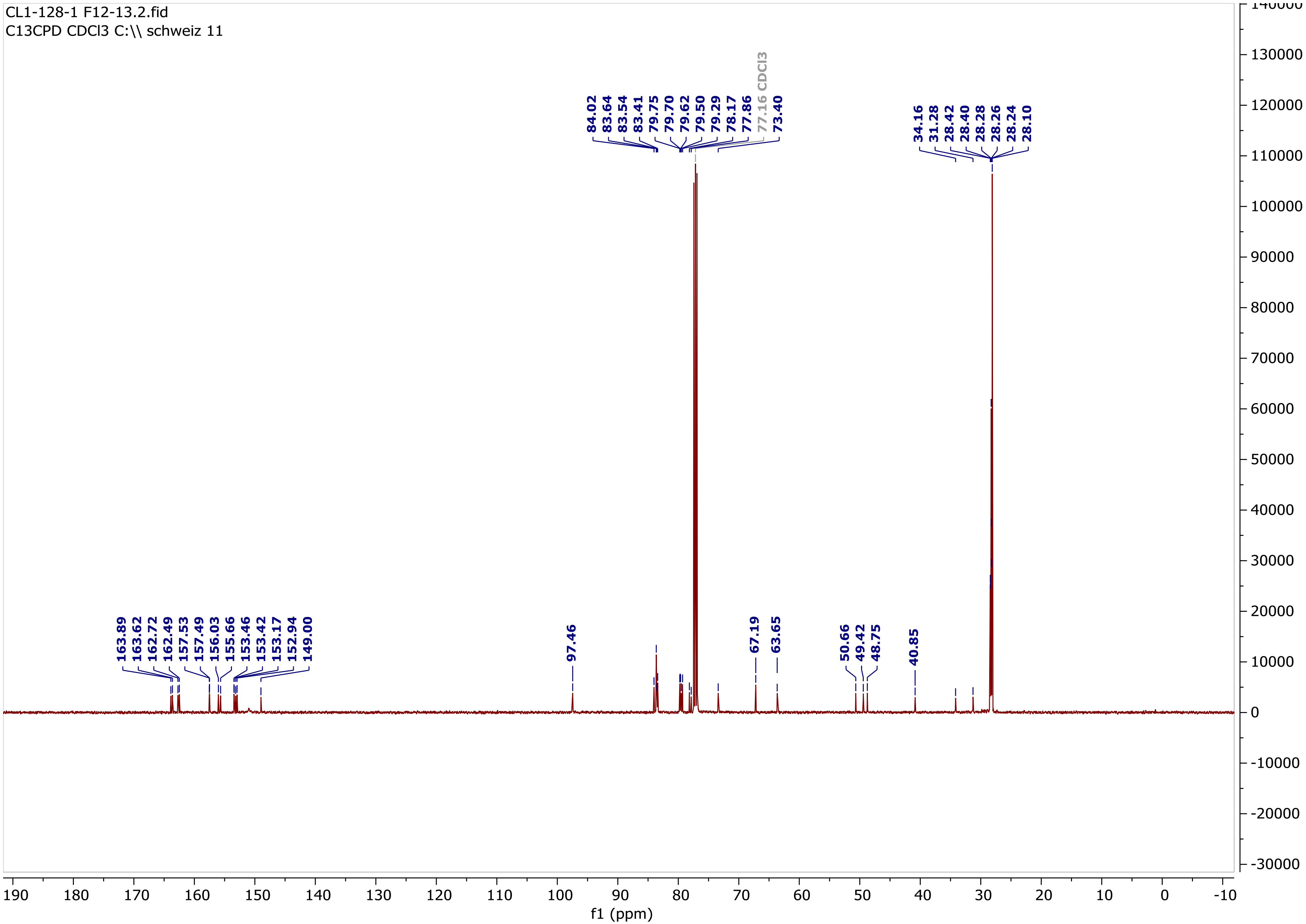

Figure S54. ^{13}C NMR spectrum for compound Boc$_8$-Guanidino-Nebramine in $CDCl_3$.

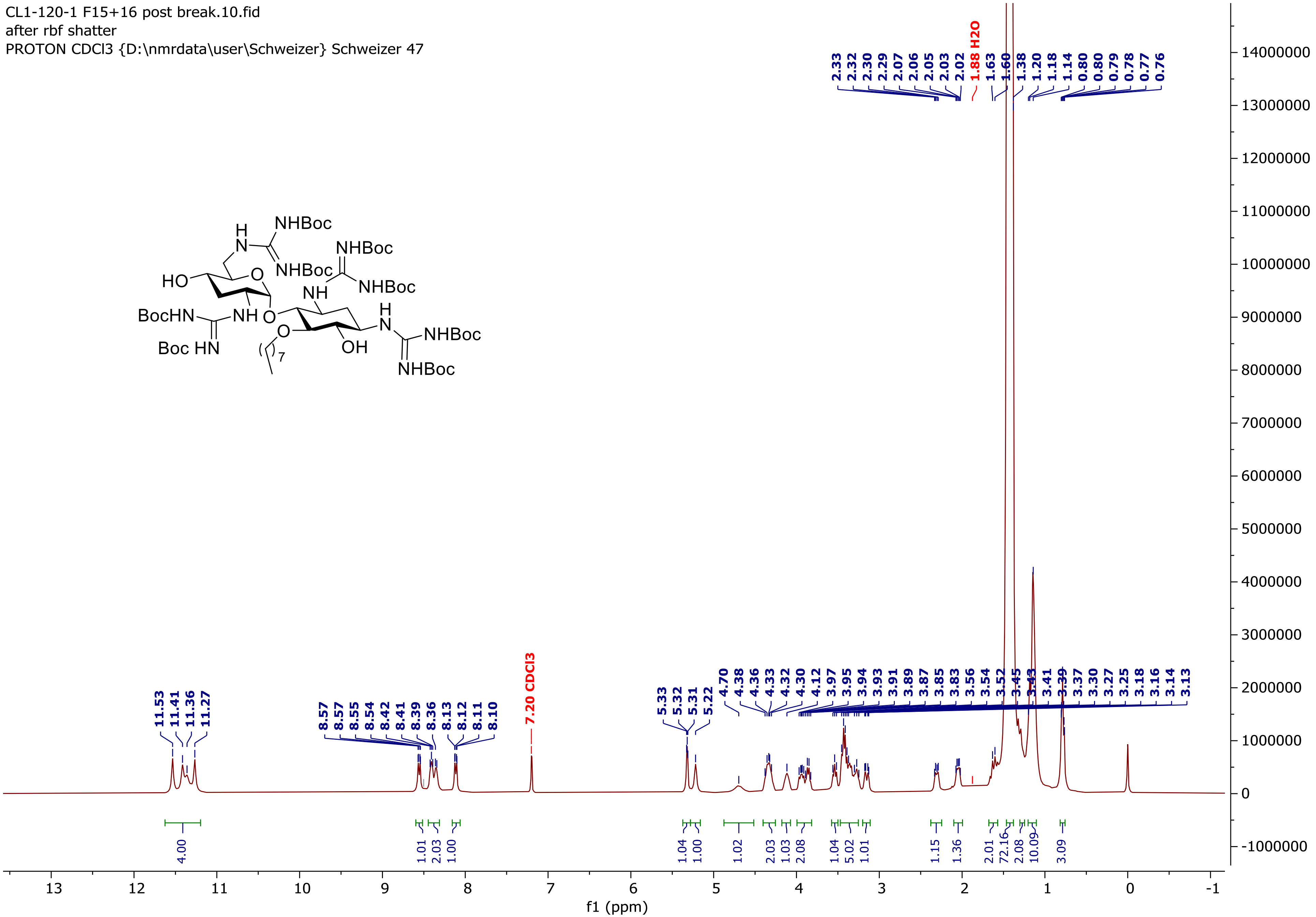

Figure S55. ^{1}H NMR spectrum for compound 5-O-(octyl)-Boc$_8$-Guanidino-Nebramine in $CDCl_3$.

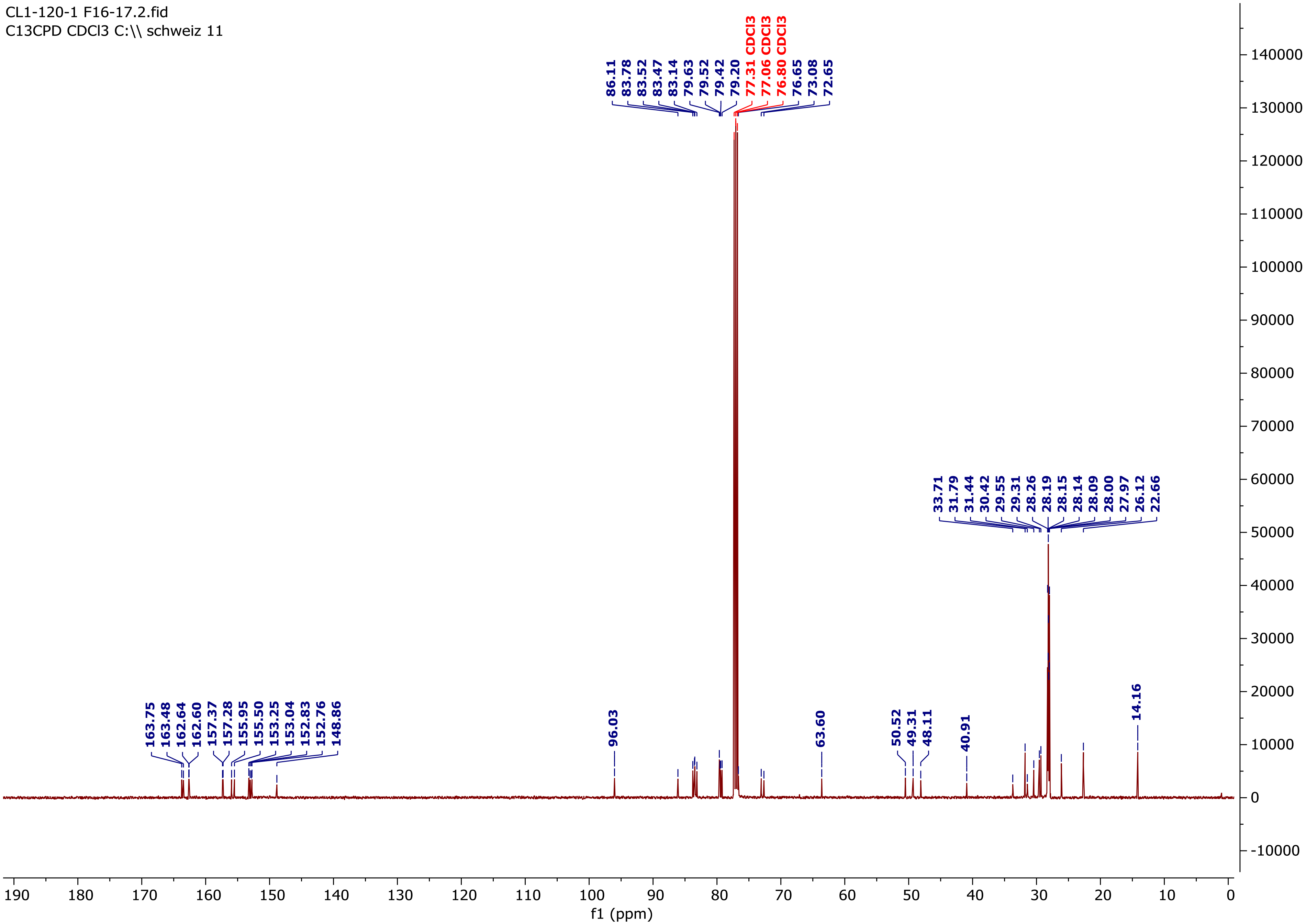

Figure S56. ^{13}C NMR spectrum for compound 5-O-(octyl)-Boc$_8$-Guanidino-Nebramine in $CDCl_3$.

1. Clinical and Laboratory Standards Institute, *Performance Standards for Antimicrobial Susceptibility Testing*, 35th ed., CLSI supplement M100, Table 2B–1, 2025.

2. Idowu, T.; Zhanel, G. G.; Schweizer, F., A dimer, but not monomer, of tobramycin potentiates ceftolozane against multidrug-resistant and extensively drug-resistant *Pseudomonas aeruginosa* and delays resistance development, *Antimicrob. Agents Chemother.*, **64** (2020), e02055-19.
